# Are the metal identity and stoichiometry of metal complexes important for colchicine site binding and inhibition of tubulin polymerization?†

**DOI:** 10.1039/d4dt01469c

**Published:** 2024-07-02

**Authors:** Iuliana Besleaga, Renáta Raptová, Alexandru-Constantin Stoica, Miljan N. M. Milunovic, Michal Zalibera, Ruoli Bai, Nóra Igaz, Jóhannes Reynisson, Mónika Kiricsi, Éva A. Enyedy, Peter Rapta, Ernest Hamel, Vladimir B. Arion

**Affiliations:** a Institute of Inorganic Chemistry, Faculty of Chemistry, University of Vienna Währinger Straße 42 A-1090 Vienna Austria miljan.milunovic@univie.ac.at vladimir.arion@univie.ac.at; b Institute of Physical Chemistry and Chemical Physics, Faculty of Chemical and Food Technology, Slovak University of Technology in Bratislava SK-81237 Bratislava Slovakia; c Institute of Physical and Theoretical Chemistry, Graz University of Technology Stremayrgasse 9/II A-8010 Graz Austria; d Inorganic Polymers Department, “Petru Poni” Institute of Macromolecular Chemistry Aleea Gr. Ghica Voda 41 A Iasi 700487 Romania; e Molecular Pharmacology Branch, Developmental Therapeutics Program, Division of Cancer Diagnosis and Treatment, National Cancer Institute, Frederick National Laboratory for Cancer Research, National Institutes of Health Frederick Maryland 21702 USA; f Department of Biochemistry and Molecular Biology, University of Szeged Közép fasor 52 H-6726 Szeged Hungary; g School of Pharmacy and Bioengineering, Keele University Newcastle-under-Lyme Staffordshire ST5 5BG UK; h Department of Molecular and Analytical Chemistry, Interdisciplinary Excellence Centre, University of Szeged Dóm tér 7-8 H-6720 Szeged Hungary enyedy@chem.u-szeged.hu; i MTA-SZTE Lendület Functional Metal Complexes Research Group, University of Szeged Dóm tér 7 H-6720 Szeged Hungary

## Abstract

Quite recently we discovered that copper(ii) complexes with isomeric morpholine-thiosemicarbazone hybrid ligands show good cytotoxicity in cancer cells and that the molecular target responsible for this activity might be tubulin. In order to obtain better lead drug candidates, we opted to exploit the power of coordination chemistry to (i) assemble structures with globular shape to better fit the colchicine pocket and (ii) vary the metal ion. We report the synthesis and full characterization of bis-ligand cobalt(iii) and iron(iii) complexes with 6-morpholinomethyl-2-formylpyridine 4*N*-(4-hydroxy-3,5-dimethylphenyl)-3-thiosemicarbazone (HL^1^), 6-morpholinomethyl-2-acetylpyridine 4*N*-(4-hydroxy-3,5-dimethylphenyl)-3-thiosemicarbazone (HL^2^), and 6-morpholinomethyl-2-formylpyridine 4*N*-phenyl-3-thiosemicarbazone (HL^3^), and *mono*-ligand nickel(ii), zinc(ii) and palladium(ii) complexes with HL^1^, namely [Co^III^(HL^1^)(L^1^)](NO_3_)_2_ (1), [Co^III^(HL^2^)(L^2^)](NO_3_)_2_ (2), [Co^III^(HL^3^)(L^3^)](NO_3_)_2_ (3), [Fe^III^(L^2^)_2_]NO_3_ (4), [Fe^III^(HL^3^)(L^3^)](NO_3_)_2_ (5), [Ni^II^(L^1^)]Cl (6), [Zn(L^1^)Cl] (7) and [Pd^II^(HL^1^)Cl]Cl (8). We discuss the effect of the metal identity and metal complex stoichiometry on *in vitro* cytotoxicity and antitubulin activity. The high antiproliferative activity of complex 4 correlated well with inhibition of tubulin polymerization. Insights into the mechanism of antiproliferative activity were supported by experimental results and molecular docking calculations.

## Abstract 2

The metal-to-ligand stoichiometry and metal identity are of primary importance for the development of metal complexes of thiosemicarbazones as effective inhibitors of tubulin polymerization.

## Graphical abstract

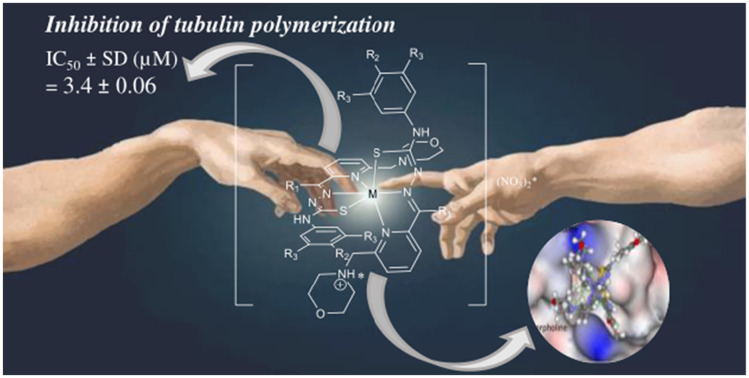

## Introduction

For several decades, Werner-type transition metal complexes have led drug research as alternatives to platinum-based cancer treatments.^1,2^ The success of such substances can be primarily attributed to features of their ligand-exchange kinetics.^3–5^ Metals play a significant role in the biological processes of the body, as the vital activities of the cell and enzymes are organized by their metal cofactors.^6,7^ Metal ions also serve as centers for building precise, three-dimensional constructs, offering various coordination geometries with unique stereochemistry. Such constructs are crucial for targeting DNA and intracellular proteins and inaccessible through carbon-based compounds alone.^8,9^ Complex formation with biologically active ligands often enhances antiproliferative activity in cancer cells.^10,11^ Understanding the distinct mechanisms behind antiproliferative activity is crucial for overcoming low selectivity and serious side effects of chemotherapy.^12–14^

Dynamic microtubules are validated primary targets in cancer therapy,^15^ and they play crucial roles in vital cellular processes, such as cell division, shape and motility, cell signaling and intracellular transport.^16–18^ Agents binding specifically to the colchicine site prevent tubulin from adopting a “straight” configuration that results in inhibition of microtubule assembly/disassembly, arrest of cell division and induction of cell death *via* apoptosis and/or necrosis. Several examples of first-row transition metal complexes acting as microtubule-destabilizing agents (MDA) or microtubule-targeting agents (MTA) have been reported.^19,20^ In addition, recently we described copper(ii) complexes with isomeric morpholine-thiosemicarbazone hybrids as the first transition metal complexes of thiosemicarbazones (TSCs) with a 1 : 1 metal-to-ligand ratio, and these complexes bind to tubulin in the colchicine site.^21^ Cu(ii) complexes featuring hybrid TSCs, with the morpholine moiety at each of the four available positions of the pyridine ring and a potentially redox active 2,6-dimethylphenol unit at the end nitrogen atom of the thiosemicarbazide fragment, exhibited significant anticancer activity against human uterine sarcoma MES-SA cells and the multidrug resistant derivative MES-SA/Dx5 cells, with IC_50_ values ranging from 1.4 μM to 13.1 μM. Notably, the compound bearing the morpholine moiety at position 6 of the pyridine ring exhibited the greatest antiproliferative activity (the lowest IC_50_ values) in the cancer cell lines and inhibited tubulin polymerization by binding to the colchicine site.

Given the 3D shape of the colchicine pocket in tubulin, we opted to exploit the power of coordination chemistry further in an attempt to build structures with well-defined globular shapes to complement the molecular diversity provided by purely organic scaffolds. This approach has not been used for the development of tubulin polymerization inhibitors so far, but it proved to be successful for creation of more efficient protein kinase inhibitors.^22,23^ We think attempts to assemble metal complexes with a more globular shape, when compared to 1 : 1 M-to-L complexes, to best fit the colchicine 3D pocket are worth exploring for the preparation of six-coordinate complexes of 1 : 2 M-to-L stoichiometry. Moreover, it would be worth investigating the impact of the central metal ion, the effect of coordination geometry, and the significance of the metal and ligand's potential redox activity on antiproliferative activity and on inhibition of microtubule assembly.

**Chart 1:**
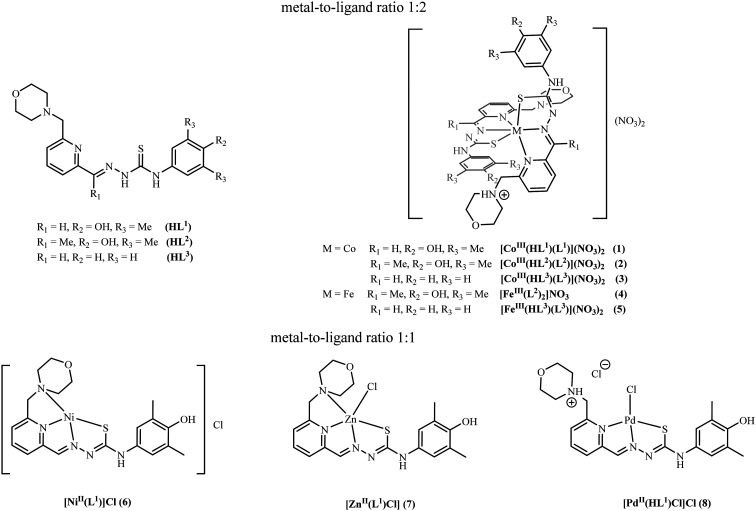
Line drawings of TSCs and their transition metal complexes studied in this work.

Thus, our goals were (i) the synthesis of new metal complexes with TSCs coupled with the morpholine moiety at position 6 of the pyridine ring and incorporating 2,6-dimethylphenol or a phenyl moiety of 1 : 2 and 1 : 1 metal-to-ligand stoichiometry (Chart 1); (ii) the investigation of the redox behavior and the stability of Co(iii) and Fe(iii) complexes of 1 : 2 metal-to-ligand stoichiometry in aqueous solution; (iii) the evaluation of the effects of the metal identity and metal-to-ligand stoichiometry (Chart 1) on *in vitro* antiproliferative activity and on tubulin polymerization; (iv) the elucidation of new structure–activity relationships; and (v) insights into the underlying mechanism of the antiproliferative activity consistent with the experimental data and molecular modelling.

## Results and discussion

### Synthesis and characterization of HL^1^–HL^3^

The synthesis of morpholine-TSC hybrids HL^1^ and HL^2^ was accomplished *via* Schiff base condensation reactions between 4*N*-(4-hydroxy-3,5-dimethylphenyl)-3-thiosemicarbazide^24^ and the corresponding aldehyde or ketone, respectively,^25,26^ while that of HL^3^ by reacting the aldehyde with 4-*N*-phenyl-3-thiosemicarbazide. These reactions were carried out in boiling ethanol, affording HL^1^–HL^3^ in good yields (>70%). The formation of HL^1^–HL^3^ was confirmed by ESI mass spectra, which showed peaks assigned to ions [HL + H]^+^ and [HL + Na]^+^, where HL = HL^1^–HL^3^. One- and two-dimensional NMR spectra were in agreement with the expected structures for HL^1^–HL^3^ of *C*_1_ molecular symmetry (Chart 1). The spectra of HL^1^–HL^3^ in DMSO-*d*_6_ indicated the presence of *E* and *Z*-isomers, typical for thiosemicarbazones, with a significant predominance of the *E*-isomers. This was demonstrated by the chemical shifts of the N^9^H proton (11.86 ppm for *E*-HL^1^ 10.43 ppm for *E*-HL^2^, 12.04 for *E*-HL^3^). For the atom labeling scheme for the NMR resonances assignment, see Chart S1 in the ESI.† The N^9^H proton of the *Z*-isomers was downfield-shifted due to the formation of a hydrogen bond with the pyridine nitrogen atom and resonated in the range *δ* 13.74–14.64 ppm. The *Z*-isomers were also identified by the hydrazinic nitrogen proton (N^11^H) with chemical shifts of 10.14 ppm (in *Z*-HL^1^), 9.97 ppm (in *Z*-HL^2^), and 10.51 ppm (in *Z*-HL^3^), compared to the *E*-isomers (9.98 ppm for HL^1^, 9.93 ppm for HL^2^, 10.24 ppm for HL^3^). The amount of *Z*-isomer was estimated as minor (less than 5%) by comparison of the integrals of the N^11^H protons in the spectra of both isomers (Fig. S1A and S3B in the ESI†). This type of isomerism is well-documented for similar TSCs and does not have any impact on their pharmacological profile.^27–29^

### Oxidation of TSCs

Semicarbazones and TSCs are prone to ring closure reactions in the presence of an oxidizing agent, to afford the corresponding 1,2,4-triazole and 1,3,4-oxa- or -thiadiazole derivatives.^29–31^ In the case of morpholine-TSC hybrids HL^1^ and HL^3^, it was found that the reaction of Fe(NO_3_)_3_·9H_2_O with these hybrids occurred *via* a 2-electron oxidative dehydrogenation, affording new species H*L*^1′^ and H*L*^3′^, containing a thiadiazole five-membered ring (for structure and atom labeling scheme see Chart S2 in the ESI†), as confirmed by ^1^H and ^13^C NMR spectra (Fig. S4A and S5B in the ESI†). This ring is formed *via* nucleophilic attack of the thione sulfur atom on the aldimine carbon atom with iron(iii) acting as an oxidant. It is of note that the more sterically hindered ketimine carbon atom in HL^2^ is less vulnerable to attack by the thione sulfur atom, and thiadiazole formation in the presence of iron(iii) was not observed. The crystal structure of thiadiazole derivative H*L*^1′^ in its protonated form, **[H**_**2**_***L***^**1**′^**]NO**_**3**_, was established by single crystal X-ray diffraction (SC-XRD) and is shown in Fig. S11 in the ESI.†

### Synthesis and characterization of complexes 1–8

Complexes 1–8 were obtained in good yields (57–92%) by the reactions of HL^1^–HL^3^ with the corresponding metal salts in methanol. Iron(iii) and cobalt(iii) complexes 1–5 were synthesized when starting materials were reacted in 1 : 2 molar ratio, while nickel(ii), zinc(ii) and palladium(ii) complexes 6–8 when metal salt and the HL^1^ were mixed in 1 : 1 molar ratio. The formation and purity of 1–8 were confirmed by ESI mass spectra, elemental analysis and ^1^H and ^13^C NMR spectra for diamagnetic complexes 1–3, 7 and 8 (see Fig. S6A and S10B in the ESI†). The d^8^ electronic configuration of Ni(ii) and Pd(ii) favors square-planar coordination geometry, while Zn(ii) with completely filled d-orbitals adopts a square-pyramidal geometry. In positive ion ESI mass spectra, Fe(iii) and Co(iii) bis-ligand complexes revealed a diagnostic peak due to [M(L^1^)_2_]^+^–[M(L^3^)_2_]^+^ ions, while complexes with 1 : 1 metal-to-ligand ratio showed peaks assigned to [M(L^1^)]^+^ ions (M = Ni(ii), Zn(ii), Pd(ii)).

The ^1^H NMR spectra of cobalt(iii) complexes 1–3 in DMSO-*d*_6_ (Fig. S6A and S8B in the ESI†) are consistent with deprotonation of the N^9^H upon TSC coordination to metal ions, as the peaks at 11.86 ppm for HL^1^, 10.43 ppm for HL^2^, 12.04 ppm for HL^3^ disappeared. The mentioned proton resonances were also missing in the ^1^H NMR spectra of the zinc(ii) and palladium(ii) complexes 7 and 8 (Fig. S9A and S10B in the ESI†). In the spectra of 1 and 7, the singlet of the azomethine proton CH=N is upfield shifted compared to the free ligand, suggesting the coordination to the metal ion.

Despite its square-planar coordination geometry in the solid state, complex 6 is paramagnetic in DMSO-*d*_6_. The determined magnetic moment by the Evans method^32^ at room temperature (*μ*_eff_ = 2.83*μ*_B_) is in agreement with the presence of 2 unpaired electrons in the d-orbitals (*S* = 1). Axial coordination of two DMSO molecules is likely.

X-ray diffraction quality single crystals of 1, 4, 5, 6 and 8 were obtained by re-crystallization in methanol, while 3 and 7 upon vapor diffusion of diethyl ether into their methanolic solutions.

### X-ray diffraction study of the proligands and their complexes

**Fig. 1:**
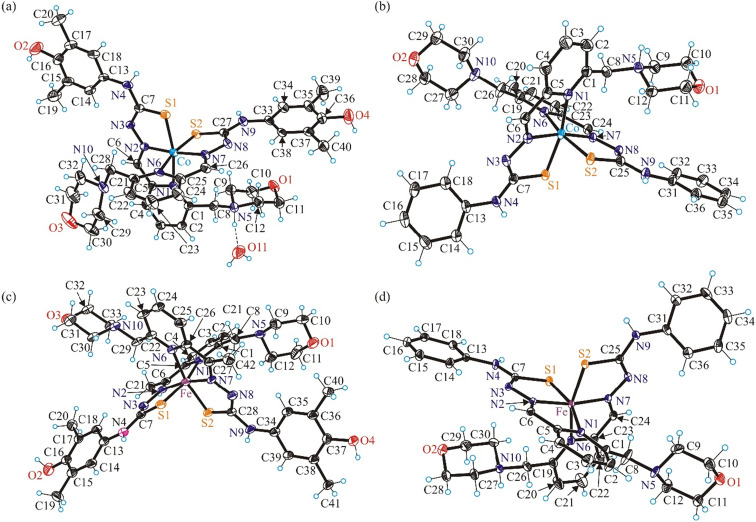
ORTEP views of the metal complex cations in (a) [Co^III^(HL^1^)(L^1^)](NO_3_)_2_·H_2_O (1), (b) [Co^III^(HL^3^)(L^3^)](NO_3_)_2_ (3), (c) [Fe^III^(L^2^)_2_]NO_3_ (4) and (d) [Fe^III^(HL^3^)(L^3^)](NO_3_)_2_ (5) with thermal ellipsoids at the 50% probability level.

**Fig. 2:**
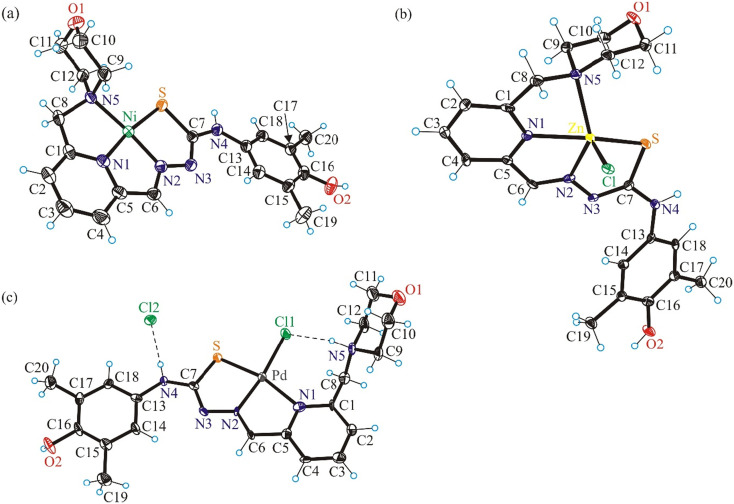
ORTEP views of (a) [Ni^II^(L^1^)]Cl·CH_3_OH (6), (b) [Zn^II^(L^1^)Cl]·CH_3_OH (7) and (c) [Pd^II^(HL^1^)Cl]Cl (8) with thermal ellipsoids at the 50% probability level (check). Interstitial solvent molecules were omitted for clarity.

The results of SC-XRD studies of complexes 1 and 3–8, with the atom labeling schemes, are shown in Fig. 1 and 2. The Co(iii)- and Fe(iii) bis-ligand complexes 1 and 3–5 crystallized in the centrosymmetric monoclinic space group(s) *P*2_1_/*c* and *P*2_1_/*n*, respectively, as racemic mixtures of the two enantiomers of chiral six-coordinate Co(iii) and Fe(iii) complexes (Fig. 1),^33^ as also reported for other metal bis-thiosemicarbazonates. Spontaneous resolution of these kinds of complexes, governed by the cooperative effect of H-bonding and π–π stacking interactions, was described previously for Mn^II^L_2_, with HL = acetylpyrazine thiosemicarbazone.^34^

The potentially tetradentate ligands in these complexes act as tridentate. The morpholine moiety is not involved in coordination to Co(iii) and/or Fe(iii). In all four cases the ligands are coordinated to the metal *via* the pyridine nitrogen atom, hydrazinic nitrogen atom and thiolato sulfur atom. However, in complexes 1, 3 and 5, one thiolato ligand from the two coordinated to the metal is protonated at the nitrogen atom of the morpholine moiety, adopting a zwitterionic form. These ligands are considered charge neutral (HL^1^ or HL^3^). Protonation of one of the two morpholine moieties is also corroborated by the presence of hydrogen bonding interactions between the protonated morpholine moiety as proton donor and proton acceptor groups, *e.g.*, water oxygen atom O11 in 1 (see Fig. 1).

The metrical parameters for complexes 1, 3 and 4, 5 summarized in Table 1 are comparable to those for Co(iii)^35–37^ and iron(iii) complexes,^33,38–40^ respectively, with related TSCs.

**Table 1:** Selected bond lengths (Å) and bond angles (deg) around M(iii) ion (M = Co, Fe) in complexes 1, 3–5

Complex	1	3	4	5
M–N1	2.107(2)	2.064(2)	2.086(5)	2.0797(11)
M–N2	1.876(3)	1.8961(18)	1.914(5)	1.9250(11)
M–S1	2.2045(9)	2.2084(9)	2.2057(17)	2.2094(4)
M–N6	2.074(2)	2.0434(17)	2.071(4)	2.0755(13)
M–N7	1.890(3)	1.8997(17)	1.896(4)	1.9244(11)
M–S2	2.2146(9)	2.2194(7)	2.1996(14)	2.2021(5)
N1–M–N2	81.58(10)	82.09(8)	89.06(11)	80.35(4)
N2–M–Sl	85.00(8)	84.95(6)	84.90(15)	84.40(3)
N6–M–N7	82.86(11)	82.22(7)	80.91(17)	80.67(5)
N7–M–S2	84.67(8)	84.89(6)	85.12(12)	84.09(4)

The Ni(ii), Zn(ii) and Pd(ii) complexes 6–8 with metal-to-ligand stoichiometry 1 : 1 (Fig. 2) crystallized in the noncentrosymmetric orthorhombic space group *Pnn*2 (6), in the centrosymmetric triclinic space group *P*1̄ (7) and orthorhombic space group *I*2/*c* (8) Selected bond lengths and bond angles for 6–8 are collected in Table 2. The HL^1^ acts as monoanionic tetradentate ligand in Ni(ii) and Zn(ii) complexes 6 and 7, respectively. The coordination occurs *via* pyridine nitrogen atom N1, hydrazinic nitrogen N2, thiolato sulfur atom S and nitrogen atom N5 of the morpholine moiety. The calculated *τ*′_4_-parameter is 0.15,^41^ in agreement with slightly distorted square-planar coordination geometry of Ni(ii) in 6. The coordination polyhedron of Zn(ii) in 7 is completed by a chlorido co-ligand and is best described as slightly distorted square-pyramidal. The *τ*_5_-parameter for Zn(ii) in 7 is 0.10.^42^ The morpholine moiety in all complexes studied by SC-XRD (1, 3–8) adopts the chair conformation.

**Table 2:** Selected bond lengths (Å) and bond angles (deg) around central M(ii) ion (M = Ni, Zn, Pd) in complexes 6–8

Complex	6	7	8
M–N1	1.826(4)	2.104(4)	2.131(5)
M–N2	1.848(4)	2.158(3)	1.944(4)
M–S	2.1470(14)	2.3490(12)	2.2232(14)
M–N5	1.973(4)	2.288(3)	
M–Cl		2.3196(14)	2.3183(16)
N1–M–N2	83.67(18)	73.10(13)	80.4(2)
N2–M–S	87.33(13)	79.86(10)	84.95(14)
N1–M–N5	84.62(18)	74.75(12)	
N2–M–N5	166.12(17)	140.18(14)	
N1–M–S	171.00(15)	146.33(12)	165.06(15)
N2–M–Cl			172.24(14)

In 8 the ligand adopts a zwitterionic form being deprotonated at the thiolato sulfur atom and protonated at the nitrogen atom N5 of the morpholine moiety. Moreover, in contrast to 6 and 7, the ligand in 8 acts as a tridentate one binding to Pd(ii) *via* pyridine nitrogen atom N1, hydrazinic nitrogen N2 and thiolato sulfur atom S. The square-planar coordination geometry is completed by additional coordination of one chlorido co-ligand (Cl1) as shown in Fig. 2c, while Cl^−^ (Cl2) acts as the counteranion. The calculated *τ*′_4_-parameter is 0.14.^41^ The atom N4 is a proton donor in a H-bond to chloride counteranion (Cl1 as proton acceptor), while N5 is a proton donor to the chlorido co-ligand. The bond lengths in the first coordination sphere of Pd(ii) are in good agreement with those documented for Pd(ii) complexes with related tridentate TSCs.^43,44^

### Solution stability studies

The stability of the proligands HL^1^–HL^3^ in both dimethyl sulfoxide (DMSO) and aqueous solution at various pH values was monitored over time by UV–visible (UV–vis) spectrophotometry. The compounds were stable in both DMSO and water at pH 7.4, as the spectra remained unchanged over 24 h (Fig. S12 in the ESI†). However, slow spectral changes were observed under both acidic (pH 2) and basic (pH 11.7) conditions. The most remarkable changes were for HL^2^ at pH 2 (Fig. S12 and S13 in the ESI†). The decrease of the absorbance band at 310 nm is most likely due to the acid-catalyzed cleavage of the C=N Schiff base bond, resulting in a less extended conjugation system in the molecule or desulfurization, which is likely under acidic conditions.^45,46^ The much more extensive hydrolysis of TSCs with a methyl group at the azomethine carbon was reported previously for 2-acetylpyridine TSCs.^47^ Spectral changes observed at pH 11.7 are likely due to Schiff base hydrolysis as well, although, in the case proligands containing the redox-active 2,6-dimethylphenol group (HL^1^ and HL^2^), oxidation may also occur, as was reported for triapine analogues.^29^ To obtain further evidence for oxidation of the phenolic moiety, time-dependent measurements were performed under strictly anaerobic conditions in a glove box (Fig. S12 in the ESI†). For proligands HL^1^ and HL^2^, the spectral changes were undoubtedly less significant when air oxygen was excluded, suggesting that these compounds can indeed be oxidized at the highly basic pH values.

Therefore, the proton dissociation constants (p*K*_a_) of the proligands HL^1^–HL^3^ were determined under anoxic conditions by pH-potentiometric titrations (Fig. S14 in the ESI†). As this method requires relatively high compound concentrations (>1 mM), the measurements were performed in 30% (v/v) DMSO to achieve the required solubility. Three p*K*_a_ values were determined for the proligands HL^1^ and HL^2^, whereas only two constants, as expected, were obtained for phenyl derivative HL^3^ (Table 3). The constants obtained were assigned to the deprotonation N_morpholinium_H^+^ (N_morph_H^+^), N_hydrazine_H (N_hydr_H) and OH moieties for HL^1^ and HL^2^, and to the N_morph_H^+^ and N_hydr_H for HL^3^, respectively. It should be noted that we could not determine p*K*_a_ values for the pyridinium group as its deprotonation in all three instances took place at fairly low pH values (pH < 2) due to the electron withdrawing effect of the protonated morpholinium moiety. The determined p*K*_a_ values indicate that these compounds are in their neutral HL form at pH 7.4 and only a minor fraction (1–3%) is protonated ((HL)H^+^) at the morpholinium nitrogen (Table 3).

**Table 3:** p*K*_a_ values of the proligands HL^1^–HL^3^ determined by pH-potentiometric titrations in 30% (v/v) DMSO/H_2_O {*t* = 25.0 °C; *I* = 0.1 M (KCl)} in addition to the distribution of the ligand species in the different protonation states at pH 7.4 and pH 6.0. Log *D* values obtained by *n*-octanol/water partitioning at pH 6.0 (20 mM 2-morpholinoethanesulfonic acid (MES), *t* = 25.0 °C)

	HL^1^	HL^2^	HL^3^
p*K*_a_ (N_morph_H^+^)	5.54 ± 0.03	5.85 ± 0.04	5.55 ± 0.02
p*K*_a_ (N_hydr_H)	10.44 ± 0.03	10.41 ± 0.03	10.44 ± 0.03
p*K*_a_ (OH)	12.12 ± 0.02	12.23 ± 0.02	—
Molar fraction	1% HL(H)^+^	3% HL(H)^+^	1% HL(H)^+^
pH 7.4	99% HL	97% HL	99% HL
Molar fraction	26% HL(H)^+^	43% HL(H)^+^	27% HL(H)^+^
pH 6.0	74% HL	57% HL	73% HL
log *D*_6.0_	+1.23 ± 0.10	+1.50 ± 0.05	+2.06 ± 0.03

Attempts to determine the distribution coefficients (log *D*) of the proligands HL^1^–HL^3^ using the shake-flask method in an *n*-octanol/buffered aqueous solution at pH 7.4 showed that the compounds remained mostly in the *n*-octanol phase, implying their fairly lipophilic character (log *D*_7.4_ > 2). Therefore, the experiment was also conducted at pH 6, at which the fraction of the protonated species (HL)H^+^ is higher (Table 3). The log *D*_6.0_ values give the following trend: HL^3^ > HL^2^ > HL^1^. Interestingly, the 2,6-dimethylphenol moiety enhances hydrophilicity, while the methyl substituent at the Schiff base azomethine carbon atom modestly increases lipophilicity.

The stability of the Co(iii) and Fe(iii) complexes (1, 2, 4 and 5) in DMSO and in water at pH 2.0 (see representative spectra for complex 2 in Fig. S15a in the ESI†), 7.4 and 11.7 was also monitored over time by UV–vis spectrophotometry. Both Co(iii) complexes were found to be stable in DMSO and in water at pH 2.0 and 7.4 for 24 h. However, slow spectral changes were observed at pH 11.7 in the presence of O_2_, most likely due to the oxidation of the 2,6-dimethylphenol moiety, because the spectra remained unchanged if the measurements were performed under anoxic conditions. In contrast, complete dissociation of iron(iii) complexes 4 and 5 was found at pH 2, as the measured spectra were very similar to those of the free proligands (Fig. S15b for 5 in the ESI†). Spectral changes recorded in both DMSO and in water at pH 7.4 indicated the slow partial dissociation of the complexes (Fig. S15c and S15d for 5 in the ESI†). On the other hand, at pH 11.7 under anoxic conditions, the changes were minimal (Fig. S16 for 4 in the ESI†), except for the appearance of an absorption band with *λ*_max_ ∼ 650 nm, which is typical for the Fe(ii) complexes of α-*N*-pyridyl TSCs.^48^ Due to the kinetic inertness of the Co(iii) complexes (d^6^, *S* = 0) and slow dissociation and reduction of the Fe(iii) complexes at pH 7.4 no titrations were performed to determine their solution speciation. The lipophilicity of the Co(iii) complexes characterized at pH 6.0 (log *D*_6.0_ = +0.94 ± 0.03 for 1, and +1.37 ± 0.05 for 2) shows that the complexes are slightly more hydrophilic than the corresponding ligands.

**Fig. 3:**
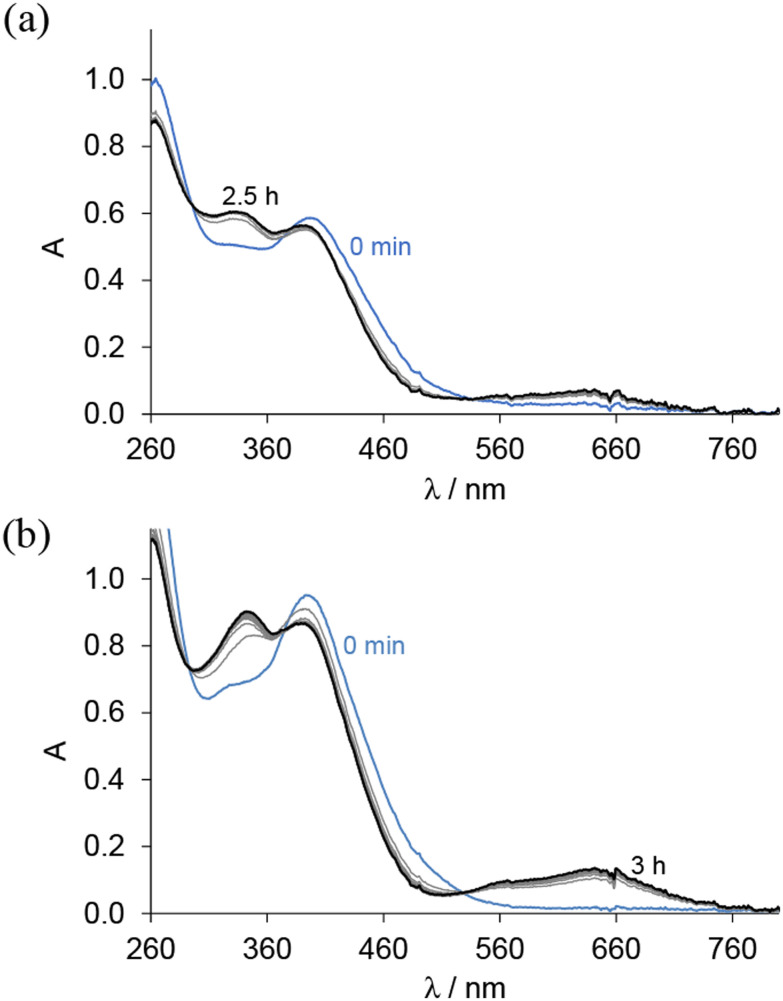
UV–vis absorption spectra for (a) complex 4 and (b) complex 5 in the presence of 100 equiv. GSH at pH 7.4 measured over time in 30% (v/v) DMSO/H_2_O; {*c*_complex_ = 25 μM; *ℓ* = 1 cm; *T* = 25.0 °C}.

The reduction of complexes 1, 2, 4 and 5 by glutathione (GSH), one of the major cellular reductants, was investigated at pH 7.4 by recording UV–vis spectra in a glove box. The spectra of the Co(iii) complexes remained unchanged upon the addition of GSH, indicating that no redox reaction occurred. In contrast, for Fe(iii) complexes, the appearance of the typical charge transfer bands of Fe(ii) species at *λ* > 560 nm suggests their reduction by GSH (Fig. 3).

The redox properties of the Co(iii) and Fe(iii) complexes were further investigated by cyclic voltammetry and UV–vis–NIR spectroelectrochemical measurements.

### Cyclic voltammetry and spectroelectrochemistry

**Fig. 4:**
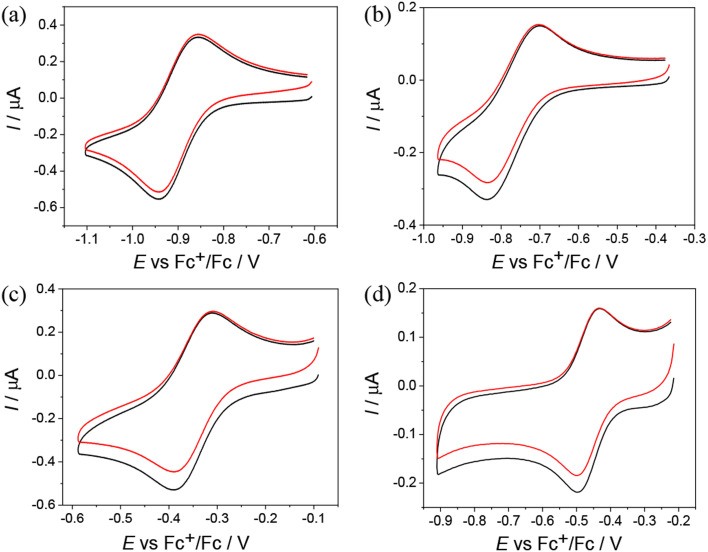
CVs of (a) Co(iii) complex 2, (b) Co(iii) complex 3, (c) Fe(iii) complex 4, and (d) Fe(iii) complex 5 in DMSO/*n*Bu_4_NPF_6_ (Pt working electrode, scan rate: 100 mV s^−1^).

Cyclic voltammograms (CV) of Co(iii) and Fe(iii) complexes 1–5 with metal-to-ligand ratio 1 : 2 showed the reversible first reduction peak in the cathodic part in DMSO/*n*Bu_4_NPF_6_ when using platinum or glassy-carbon working electrodes at a scan rate of 100 mV s^−1^ (Table 4 and Fig. 4).

**Table 4:** Electrochemical data^a^ for 1–8 in *n*Bu_4_NPF_6_/DMSO

Complex	1^st^ reduction	2^nd^ reduction	1^st^ oxidation
1	−0.78^q^	−1.05^q^	0.43^i^*
2	−0.90^r^	−1.32^q^	0.48^i^*
3	−0.77^r^	−1.08^q^	
4	−0.35^r^	−1.85^q^	0.40^i^*
5	−0.47^r^	−0.98^q^	
6	−1.29^q^	−1.87^q^	0.12^i^*
7	−1.86^i^		0.24^i^*
8	−0.91^q^	−1.28^q^	0.28^i^*

a Half-wave potentials *E*_1/2_ or peak potentials (*) *E*_p_, in volts *vs.* Fc^+^/Fc, scan rate 100 mV s^−1^; r – reversible, q – quasireversible, i – irreversible.

The Co(iii) complexes 1–3 exhibit the most negative reduction potentials from −0.77 to −0.90 V (Table 4). The least negative reduction potential was observed for the Fe(iii) complex 4, indicating easy and reversible reduction, while the reduction of Fe(iii) complex 5 occurs at a more negative potential (see Table 4). The cyclic voltammetry of metal complexes of 1 : 1 stoichiometry showed less reversible reduction events in the region of the first electron transfer, and these were much more negative. For the Ni(ii) complex 6, two quasireversible reduction waves were observed at −1.29 and −1.87 V *vs.* Fc^+^/Fc (Fig. S17a in the ESI†). Given the redox inactivity of the corresponding proligand in the cathodic part, a strong influence of the central atom on the ligand and a substantially noninnocent character of the ligand are conceivable. In the anodic part, one fully irreversible peak appeared upon oxidation at *E*_pa_ = 0.12 V *vs.* Fc^+^/Fc with about double intensity compared to the reduction peaks (Fig. S17b in the ESI†). This wave could be attributed to the 2-electron oxidation of the potentially redox active 2,6-dimethylphenol unit.^29^ The Zn(ii) complex 7 is irreversibly reduced at −1.86 V *vs.* Fc^+^/Fc. This reduction is presumably ligand-based (Fig. S17c in the ESI†). An irreversible peak was observed at *E*_pa_ = 0.24 V *vs.* Fc^+^/Fc in the anodic part, which could be assigned to the oxidation of the 2,6-dimethylphenol unit (Fig. S17d in the ESI†). For Pd(ii) complex 8, a quasireversible reduction peak was detected at −1.28 V *vs.* Fc^+^/Fc, followed by several consecutive reduction peaks at higher potentials, indicating irreversible changes after reduction. Irreversible oxidation occurred at *E*_pa_ = 0.28 V *vs.* Fc^+^/Fc, which is presumably localized on the ligand (Fig. S18 in the ESI†).

**Fig. 5:**
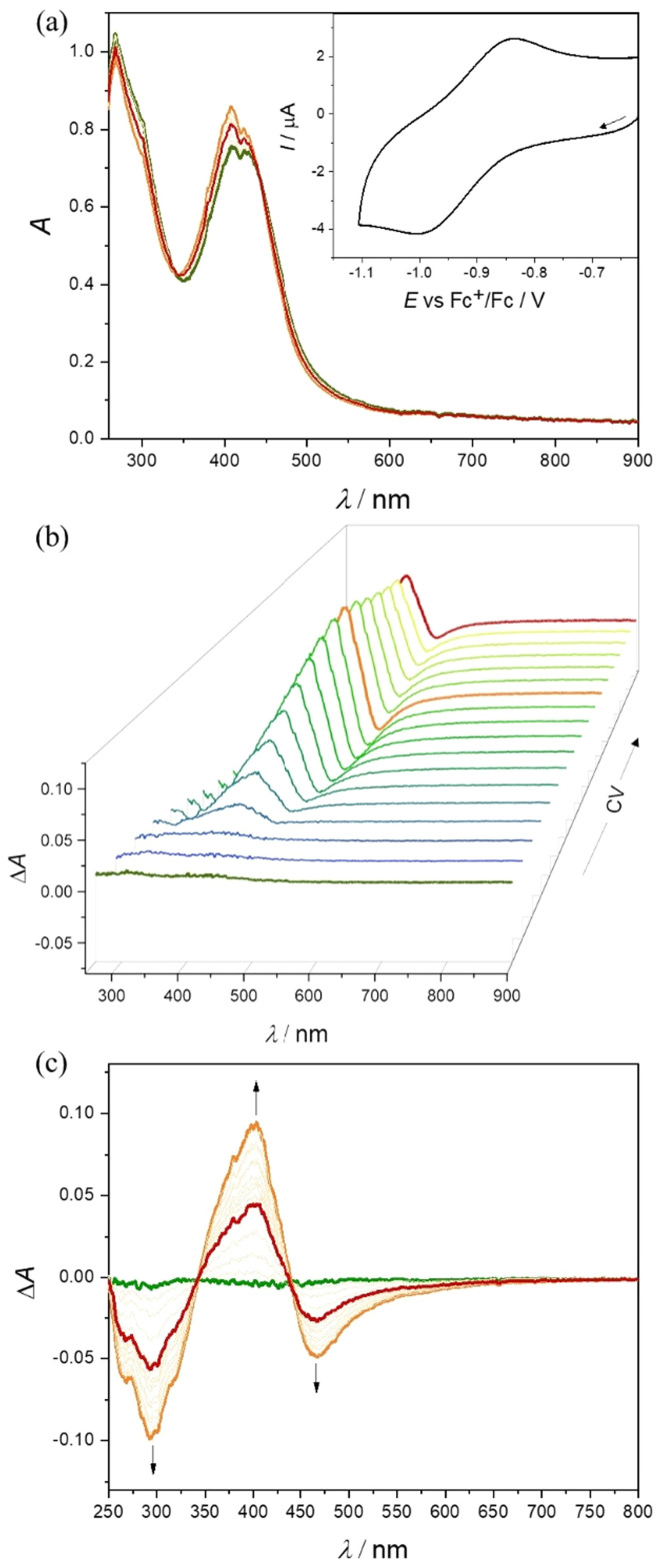
Spectroelectrochemistry of 2 in *n*Bu_4_NPF_6_/DMSO in the region of the first cathodic peak: (a) UV–vis–NIR spectra measured simultaneously with *in situ* reduction; inset: CV of 2 with Pt-microstructured honeycomb working electrode and scan rate 10 mV s^−1^; (b) 3D-difference UV–vis–NIR spectra measured simultaneously with CV and (c) 2D-difference Δ*A* optical spectra.

**Fig. 6:**
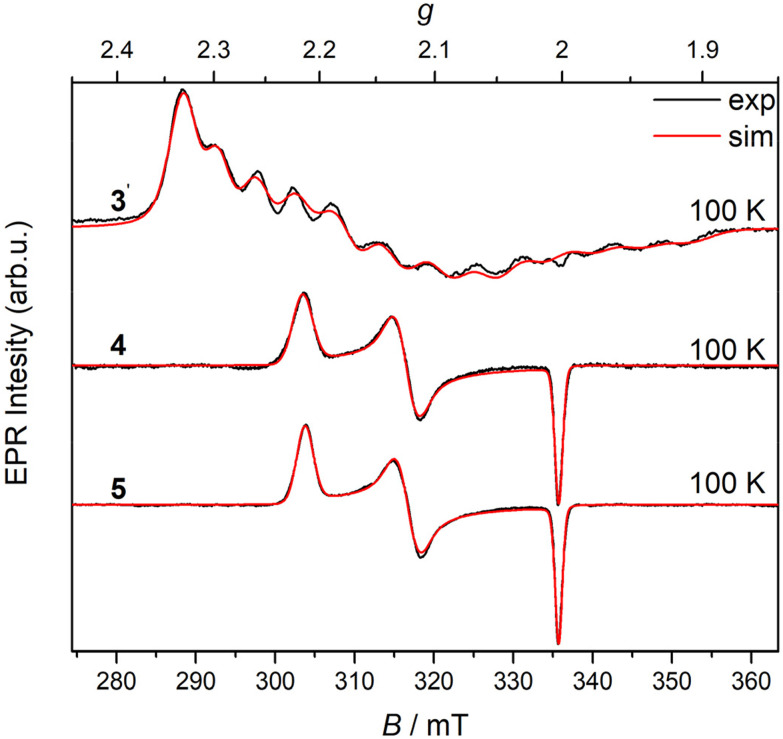
X-band EPR spectra of 4, 5 and 3 reduced by cobaltocene (3′) in acetonitrile/dimethylformamide (MeCN/DMF) glass at 100 K. The black traces show experimental records, and the red traces show the simulations using Spin-Hamiltonian parameters quoted in the text. Spectrometer settings: microwave frequency, 9.45 GHz; microwave power, 1 mW; modulation frequency, 100 kHz; modulation amplitude, 0.2 mT.

No EPR signal was detected at room temperature nor at 100 K for Co(iii) complexes 1–3, indicating the low spin EPR inactive configuration of the central atom (d^6^, *S* = 0). Applying *in situ* UV–vis–NIR spectroelectrochemistry, the nearly reversible behavior in the cathodic part for 2 and 3 in *n*Bu_4_NPF_6_/DMSO was observed. As very small changes have been detected during *in situ* voltammetric scan, both standard absorption and difference (Δ*A*) optical spectra are shown in Fig. 5 for 2. For complex 3 a similar reversible redox behavior was observed in the region of the first reduction peak (Fig. S19 in the ESI†). The lowest reversibility of the electrochemical reduction was found for 1 (Fig. S20 in the ESI†). The metal-centered character of the reduction event was confirmed by EPR spectroscopy with complex 3. Chemical reduction with a stoichiometric equivalent of a single electron reducing agent, cobaltocene, produced an EPR signal characteristic for a low spin Co(ii) (d^7^, *S* = 1/2) in the complex labeled as 3′, shown in Fig. 6.^49–52^ The rhombic *g*-tensor with principal values (*g*_1_, *g*_2_, *g*_3_) of (2.2376, 2.1924, 2.0275) and resolved hyperfine coupling with the ^59^Co (*I* = 7/2), characterized by a rhombic *A*-tensor (*A*_1_, *A*_2_, *A*_3_) of (98, 153, 169 MHz), are in line with the distorted octahedral coordination polyhedron around Co(ii), presumably identical to that of the parent complex cation in 3.

The low-spin configuration of Fe(iii) in 4 and 5 was also confirmed by low-temperature EPR spectra (Fig. 6).

The principal values of the *g*-tensors (*g*_1_, *g*_2_, *g*_3_) for 4 (2.2157, 2.1241, 2.0026) and 5 (2.2213, 2.1229, 2.0027) in frozen MeCN/DMF glass are very similar and characteristic of the low-spin Fe(iii) state (d^5^, *S* = ½).^53^ As expected, the EPR spectra of 4 and 5 in DMSO at room temperature were broad and noisy but unambiguously confirmed their low-spin Fe(iii) ground state at 298 K as well.

**Fig. 7:**
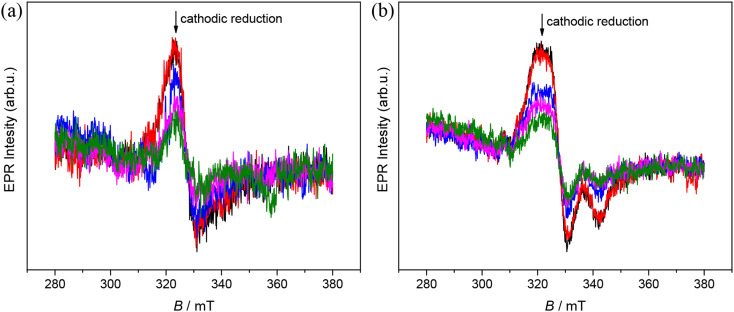
Time evolution of X-band EPR spectra of (a) 4 and (b) 5 in *n*Bu_4_NPF_6_/DMSO at room temperature in the region of the first cathodic peak when using the Pt mesh working electrode. Spectrometer settings: microwave frequency, 9.775 GHz; microwave power, 10 mW; modulation frequency, 100 kHz, modulation amplitude, 0.5 mT.

To provide further evidence that the reduction of complexes 4 and 5 is iron-centered, their reversible one-electron reduction was studied by *in situ* EPR spectroelectrochemistry. A clear decrease of the EPR signal was observed at the corresponding first cathodic peak for 4 and 5 in the *in situ* EPR spectroelectrochemical experiment directly in the EPR cavity using a large platinum working electrode and a flat spectroelectrochemical cell (Fig. 7), thus confirming the reduction of low spin Fe(iii) to the low spin EPR-inactive Fe(ii) species 4′ and 5′ (d^6^, *S* = 0).

**Fig. 8:**
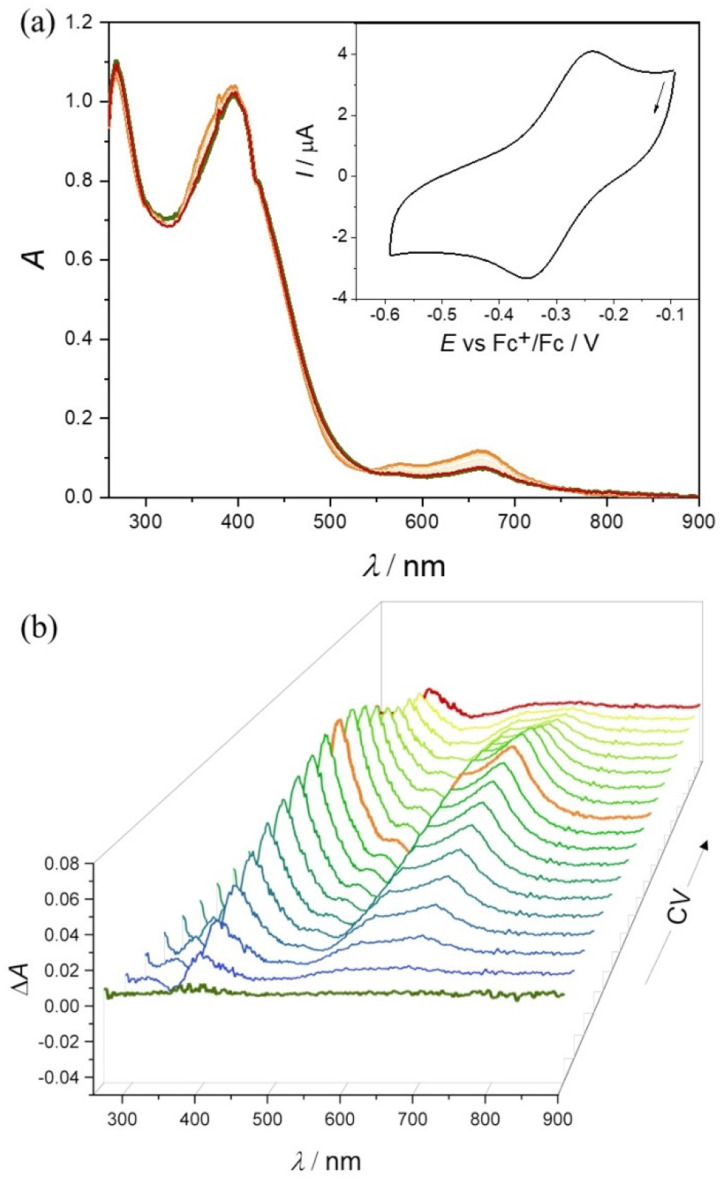
Spectroelectrochemistry of 4 in *n*Bu_4_NPF_6_/DMSO in the region of the first cathodic peak: (a) UV–vis–NIR spectra detected simultaneously upon *in situ* reduction; inset: CV of 4 with Pt-microstructured honeycomb working electrode and a scan rate of 10 mV s^−1^; (b) difference UV–vis–NIR spectra (the spectrum of the initial solution of 4 was taken as a reference) measured simultaneously with cyclic voltammetric scan (see the corresponding CV in (a)).

The formation of Fe(ii) analogues 4′ and 5′ upon cathodic reduction of 4 and 5 in DMSO, as well as the reversibility of the corresponding Fe(iii)/Fe(ii) redox couple, was also studied by *in situ* UV–vis–NIR spectroelectrochemistry. A new absorption band at 663 nm emerged after cathodic reduction of 4 in DMSO/*n*Bu_4_NPF_6_*via* an isosbestic point at 540 nm (Fig. 8). This result is reminiscent of those reported for other Fe(iii)–TSC complexes.^54–56^ Additionally, upon voltammetric reverse scan, reoxidation and nearly full recovery of the initial optical bands were observed, confirming the chemical reversibility of the cathodic reduction even at low scan rates. The reversible changes are even more evident in the difference optical spectra presented in a 3D projection (Fig. 8). A decrease of low intensity d–d transitions in the region 600–900 nm observed after the cathodic reduction of 4 provides further evidence for the metal centered electron transfer (Fig. S21 in the ESI†). A similar redox behavior was observed at a scan rate of 10 mV s^−1^ for 5 in the corresponding spectroelectrochemical experiment (Fig. S22 in the ESI†).

Given the stability of the Co(iii) and Fe(iii) complexes, the latter showing only slow and partial dissociation at pH 7.4, and the ability of the Fe(iii) species to be reduced both electrochemically and by GSH, which potentially makes them capable of producing reactive oxygen species (ROS), the antiproliferative activity of the complexes was further investigated.

### Cytotoxicity

The proligands HL^1^–HL^3^ and their corresponding metal complexes 1–8 prepared in this study were subjected to evaluation of their anticancer potential, including selectivity towards specific types of cancer: leukemia, non-small cell lung (NSCLC), colon, central nervous system (CNS), melanoma, ovarian, renal, prostate and breast cancers, by the National Cancer Institute's Developmental Therapeutics Program. This evaluation involved the utilization of the NCI 60 human tumor cell line panel.^57^ One-dose (10 μM) assays showed that HL^1^ and HL^2^, as well as complexes 3, 6–8 were devoid of antiproliferative activity in the low micromolar range (see Fig. S23–S28 in the ESI†). Five-dose (0.01 μM–100 μM) concentrations were applied for complexes 1, 2 and 4, 5 and the data were used to calculate 50% growth inhibition of tested cells (GI_50_), total growth inhibition (TGI), and lethal dose concentration inducing 50% cell death (LC_50_). The GI_50_ data in μM are collected in Table 5. 5-Dose screen curves for 1, 2, 4 and 5 are presented in Fig. S29–S32 in the ESI.†

**Table 5:** GI_50_ concentrations of 1, 2 and 4, 5 across the NCI 60 human cancer cell line panel accompanied by heat map data

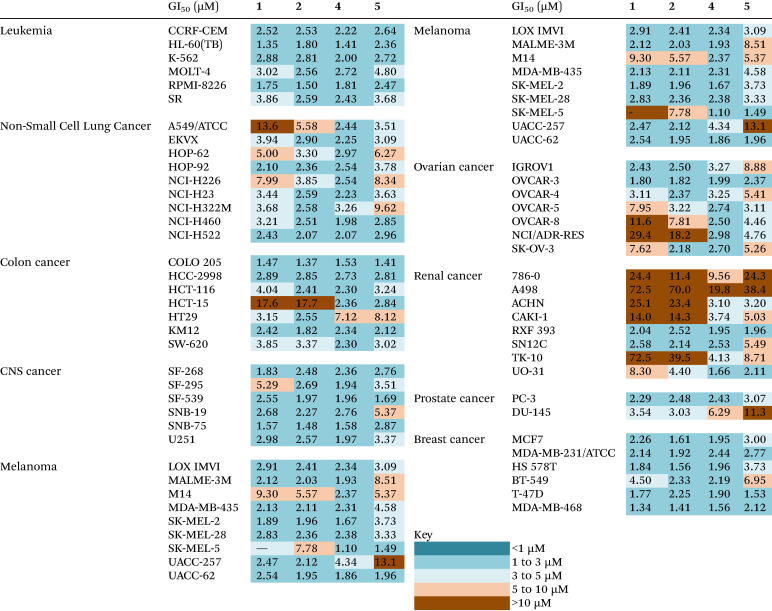

Notably, the iron(iii) complexes 4 and 5 displayed pronounced cytotoxic effect against the majority of cell lines within the panel. For the most active Fe(iii) complex 4, the average GI_50_, TGI, and LC_50_ concentrations across all cell lines in the panel were 2.92 μM, 18.4 μM, and 51.2 μM, respectively. For comparison, the Co(iii) complex 2 (with the same ligand as Fe(iii) complex 4) was somewhat less active with respective values of 5.63 μM, 39.9 μM and 68.3 μM, indicating that the two types of complexes may have distinct mechanisms of action.

Comparison of the GI_50_ values for different types of cancer cells indicates that the renal cancer cell lines are less sensitive to 1, 2 and 4, 5. In addition, the renal cancer cell lines show relative resistance to Co(iii) complexes 1 and 2, when compared to Fe(iii) complexes 4 and 5. Similarly, ovarian cancer cell lines (OVCAR-8, NCI/ADR-RES), colon cancer cells (HCT-15) and lung carcinoma cells (A549) showed relative resistance to 1 and 2, when compared to 4 and 5.

The robust growth inhibition and lethal concentration screening based on the NCI 60 human cancer cell line panel provided a basis to (i) evaluate the overall anticancer potential of the proligands and the most cytotoxic metal complexes, (ii) determine the selectivity of metal complexes towards specific types of cancers, (iii) select rationally the most potent compounds. The cellular and molecular effects of the selected complexes and the proligands were examined more extensively, and their effects on non-cancerous MRC-5 fibroblast cells were also determined.

**Fig. 9:**
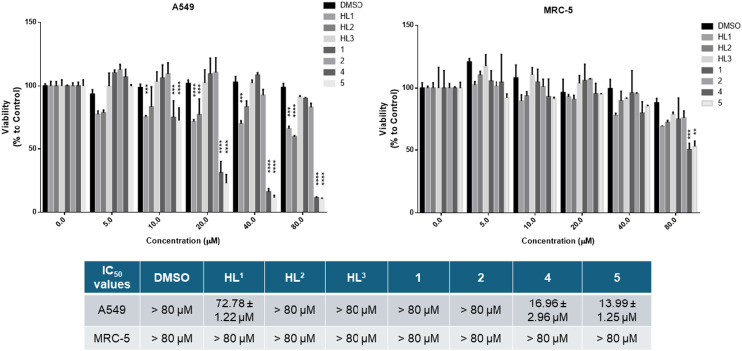
Viability of A549 tumor cells and MRC-5 fibroblast cells in the presence of HL^1^–HL^3^ and Co(iii) and Fe(iii) complexes 1, 2 and 4, 5, respectively, after a 24 h incubation determined by the MTT assay. *P* value: ** <0.005, *** <0.0005; **** <0.0001.

We wanted to compare the cytotoxicity of proligands HL^1^–HL^3^ and of their Co(iii) and Fe(iii) complexes 1, 2 and 4, 5 on cell lines originating from the same tissue, so human lung carcinoma A549 cells and lung fibroblast MRC-5 cells were subjected to 24 h treatments. Cell viability results and IC_50_ values (the μM drug concentration that reduces cell population to half the control value^58^) are shown in Fig. 9. The data indicate that HL^1^ and HL^2^ decrease somewhat the viability of the A549 lung adenocarcinoma cells at higher concentrations, but HL^3^ and the Co(iii) complexes lack significant anticancer activity with the A549 cells. These proligands HL^1^–HL^3^ and the Co(iii) complexes 1 and 2 did not affect the viability of MRC-5 fibroblasts. Importantly, Fe(iii) complexes 4 and 5 show significant toxicity towards A549 cancer cells, and the Fe(iii) complexes revealed good selectivity for the A549 cancer cell line (IC_50_ = 14–17 μM), as they were essentially non-toxic to non-cancerous MRC-5 cells (IC_50_ > 80 μM) except at the highest applied concentration. The vehicle DMSO exerted no toxic effect on either cell line.

**Fig. 10:**
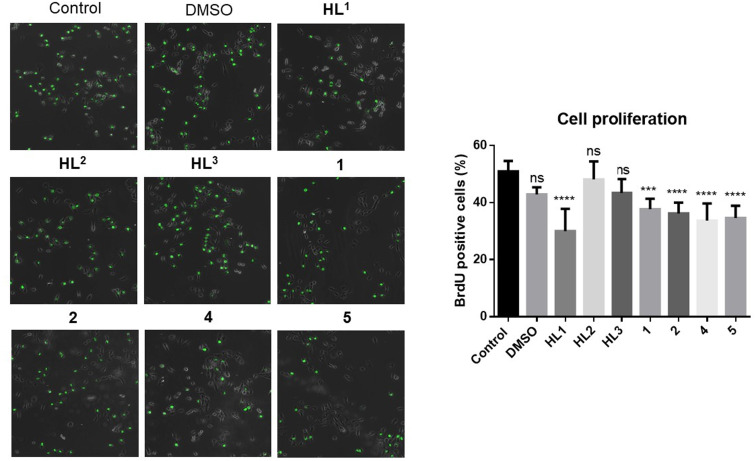
A549 cell proliferation in the presence of the proligands or their metal complexes assessed by the BrdU assay. The green fluorescent cells are BrdU positive, the non-fluorescent cells are BrdU negative.

To test directly the antiproliferative activity of the proligands and of their Co(iii) and Fe(iii) complexes on A549 cancer cells, non-toxic concentrations of the compounds were applied to the cells, then the 5-bromo-2′-deoxy-uridine (BrdU) incorporation assay was performed (Fig. 10). BrdU is a thymidine analog used in *in vitro* cell cultures to identify actively proliferating cells, since BrdU is incorporated into replicating DNA and can be detected using anti-BrdU antibodies. A reduction in the number of BrdU positive cells indicates that the applied treatment diminished cell proliferation. According to our results, DMSO vehicle control and the proligands HL^2^ and HL^3^ did not affect the proliferation of A549 cells compared to the untreated samples. On the other hand, significantly less BrdU incorporation was observed when cells were exposed to either HL^1^ ligand or to complexes 1, 2, 4 and 5. These results indicate a significant antiproliferative activity of the Co(iii) and Fe(iii) complexes, including the most active compounds 4 and 5.

### Interference with tubulin polymerization

Intrigued by the ability of recently reported Cu(ii) complex with HL^1^ to inhibit the polymerization of purified tubulin,^21^ we performed similar assays with complexes 1, 2, 4 and 6–8. As a reference for comparison, combretastatin A-4 (CA-4) and **Cu(HL**^**1**^**)Cl**_**2**_ were used. As shown in Table 6, among the 1 : 1 complexes only the square-planar Pd(ii) complex 8 showed appreciable inhibitory activity. The square-pyramidal Zn(ii) complex 7, structurally closely related to the recently reported Cu(ii) counterpart endowed with appreciable tubulin polymerization inhibitory activity (IC_50_ = 7.0 μM),^21^ did not show significant inhibitory activity even at 20 μM. Within the bis-ligand complexes, compound 4 showed the highest inhibitory activity so far, indicating that the metal complex stoichiometry and metal identity play an important role.

**Table 6:** Inhibition of tubulin polymerization and colchicine binding by 1, 2, 4 and 6–8^a^

Compound	IC_50_ ± SD (μM)	% inhibition ± SD	
		5 μM inhibitor	25 μM inhibitor
C-A4^b^	0.91 ± 0.1	97 ± 0.5	
4	3.4 ± 0.06	3.4 ± 5	17 ± 4
8	6.5 ± 1	13 ± 5	97 ± 1
**Cu(HL** ^**1**^ **)Cl**_**2**_ c	7.0 ± 0.3	22 ± 2	40 ± 3
1, 2, 6, 7	>20		

a Each experiment was performed 2–3 times, and SD's are presented.

b Combretastatin A-4.

c Taken from ref. 21.

Comparison of the GI_50_ values for cytotoxicity of 4 in cancer cells (Table 5) and inhibition of the polymerization of purified tubulin (3.4 μM) shows that these are mainly in the low micromolar range. This might indicate that the mode of action of 4 involves inhibition of tubulin assembly. In contrast, the one dose (10 μM) mean graph for 8 in cancer cells (Fig. S22 in the ESI†) indicated lower cytotoxicity of the compound, a reason why this compound was not examined in 5-dose assays to determine GI_50_ values. The IC_50_ value of inhibition of pure tubulin is 6.5 μM. The disjunction between antitubulin activity and cytotoxicity of 8 might be caused by multiple reasons.^59,60^ First, the drug concentrations quoted are almost always the concentrations in the culture medium. Neither the volume of the cells nor the proportion of drug that enters the cells or the rate of entry are generally known. Second, the drug can be altered in cells to become more active or less active. As for the Cu(ii) complex with HL^1^ ^21^ complex formation with Fe(iii) and Pd(ii) resulted in significant enhancement of the ability of the proligands to inhibit tubulin assembly. The active metal complexes 4 and 8 were further investigated for their abilities to inhibit the binding of [^3^H]colchicine to tubulin at two different concentrations (5 and 25 μM), with tubulin and colchicine at 0.5 and 5 μM concentrations, respectively (Table 6).^61,62^ The data obtained show that Pd(ii) complex 8 is by a factor of 5 superior to Fe(iii) complex 4 in its ability to inhibit the binding of [^3^H]colchicine to tubulin at 25 μM, but is still 7-fold less potent than CA-4 at the 5 μM concentration.

**Fig. 11:**
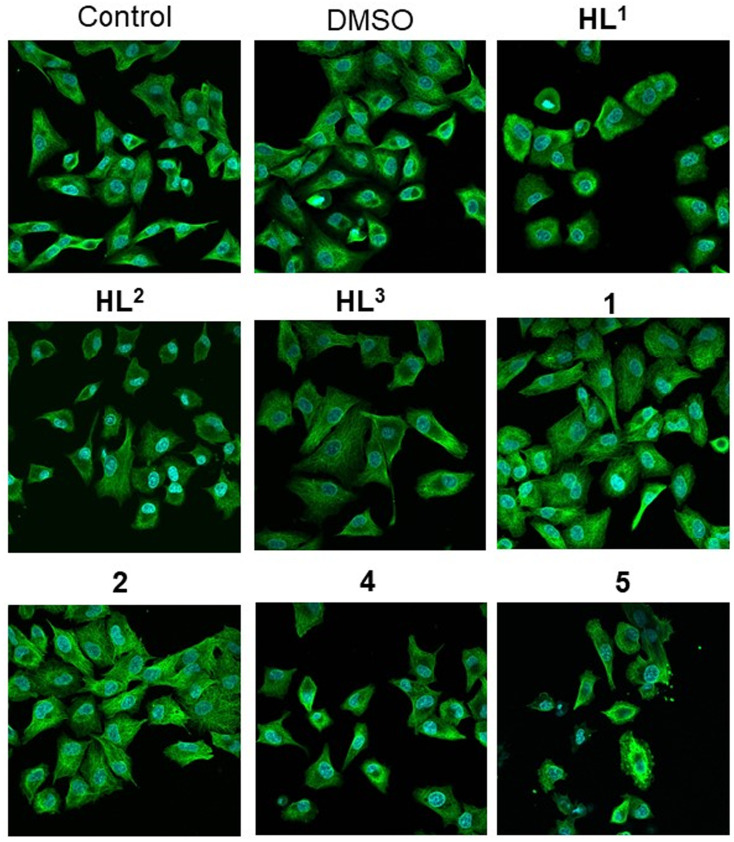
Fluorescent microscopic images of the microtubule system within A549 cancer cells after exposure to proligands HL^1^–HL^3^ or complexes 1, 2, 4 and 5 after tubulin immunostaining. Cell nuclei are visualized using DAPI.

Since we found remarkable antiproliferative activity exerted by some of the tested compounds by the BrdU assay, and it was also shown that some of the compounds might interfere with tubulin polymerization in cell-free tests, we examined whether the metal complexes or the proligands influence the structure of the microtubule system in A549 cancer cells. For this purpose, the cells were treated with either DMSO, HL^1^–HL^3^, or with complexes 1, 2, 4 and 5, or were left untreated, followed by α-tubulin immunostaining (Fig. 11). In the untreated samples normal cell morphology and filamentous microtubule structure was observed. The vehicle control DMSO, the ligands HL^2^ and HL^3^, and the metal complexes 1 and 2 did not affect either cell morphology or the filamentous structure of the microtubule system in the A549 cells. The microscopic images did show that microtubule structure was disrupted by the HL^1^ ligand treatment, and there was even greater disruption when the cells were exposed to 4 or 5 (Fig. 11). These microtubules do not span the entire cell in arrays but rather seem to be unstable and fuzzy.

Molecular docking was performed to predict the fit and orientation of 4 and 8 within the colchicine site of tubulin and thus validate the established antitubulin activity of these compounds.

### Molecular modelling

The Fe(iii) complex 4 and Pd(ii) complex 8 (Chart 1) were docked to the colchicine site of tubulin (PDB ID: 4O2B, resolution 2.30 Å);^63^ the docking scaffold was previously verified.^64^ The scoring functions GoldScore(GS),^65^ ChemScore(CS),^66,67^ Piecewise Linear Potential (ChemPLP)^68^ and Astex Statistical Potential (ASP)^69^ were used with the GOLD (v2024.1) docking algorithm. The GOLD docking algorithm is an excellent molecular modelling tool.^70,71^ Only GS runs were performed for the two metal complexes. The binding scores for the complexes are shown in Table 7. The complexes show good scores, indicating reasonable binding similar that of the *N*-[(7*S*)-1,2,3,10-tetramethoxy-9-oxo-6,7-dihydro-5*H*-benzo[*d*]heptalen-7-yl]ethanamide (LOC) co-crystalized ligand. The higher binding score for complex 8 (Table 7) is in line with its superior ability to inhibit the binding of [^3^H]colchicine to tubulin at 25 μM as compared with complex 4 (see Table 6).

**Table 7:** The binding affinities of 4, 8 and *N*-[(7*S*)-1,2,3,10-tetramethoxy-9-oxo-6,7-dihydro-5*H*-benzo[*d*]heptalen-7-yl]ethanamide (LOC) as predicted by the scoring functions for the colchicine site of tubulin. LOC was re-docked and reproduced the crystal structure well with low Root Mean Square Distance (RMSD)

Complexes	GS	ASP	PLP	CS	IC_50_ (μM)
4	62.9	—	—	—	3.4 ± 0.06
8	78.0	—	—	—	6.5 ± 1
LOC	72.6	29.1	73.3	29.0	—
RMSD (Å)	0.3251	0.3659	0.2410	0.4822	—

**Fig. 12:**
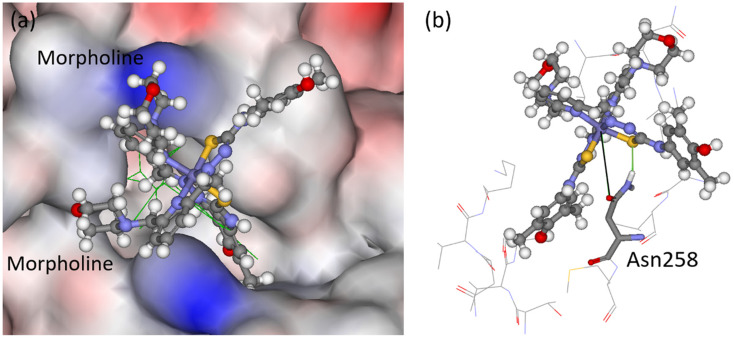
(a) The docked pose of 4 (ball-and-stick) in the colchicine site of tubulin. The co-crystalized ligand (LOC) is shown in line format (green), and its hydrogen atoms are not shown for clarity. The protein surface is rendered with blue color depicting regions with a partial positive charge on the surface, red color depicting regions with a partial negative charge and grey showing neutral areas. (b) The predicted binding of complex 4, with amino acids within 5 Å shown in line format. H-bonding is predicted between the βAsn258 (stick format) side chain and one of the sulfur atoms in the complex (green solid line, 2.1 Å), and there is a potential interaction of the amide side chain oxygen atom with the central iron (black solid line, 4.5 Å).

The modelling into the tubulin-colchicine pocket revealed that both complexes overlap extensively with the LOC co-crystalized ligand (see Fig. 12 and Fig. S33 in the ESI†). Fig. 12 shows the predicted binding mode of complex 4. It fits into the pocket with both morpholine rings pointing into the water environment. One H-bonding interaction is predicted between the amino group in the side chain of Asn258 and the thiolato sulfur atom of one of the TSC ligands. In addition, the oxygen atom in the amide side chain came close to the iron(iii) center.

## Conclusions

This work led to a series of cobalt(iii) and iron(iii) complexes with three closely related TSCs HL^1^–HL^3^ (except that of Fe(iii) with HL^1^) of 1 : 2 stoichiometry and nickel(ii), zinc(ii) and palladium(ii) complexes with HL^1^ of 1 : 1 stoichiometry. The three proligands resulted from condensation reactions of 6-morpholinomethyl-2-formylpyridine and 6-morpholinomethyl-2-acetylpyridine with 4-(4-hydroxy-3,5-dimethylphenyl)thiosemicarbazide (HL^1^ and HL^2^, respectively) or 6-morpholinomethyl-2-formylpyridine and 4-*N*-phenylthiosemicarbazide (HL^3^). X-ray diffraction studies have shown that TSC-morpholine hybrids act as tridentate monoanionic or zwitterionic ligands in cobalt(iii) and iron(iii) complexes of 1 : 2 stoichiometry, namely [Co(HL^1^)(L^1^)](NO_3_)_2_ (1), [Co(HL^2^)(L^2^)](NO_3_)_2_ (2), [Co(HL^3^)(L^3^)](NO_3_)_2_ (3), [Fe(L^2^)_2_]NO_3_ (4) and [Fe(HL^3^)(L^3^)](NO_3_)_2_ (5). In contrast, in 1 : 1 metal-to-ligand complexes the coordination mode and protonation state of HL^1^ differ. In square-planar nickel(ii) and square-pyramidal zinc(ii) complexes [Ni(L^1^)]Cl and [Zn(L^1^)Cl] the TSC-morpholine hybrid acts as monoanionic tetradentate ligand, while in square-planar palladium(ii) complex [Pd(HL^1^)Cl]Cl HL^1^ adopts a zwitterionic form being protonated at morpholine nitrogen atom and deprotonated at the thiosemicarbazide fragment, with the negative charge formally localized on the thiolato sulfur atom and acting as a tridentate ligand. The bis-ligand metal complexes (1, 2 and 4, 5) showed good cytotoxicity in a panel of 60 cancer cell lines. In contrast, the *mono*-ligand complexes 6–8 were devoid of cytotoxicity in the low μM range. Antiproliferative activity assays showed good selectivity (selectivity index *ca.* 5) of Fe(iii) complexes 4 and 5 for human lung carcinoma A549 cells when compared to lung fibroblast MRC-5 cells. In addition, the complexes 4 and 8 were found to show good antitubulin activity with IC_50_ value of 3.4 and 6.5 μM, respectively. Comparison of the average GI_50_ concentration of Fe(iii) complex 4 against the NCI 60 tumor cell panel (2.92 μM) with the IC_50_ value for inhibition of tubulin assembly (3.4 μM) leads to the conclusion that tubulin might be a target for this compound. This is also consistent with the tubulin immunostaining experiment in A549 cells and with molecular docking calculations. The complexes 4 and 8 are the first reported Fe(iii) and Pd(ii) complexes acting as inhibitors of tubulin assembly. The change of metal-to-ligand stoichiometry and metal identity seem to be important for further structural optimization of metal complexes in order to obtain compounds with improved antitubulin activity.

## Experimental section

### Chemicals

2-Formylpyridine, 2-acetylpyridine, 4-*N*-phenyl-3-thiosemicarbazide, Co(NO_3_)_2_·6H_2_O, Fe(NO_3_)_3_·9H_2_O, NiCl_2_·6H_2_O, ZnCl_2_, PdCl_2_(MeCN)_2_ were purchased from commercial suppliers and used without further purification. The syntheses of 6-(morpholinomethyl)-2-formylpyridine, 6-(morpholinomethyl)-2-acetylpyridine and 4-*N*-(4-hydroxy-3,5-dimethylphenyl)-3-thiosemicarbazide were performed by following literature protocols.^24–26^

### The synthesis of the proligands

#### General method

A solution of the corresponding aldehyde or ketone (2 mmol) and 4*N*-(4-hydroxy-3,5-dimethylphenyl)-3-thiosemicarbazide (2 mmol) in ethanol (40 mL) was refluxed for 2 h (or overnight in the case of HL^2^**·0.5H_2_O**). The clear yellow solution was concentrated under reduced pressure to *ca.* 2 mL and left to stand at 4 °C for 6 h. The pale-yellow crystalline product was filtered, washed with cold ethanol, and dried *in vacuo*.

#### 6-(Morpholinomethyl)-2-formylpyridine 4*N*-(4-hydroxy-3,5-dimethylphenyl)-3-thiosemicarbazone (HL^1^·0.75H_2_O)

Yield: 774 mg, 94% Anal. Calcd for C_20_H_25_N_5_SO_2_·0.75H_2_O (*M*_r_ = 413.02): C, 58.16; H, 6.47; N, 16.96; S, 7.76%. Found, %: C, 58.22; H, 6.12; N, 16.93; S, 7.79. Positive ion ESI-MS (MeCN/MeOH + 1% H_2_O): *m*/*z* 400.18 [HL^1^ + H]^+^, 422.16 [HL^1^ + Na]^+^. ^1^H NMR (600 MHz, DMSO-*d*_6_, *E*-isomer): *δ* 11.86 (s, 1H, H_9_), 9.98 (s, 1H, H_11_), 8.32 (d, *J* = 7.9 Hz, 1H, H_3_), 8.21 (s, 1H, H_18_), 8.11 (s, 1H, H_7_), 7.80 (t, *J* = 7.7 Hz, 1H, H_4_), 7.42 (d, *J* = 7.5 Hz, 1H, H_5_), 7.01 (s, 2H, H_13+17_), 3.59 (s, 6H, H_21+24+25_), 2.42 (s, 4H, H_23+26_), 2.17 (s, 6H, H_19+20_) ppm. ^13^C NMR (151 MHz, DMSO-*d*_6_, *E*-isomer): *δ* 176.98 (C_10_), 158.16 (C_6_), 153.23(C_2_), 151.57 (C_15_), 143.00 (C_7_), 137.33 (C_4_), 130.65 (C_12_), 126.75 (C_13+17_), 124.30 (C_14+16_), 123.63 (C_5_), 119.40 (C_3_), 66.65 (C_24+25_), 64.39 (C_21_), 53.79 (C_23+26_), 17.10 (C_19+20_) ppm. In DMSO *E*/*Z* isomers are present in a 40 : 1 molar ratio.

#### 6-(Morpholinomethyl)-2-acetylpyridine 4*N*-(4-hydroxy-3,5-dimethylphenyl)-3-thiosemicarbazone (HL^2^·0.5H_2_O)

Yield: 618 mg, 73%. M.p. 208–209 °C. Anal. Calcd for C_21_H_27_N_5_SO_2_·0.5H_2_O (*M*_r_ = 422.55): C, 59.69; H, 6.68; N, 16.57; S, 7.59%. Found, %: C, 59.44; H, 6.41; N, 16.36; S, 7.35. Positive ion ESI-MS (MeCN/MeOH + 1% H_2_O): *m*/*z* 414.20 [HL^2^ + H]^+^, 436.18 [HL^2^ + Na]^+^. Negative ion ESI-MS (MeCN/MeOH +1% H_2_O): *m*/*z* 412.16 [HL^2^ − H]^−^. ^1^H NMR (500 MHz, DMSO-*d*_6_, *E*-isomer): *δ* 10.43 (s, 1H, H_9_), 9.93 (s, 1H, H_11_), 8.42 (d, *J* = 7.8 Hz, 1H, H_3_), 8.23 (s, 1H, H_18_), 7.77 (t, *J* = 7.8 Hz, 1H, H_4_), 7.44 (d, *J* = 7.6 Hz, 1H, H_5_), 7.01 (s, 2H, H_13+17_), 3.64 (s, 2H, H_21_), 3.60 (s, 4H, H_24+25_), 2.45 (s, 3H, H_7′_), 2.43 (s, 4H, H_23+26_), 2.17 (s, 6H, H_19+20_) ppm. ^13^C NMR (151 MHz, DMSO-*d*_6_, *E*-isomer): *δ* 177.28 (C_10_), 156.90 (C_6_), 153.92 (C_2_), 151.07 (C_15_), 138.86 (C_7_), 136.74 (C_4_), 130.33 (C_12_), 126.27 (C_13+17_), 123.79 (C_14+15_), 122.82 (C_5_), 119.50 (C_3_) 66.20 (C_21_), 64.13 (C_24+25_), 53.24 (C_23+26_), 16.60 (C_19+20_), 12.26 (C_7′_) ppm. In DMSO *E*/*Z* isomers are present in a 30 : 1 molar ratio. IR (ATR, selected bands, *υ̃*_max_): 3316, 3182, 1612, 1576, 1451, 1189, 1114, 867, 593, 337 cm^−1^. UV–vis (MeOH), *λ*_max_, nm (*ε*, M^−1^ cm^−1^): 317 (38 853). Other details can be found in a recently published article.^21^

#### 6-(Morpholinomethyl)-2-formylpyridine 4*N*-phenyl-3-thiosemicarbazone (HL^3^)

Yield: 620 mg, 87% M.p. 111–112 °C. Anal. Calcd for C_18_H_21_N_5_SO (*M*_r_ = 355.47): C, 60.82; H, 5.95; N, 19.70; S, 9.02%. Found, %: C, 61.09; H, 5.74; N, 19.80; S, 8.92. Positive ion ESI-MS (MeCN/MeOH + 1% H_2_O): *m*/*z* 356.17 [HL^3^ + H]^+^, 378.16 [HL^3^ + Na]^+^. Negative ion ESI-MS (MeCN/MeOH +1% H_2_O): *m*/*z* 354.11 [HL^3^ − H]^−^. ^1^H NMR (500 MHz, DMSO-*d*_6_, *E*-isomer): *δ* 12.04 (s, 1H, H_9_), 10.24 (s, 1H, H_11_), 8.34 (d, *J* = 7.9 Hz, 1H, H^3^), 8.15 (s, 1H, H_7_), 7.82 (t, *J* = 7.8 Hz, 1H, H_4_), 7.54 (d, *J* = 7.4 Hz, 2H, H_5_), 7.44 (d, *J* = 7.4 Hz, 1H, H_13+17_), 7.39 (t, *J* = 7.9 Hz, 2H, H_14+16_), 7.23 (t, *J* = 7.4 Hz, 1H, H_15_), 3.59 (d, *J* = 6.9 Hz, 6H, H_18+21+22_), 2.42 (s, 4H, H_20+23_) ppm. ^13^C NMR (151 MHz, DMSO-*d*_6_, *E*-isomer): *δ* 176.99 (C_10_), 153.76 (C_6_), 149.17(C_2_), 142.47 (C_12_), 139.28(C_7_), 138.10 (C_4_), 128.61 (C_14+16_), 126.65 (C_13+17_), 126.10 (C_15_), 125.57 (C_5_), 121.19 (C_3_), 63.59 (C_21+22_), 60.23 (C_18_), 51.86 (C_20+23_) ppm. In DMSO *E*/*Z* isomers are present in a 20 : 1 molar ratio. IR (ATR, selected bands, *υ̃*_max_): 3211, 2440, 1730, 1591, 1517, 1250, 1189, 694 cm^−1^. UV–vis (MeOH), *λ*_max_, nm (*ε*, M^−1^ cm^−1^): 277 sh, 327 (18 220).

### Oxidized ligands

#### General method

To a solution of Fe(NO_3_)_3_·9H_2_O (0.2 mmol) in ethanol (5 mL) a solution of the proligand (0.1 mmol) in ethanol (5 mL) was added dropwise, and the mixture was stirred at 50 °C for 10 min.

#### *N*-(4-Hydroxy-3,5-dimethylphenyl)-6-(morpholinomethyl-2-formylpyridine)-1,3,4-thiadiazol-2-amine (H*L*^1′^·H_2_O)

The reaction mixture was evaporated to dryness, and the residue was dissolved in DMF. Yellow crystals, obtained by slow diffusion of diethyl ether into a solution of the product in DMF, were removed by filtration, washed with cold methanol (2 × 1 mL) and dried *in vacuo*. Yield: 20 mg, 43%. Anal. Calcd for C_20_H_23_N_5_SO_2_·H_2_O (*M*_r_ = 415.51): C, 57.81; H, 6.06; N, 16.85; S, 7.72%. Found, %: C, 57.59; H, 6.22; N, 16.71; S, 7.89. Positive ion ESI-MS (MeCN/MeOH + 1% H_2_O): *m*/*z* 398.17 [H*L*^1′^ + H]^+^. ^1^H NMR (700 MHz, DMSO-*d*_6_): *δ* 10.26 (s, 1H, H_11_), 10.14 (s, 1H, H_22_), 8.16 (d, *J* = 7.0 Hz, 1H, H_3_), 8.12 (s, 1H, H_18_), 8.08 (d, *J* = 7.2 Hz, 1H, H_4_), 7.58 (d, *J* = 7.5 Hz, 1H, H_5_), 7.16 (s, 2H, H_13+17_), 4.59 (s, 2H, H_21_), 4.00 (s, 2H, H_24/25_), 3.72 (s, 2H, H_24/25_), 3.50 (s, 2H, H_23/26_), 3.29 (s, 2H, H_23/26_), 2.18 (s, 6H, H_19+20_) ppm. ^13^C NMR (176 MHz, DMSO-*d*_6_): *δ* 167.84 (C_10_), 157.91 (C_7_), 150.77 (C_15_), 149.70 (C_6_), 149.67(C_2_), 139.38 (C_4_), 132.89 (C_12_), 125.79 (C_5_), 125.61 (C_14+16_), 119.89 (C_3_) 119.48 (C_13+17_), 63.77 (C_24+25_), 59.42 (C_21_), 52.35 (C_23+26_), 17.33 (C_19+20_) ppm.

#### *N*-Phenyl-6-(morpholinomethyl-2-formylpyridine)-1,3,4-thiadiazol-2-amine (H*L*^3′^)

Upon standing, a methanol solution of HL^3^ with Fe(NO_3_)_3_·9H_2_O in a 2 : 1 ligand-to-metal ratio, a small amount of oxidized proligand was formed. This was confirmed by positive ion ESI-MS (MeCN/MeOH + 1% H_2_O) (*m*/*z* 354.17 [H*L*^3′^ + H]^+^) and negative ion ESI-MS (*m*/*z* 351.99 [H*L*^3′^ − H]^−^). Anal. Calcd for C_18_H_19_N_5_SO (*M*_r_ = 353.44): C, 61.17; H, 5.42; N, 19.82; S, 9.07%. Found, %: C, 61.26; H, 5.56; N, 19.69; S, 8.89. ^1^H NMR (600 MHz, DMSO-*d*_6_): *δ* 10.65 (s, 1H, H_11_), 8.02 (d, *J* = 7.7 Hz, 1H, H_3_), 7.95 (d, *J* = 7.7 Hz, 1H, H_4_), 7.67 (d, *J* = 7.8 Hz, 2H, H_13+17_), 7.53 (d, *J* = 7.6 Hz, 1H, H_5_), 7.38 (t, *J* = 7.9 Hz, 2H, H_14+16_), 7.04 (s, 1H, H_15_), 3.66 (s, 2H, H_18_), 3.61 (m, 4H, H_21+22_), 2.47 (s, 4H, H_20+23_) ppm. ^13^C NMR (151 MHz, DMSO-*d*_6_): *δ* 165.57 (C_10_), 159.67 (C_7_), 158.70 (C_6_), 148.41 (C_2_), 140.43 (C_12_), 137.94 (C_4_), 129.13 (C_14+16_), 123.64 (C_5_), 122.18 (C_15_), 117.75 (C_3_), 117.65 (C_13+17_), 66.22 (C_21+22_), 63.53 (C_18_), 53.24 (C_20+23_) ppm.

### Synthesis of complexes with metal-to-ligand stoichiometry of 1 : 2

#### General procedure

To a solution of the corresponding proligand (0.1 mmol) in methanol (5 mL) at 50 °C was added dropwise a solution of Co(NO_3_)_2_·6H_2_O or Fe(NO_3_)_3_·9H_2_O (0.05 mmol) in methanol (5 mL). The resulting mixture was stirred at 50 °C for 10 min. Upon cooling to room temperature, dark-red crystals were formed. The crystals were separated by filtration, washed with cold methanol (2 × 1 mL) and dried *in vacuo*.

#### [Co^III^(HL^1^)(L^1^)](NO_3_)_2_·2H_2_O (1)

Yield: 40 mg, 79%. Anal. Calcd for C_40_H_49_CoN_12_O_10_S_2_·2H_2_O (*M*_r_ = 1016.99): C, 47.24; H, 5.25; N, 16.53; S, 6.31%. Found, %: C, 47.15; H, 4.95; N, 16.30; S, 6.04. Positive ion ESI-MS in MeCN/MeOH + 1% H_2_O: *m*/*z* 855.33 [Co^III^(L^1^)_2_]^+^. ^1^H NMR (700 MHz, DMSO-*d*_6_): *δ* 10.20 (brs, 1H, H_11_), 9.02 (s, 1H, H_7_), 8.56–8.19 (brs, 1H, H_18_), 8.17 (t, *J* = 7.6 Hz, 1H, H_4_), 8.01 (d, *J* = 7.5 Hz, 1H, H_3_), 7.64 (d, *J* = 6.0 Hz, 1H, H_5_), 7.04 (brs, 2H, H_13+17_), 3.49 (s, 2H, H_24/25_), 3.41 (s, 2H, H_24/25_), 2.37 (s, 2H, H_23/26_), 2.19 (s, 2H, H_23/26_), 2.13 (s, 6H, H_19+20_) ppm. The resonance for H_21_ was overlapped with the peak of the residual water. The H_22_ proton of the morpholinium moiety has not been seen in ^1^H NMR spectrum. ^13^C NMR (176 MHz, DMSO-*d*_6_): *δ* 158.70 (C_2_), 151.04 (C_15_), 140.74 (C_4_), 127.31 (C_5_), 126.51 (C_3_), 125.09 (C_16+14_), 65.62 (C_24+25_), 60.06 (C_21_), 53.29 (C_23+26_), 17.28 (C_19+20_) ppm. The number of resonances observed indicates a 2-fold symmetry of the complex cation in solution. IR (ATR, selected bands, *υ̃*_max_): 3416, 1627, 1297, 1110, 1016, 865, 785 cm^−1^. UV–vis (MeOH), *λ*_max_, nm (*ε*, M^−1^ cm^−1^): 266 (70 965), 303 sh, 418 (41 503), 482sh, 535sh, 580sh.

#### [Co^III^(HL^2^)(L^2^)](NO_3_)_2_·3H_2_O (2)

Yield: 41 mg, 77%. Anal. Calcd for C_42_H_53_CoN_12_O_10_S_2_·3H_2_O (*M*_r_ = 1062.05): C, 47.45; H, 5.59; N, 15.81; S, 6.03%. Found, %: C, 47.38; H, 5.49; N, 15.84; S, 6.16. Positive ion ESI-MS in MeCN/MeOH + 1% H_2_O: *m*/*z* 883.34 [Co^III^(L^2^)_2_]^+^. ^1^H NMR (600 MHz, DMSO-*d*_6_): *δ* 10.10 (s, 1H, H_11_), 8.23 (s, 1H, H_19_), 8.16 (t, *J* = 7.8 Hz, 1H, H_4_), 8.06 (d, *J* = 7.7 Hz, 1H, H_5_), 7.69 (d, *J* = 7.8 Hz, 1H, H_3_), 7.12 (s, 2H, H_13+17_), 3.52 (brs, 4H, H_23/26+21_; overlapped by the signal of water), 3.37–3.21 (brs, 2H, H_23/26_), 2.86 (s, 3H, H_7′_), 2.19 (brs, 2H, H_25/24_), 2.15 (s, 6H, H_19+20_), 2.12–2.09 (brs, 2H, H_25/24_) ppm. The proton H_22_ of the morpholinium moiety was not observed in ^1^H NMR spectrum. ^13^C NMR (151 MHz, DMSO-*d*_6_): *δ* 163.25 (C_6_), 159.69 (C_2_), 157.76 (C_7_), 150.04 (C_15_), 140.37 (C_4_), 125.97 (C_5_), 124.59 (C_3_), 124.51 (C_14+16_), 120.94 (C_12_), 65.69 (C_21_), 59.61 (C_23+26_), 53.29 (C_24+25_), 16.83 (C_19+20_), 16.24 (C_7′_) ppm. Three carbon atoms C_13_, C_17_ and C_10_ were not seen in ^13^C NMR spectrum. The number of resonances observed indicates a 2-fold symmetry of the complex cation in solution. IR (ATR, selected bands, *υ̃*_max_): 3260, 3080, 1488, 1455, 1409, 1299, 1207, 1146, 1009, 860, 493 cm^−1^. UV–vis (MeOH), *λ*_max_, nm (*ε*, M^−1^ cm^−1^): 261 (62 438), 301sh, 417 (35 270), 482sh, 535sh, 580sh.

#### [Co^III^(HL^3^)(L^3^)](NO_3_)_2_·H_2_O (3)

Yield: 40 mg, 88%. Anal. Calcd for C_36_H_41_CoN_12_O_8_S_2_·H_2_O (*M*_r_ = 910.87): C, 47.47; H, 4.76; N, 18.45; S, 7.04%. Found, %: C, 47.14; H, 4.55; N, 18.24; S, 7.01. Positive ion ESI-MS in MeCN/MeOH + 1% H_2_O: *m*/*z* 767.29 [Co^III^(L^3^)_2_]^+^. ^1^H NMR (600 MHz, DMSO-*d*_6_): *δ* 10.54 (s, 1H, H_11_), 9.18 (s, 1H, H_7_), 8.20 (t, *J* = 7.7 Hz, 1H, H_4_), 8.05 (t, *J* = 7.3 Hz, 1H, H_3_), 7.63 (d, *J* = 7.7 Hz, 1H, H_5_), 7.57 (dd, *J* = 22.1, 7.6 Hz, 2H, H_13+17_), 7.36 (q, *J* = 7.4 Hz, 2H, H_16+14_), 7.11 (t, *J* = 7.4 Hz, 1H, H_15_), 3.59 (s, 2H, H_18_), 3.43 (s, 2H, H_21/22_), 3.32 (s, 2H, H_21/22_), 2.29 (s, 2H, H_20/23_), 2.08 (s, 2H, H_20/23_) ppm. The proton H_19_ of the morpholinium moiety was not seen in ^1^H NMR spectrum. ^13^C NMR (151 MHz, DMSO-*d*_6_): *δ* 158.21 (C_6_), 155.30 (C_2_), 140.22 (C_4_), 139.38 (C_12_), 128.90 (C_16+14_), 127.67 (C_5_), 126.31 (C_3_), 123.93 (C_15_), 120.58 (C_13+17_), 65.44 (C_21+22_), 60.29 (C_18_), 52.89 (C_20+23_) ppm. The C_7_ and C_10_ carbon atoms were not seen in ^13^C NMR spectrum. IR (ATR, selected bands, *υ̃*_max_): 3308, 1601, 1551, 1481, 1252, 1108, 746, 686, 557, 488 cm^−1^. UV–vis (MeOH), *λ*_max_, nm (*ε*, M^−1^ cm^−1^): 257 (62 509), 307 sh, 396 (34 215), 482sh, 535sh, 580sh.

#### [Fe^III^(L^2^)_2_]NO_3_·0.5H_2_O (4)

Yield: 40 mg, 80%. Anal. Calcd for C_42_H_52_FeN_11_O_7_S_2_·3H_2_O (*M*_r_ = 996.96): C, 50.60; H, 5.86; N, 15.45; S, 6.43%. Found, %: C, 50.87; H, 5.43; N, 15.39; S, 6.87. Positive ion ESI-MS of 4 in MeCN/MeOH + 1% H_2_O: *m*/*z* 880.37 [Fe^III^(L^2^)_2_]^+^. IR (ATR, selected bands, *υ̃*_max_): 3239, 1601, 1574, 1480, 1407, 1252, 1021, 825, 688, 490 cm^−1^. UV–vis (MeOH), *λ*_max_, nm (*ε*, M^−1^ cm^−1^): 260 (30 000), 403 (18 229), 449sh, 580 (800), 710 (570), 800 (560).

#### [Fe^III^(HL_3_)(L_3_)](NO_3_)_2_·CH_3_OH·H_2_O (5)

Yield: 72 mg, 89%. Anal. Calcd for C_36_H_41_FeN_12_O_8_S_2_·CH_3_OH·H_2_O (*M*_r_ = 939.82): C, 47.29; H, 5.04; N, 17.88; S, 6.82%. Found, %: C, 47.64; H, 4.93; N, 17.77; S, 6.79. Positive ion ESI-MS for 5 in MeCN/MeOH + 1% H_2_O: *m*/*z* 764.23 [Fe^III^(L^3^)_2_]^+^. IR (ATR, selected bands, *υ̃*_max_): 3253, 1600, 1547, 1479, 1442, 1318, 1109, 1021, 743, 641, 496 cm^−1^. UV–vis (MeOH), *λ*_max_, nm (*ε*, M^−1^ cm^−1^): 257 (35 179), 399 (23 980), 444 sh, 575sh, 685 (400), 805 (300).

### Synthesis of complexes with metal-to-ligand stoichiometry of 1 : 1

#### General procedure

To a solution of the proligand (0.2 mmol) in methanol (8 mL) at 50 °C was added dropwise a solution of NiCl_2_·6H_2_O, ZnCl_2_ or PdCl_2_(MeCN)_2_ (0.2 mmol) in methanol (2 mL). The resulting mixture was stirred at 50 °C for 10 min. After allowing the solution to cool to room temperature and slow evaporation of methanol, crystals were formed, which were separated by filtration, washed with cold methanol (2 × 1 mL) and dried *in vacuo*.

#### [Ni^II^(L^1^)]Cl·2.5H_2_O (6)

Yield: 62 mg, 58%. Anal. Calcd for C_20_H_25_ClNiN_5_O_2_S·2.5H_2_O (*M*_r_ = 538.70): C, 44.59; H, 5.61; N, 13.00; S, 5.95%. Found, %: C, 44.62; H, 5.25; N, 12.64; S, 5.87. Positive ion ESI-MS for 6 in MeCN/MeOH + 1% H_2_O (positive): *m*/*z* 456.15 [Ni^II^(L^1^)]^+^. IR (ATR, selected bands, *υ̃*_max_): 3199, 1604, 1459, 1414, 1208, 1125, 857, 521, 492 cm^−1^. UV–vis (MeOH), *λ*_max_, nm (*ε*, M^−1^ cm^−1^): 261 (14 702), 314 sh, 430 (82 375), 489sh, 620sh. X-ray diffraction quality single crystals were selected from the prepared sample.

#### Zn^II^(L^1^)Cl·1.5H_2_O (7)

Yield: 37 mg, 74%. Anal. Calcd for C_20_H_24_ClN_5_O_2_SZn·1.5H_2_O (*M*_r_ = 526.37): C, 45.64; H, 5.17; N, 13.31; S, 6.09%. Found, %: C, 45.87; H, 4.77; N, 13.12; S, 5.82. Positive ion ESI-MS for 7 in MeCN/MeOH + 1% H_2_O (positive): *m*/*z* 426.09 [Zn^II^(L^1^)]^+^. ^1^H NMR (600 MHz, DMSO-*d*_6_): *δ* 9.52 (s, 1H, H_11_), 8.36 (s, 1H, H_7_), 8.13 (t, *J* = 7.7 Hz, 1H, H_4_), 8.01 (s, 1H, H_18_), 7.66 (d, *J* = 7.7 Hz, 1H, H_3_), 7.54 (d, *J* = 7.8 Hz, 1H, H_5_), 7.23 (s, 2H, H_13+17_), 3.91 (s, 2H, H_21_), 3.81 (s, 4H, H_24+25_), 2.77 (s, 4H, H_23+26_), 2.15 (s, 6H, H_19+20_) ppm. ^13^C NMR (151 MHz, DMSO-*d*_6_): *δ* 153.63 (C_6_), 149.16 (C_15_), 148.09 (C_2_), 141.86 (C_4_), 137.28 (C_7_), 131.94 (C_12_), 124.01 (C_14+16_), 123.13 (C_5_), 122.53 (C_3_), 122.08 (C_13+17_), 64.91 (C_25+24_), 60.77 (C_21_), 53.38 (C_23+26_), 16.84 (C_19+20_) ppm. Single crystals of X-ray diffraction quality were obtained by slow diffusion of diethyl ether into methanolic solution of 7. IR (ATR, selected bands, *υ̃*_max_): 3465, 3276, 1607, 1560, 1490, 1466, 1440, 1224, 1191, 1052, 842, 501 cm^−1^. UV–vis (MeOH), *λ*_max_, nm (*ε*, M^−1^ cm^−1^): 272 (10 527), 409 (13 822).

#### [Pd^II^(HL^1^)Cl]Cl·H_2_O (8)

Acetonitrile was used as solvent. The reaction mixture was heated at 80 °C for 1 h. The precipitate formed was isolated by filtration after cooling the mixture to room temperature. It was washed with MeCN (1 mL) and dried in air. Yield: 110 mg, 92%. Anal. Calcd for C_20_H_25_Cl_2_N_5_O_2_PdS·H_2_O (*M*_r_ = 594.95): C, 40.38; H, 4.57; N, 11.77; S, 5.39%. Found, %: C, 40.29; H, 4.27; N, 12.07; S, 5.04. Positive ion ESI-MS for 8 in MeCN/MeOH + 1% H_2_O (positive): *m*/*z* 504.07 [Pd^II^(L^1^)]^+^. ^1^H NMR (600 MHz, DMSO-*d*_6_ + 10% MeOH-*d*_4_): *δ* 8.26 (t, *J* = 7.6 Hz, 1H, H_4_), 7.91 (d, *J* = 7.5 Hz, 1H, H_3_), 7.87 (d, *J* = 7.5 Hz, 1H, H_5_), 7.08 (s, 2H, H_13+17_), 5.09 (s, 2H, H_21_), 3.98 (d, *J* = 13.0 Hz, 2H, H_24/25_), 3.74 (t, *J* = 11.6 Hz, 2H, H_24/25_), 3.37 (d, *J* = 11.2 Hz, 2H, H_23/26_), 3.33 (d, *J* = 10.6 Hz, 2H, H_23/26_), 2.14 (s, 6H, H_19+20_) ppm. ^13^C NMR (151 MHz, DMSO-*d*_6_): *δ* 158.80 (C_2_), 152.41 (C_6_), 141.87 (C_4_), 130.38 (C_5_), 127.21 (C_3_), 124.84 (C_13_ + C_17_), 63.55 (C_21_), 58.14 (C_24+25_), 51.41 (C_23+26_), 16.97 (C_19+20_) ppm. Specific details can be found in the ESI.† Single crystals suitable for X-ray diffraction measurements were obtained by slow evaporation of methanolic solution of 8. IR (ATR, selected bands, *υ̃*_max_): 3196, 1605, 1481, 1416, 1215, 1112, 964, 906, 773, 738, 490 cm^−1^. UV–vis (MeOH), *λ*_max_, nm (*ε*, M^−1^ cm^−1^): 265sh, 288 (11 619), 459 (6925), 580sh.

### Physical measurements

Elemental analysis was carried out with a Carlo-Erba microanalyzer at the Microanalytical Laboratory at the Faculty of Chemistry, University of Vienna. The samples for electrospray ionization mass spectrometry (ESI-MS) were measured on an Amazon speed ETD Bruker instrument. Expected and experimental isotope distributions were compared. IR spectra were recorded on a Bruker Vertex 70 Fourier transform IR spectrometer (300–4000 cm^−1^) using attenuated total reflection (ATR) technique. 1D (^1^H, ^13^C) and 2D (^1^H–^1^H COSY, ^1^H–^13^C HSQC, ^1^H–^13^C HMBC) NMR spectra were acquired on a Bruker AV NEO 500 or AV III 600 spectrometers in DMSO-*d*_6_ at 25 °C.

#### Crystallographic structure determination

X-ray diffraction measurements of 1, 3–8 and **[H**_**2**_***L***^**1′**^**]NO**_**3**_ were performed on STOE Stadivari and Bruker X8 APEX-II CCD diffractometers. Single crystals were positioned at 50, 50, 50, 50, 40, 50, 40 and 60 mm from the detector, and 2974, 2650, 3217, 4779, 602, 4389, 3103 and 10 614 frames were measured, each for 20, 20, 60, 5, 50, 30, 4 and 5 s over 0.36, 0.36, 0.36, 0.36, 2.0, 0.36, 0.5 and 1.0° scan width, respectively. Crystal data, data collection parameters, and structure refinement details are given in Tables S1 and S2.† The structures were solved by direct methods and refined by full-matrix least-squares techniques. Non-H atoms were refined with anisotropic displacement parameters. H atoms were inserted in calculated positions and refined with a riding model. The disorder of interstitial solvent in voids of 1, 4 and 5 could not be resolved, and, therefore, SQUEEZE routine implemented in PLATON^72^ was applied to analyze the data, revealing a void volume of 279 Å^3^ (for 1), 75 Å^3^ (for 4), 290 Å^3^ (for 5) and (899 Å^3^) (for 6). The ascertained void content was removed from the model and was not included in the final refinement. The following computer programs and hardware were used: structure solution, SHELXS-2014 and refinement, SHELXL-2014;^73^ molecular diagrams, ORTEP;^74^ computer, Intel CoreDuo. CCDC 2354137 (1), 2354138 (3), 2354139 (4), 2354140 (5), 2354141 (6), 2354142 (7), 2354143 (8) and 2354144**[H**_**2**_***L***^**1′**^**]NO**_**3**_.†

#### Electrochemistry

Cyclic voltammetric experiments with 0.5 mM solutions of 1–3 in 0.1 M *n*Bu_4_NPF_6_ (puriss quality from Fluka (Schwerte, Germany), dried under reduced pressure at 70 °C for 24 h before use) supporting electrolyte in DMSO (SeccoSolv max. 0.025% H_2_O, Merck) were performed under argon atmosphere using a three-electrode setup with a platinum disk or a glassy carbon disk working electrode (from Ionode, Australia), platinum wire as a counter electrode, and silver wire as a pseudo reference electrode. All potentials in voltammetric studies were quoted *vs.* ferricenium/ferrocene (Fc^+^/Fc) redox couple. A Heka PG310USB (Lambrecht, Germany) potentiostat with a PotMaster 2.73 software package served for the potential control in voltammetric studies. *In situ* ultraviolet-visible-near-infrared (UV–Vis–NIR) spectroscopic and spectroelectrochemical measurements were performed on a spectrometer Avantes (Model AvaSpec-2048_14-USB2) in 1 cm quartz cuvette or the spectroelectrochemical cell kit (AKSTCKIT3) with the Pt-microstructured honeycomb working electrode, purchased from Pine Research Instrumentation (Lyon, France). The cell was positioned in the CUV-UV Cuvette Holder (Ocean Optics, Ostfildern, Germany) connected to the diode-array UV–Vis–NIR spectrometer by optical fibres. UV–Vis–NIR spectra were processed using the AvaSoft 7.7 software package. Halogen and deuterium lamps were used as light sources (Avantes, Model AvaLight-DH-S-BAL, Apeldoorn, The Netherlands). EPR spectra were measured with a X-band cw-EPR spectrometers EMX and EMX plus (Bruker) at room temperature or 100 K. Spectroelectrochemical experiments were performed in a flat cell equipped with a Pt mesh working electrode using *n*Bu_4_NPF_6_/DMSO electrolyte. Measurements in frozen solvent glass were done in MeCN/DMF 1 : 1 v/v. Complexes 4 and 5 were dissolved under ambient conditions, and complex 3 was reduced with a stoichiometric equivalent of cobaltocene in a N_2_ glovebox (N_2_ < 2 ppm, H_2_O < 1 ppm). The MeCN/DMF solutions were flash frozen in liquid N_2_ prior to transfer to the N_2_ flow cryostat precooled at 100 K inside the cavity of the EPR resonator. EPR spectra were analyzed with the Easyspin toolbox,^75^ running on Matlab.

### NCI-60 screening

The NCI-60 SRB assay was performed as described previously.^76^ GI_50_ values (the concentration of the metal complex causing 50% growth inhibition), TGI value (the concentration of the complex causing 0% cell growth), and LC_50_ (the concentration of the complex causing 50% cell death) were interpolated from dose–response curves that were plots of percentage cell growth *versus* concentration of test compounds.

#### Pharmacological analysis of selected compounds

The biological effects of selected metal complexes (1, 2, 4 and 5) and of their proligands (HL^1^, HL^2^ and HL^3^) were tested more extensively on A549 human lung adenocarcinoma cells and on MRC-5 human lung fibroblast cells. Both cell lines were obtained from ATCC and maintained in DMEM cell culture media containing 1.0 g L^−1^ glucose (Biosera, Nuaille, France) complemented with 10% fetal bovine serum (FBS), 2 mM l-glutamine, 0.01% streptomycin and 0.005% penicillin and were cultured under standard conditions in a 37 °C incubator at 5% CO_2_ and 95% humidity. The viability of A549 and of MRC-5 fibroblast cells upon exposure to the selected metal–ligand complexes and ligands were evaluated by the MTT assay. For this, 10 000 cells per well were seeded into 96-well plates and left to grow. On the next day the cells were exposed to 0, 5, 10, 20, 40 or 80 μM of each compound or to an equivalent volume of DMSO for 24 h. After the treatments, cells were washed with PBS and then were incubated with 0.5 mg mL^−1^ MTT reagent (Sigma-Aldrich, St Louis, Missouri, USA) for 1 h at 37 °C. Finally, 100 μL of DMSO (Molar Chemicals, Halásztelek, Hungary) was added to each well, and the absorbance of samples was measured at 570 nm using a Synergy HTX plate reader (BioTek, Winooski, Vermont, USA). The viability measurements were repeated three times using 3 independent biological replicates.

#### BrdU assay

The effect of proligands HL^1^–HL^3^ and of metal complexes 1, 2 and 4, 5 on the proliferation of A549 cells was investigated by the BrdU assay (5-bromo-2′-deoxyuridine Labeling and Detection Kit I, Roche, Cat. No. 11 296 736 001). For this, 3 × 10^4^ cells were seeded onto glass coverslips that were placed on 24-well plates, and the cells were left to grow overnight. Next day, when the cells reached about 50% confluence, the samples were treated with 10 μM of each compound or with an equivalent volume of DMSO for 24 h. At this point, BrdU labeling solution was added in a 1 : 1000 dilution, and the incubation continued for 30 min. Then the cells were fixed using 70% ethanol in 50 mM glycine (pH 2.0), and immunofluorescence of adherent cells was measured following the manufacturer's instructions. BrdU incorporation was visualized using an Olympus FV10i confocal microscope.

#### Tubulin assays

The methods used for determination of the IC_50_ values for tubulin assembly and inhibition of [^3^H]colchicine binding were as described previously.^77^

#### Tubulin immunostaining

The structure of microtubules was visualized by α-tubulin immunostaining on A549 cells. For this, 5 × 10^4^ cells per well were seeded onto coverslips in 24-well plates. On the following day the samples were treated with 80 μM of each compound or with an equivalent amount of DMSO for 24 h. Then samples were fixed with 4% formaldehyde (Molar Chemicals, Halásztelek, Hungary), permeabilized with 0.3% Triton-X-100 (Calbiochem, Merck Millipore, Darmstadt, Germany) and were blocked using 5% BSA (Sigma-Aldrich, St Louis, Missouri, USA) diluted in PBS. Then the cells were incubated with anti-α-tubulin antibody (Thermo Fisher Scientific, monoclonal mouse antibody, DM1A, Waltham, Massachusetts, USA) in a 1 : 300 dilution in 1% BSA followed by Alexa 488 fluorophore-conjugated goat anti-mouse secondary antibody (Abcam, Cambridge, UK) in a 1 : 600 dilution in 1% BSA. The stained samples were examined in an Olympus FV10i confocal microscope.

#### Molecular docking

The complexes were docked against the crystal structure of the tubulin-colchicine site (PDB ID: 4O2B, resolution 2.30 Å),^63^ which was obtained from the Protein Data Bank (PDB).^78,79^ The GOLD (v2024.1) software suite was used to prepare the crystal structures for docking, *i.e.*, the hydrogen atoms were added, water molecules and expedients deleted and the co-crystallised ligand identified: tubulin-colchicine site – LOC. The docking center for the binding pocket was defined as the position of the co-crystallised LOC ligands with a 10 Å radius. The scoring functions GoldScore(GS),^65^ ChemScore(CS),^66,67^ Piecewise Linear Potential (ChemPLP)^68^ and Astex Statistical Potential (ASP)^69^ were used in the GOLD (v2024.1) docking algorithm. The crystal structures of the complexes 4 and 8 were used for the docking.

## Author contributions

Iuliana Besleaga – data curation; formal analysis; investigation; methodology; writing – original draft. Renáta Raptová – data curation; investigation; methodology; Alexandru-Constantin Stoica – data curation; software; visualization; investigation; Miljan N. M. Milunovic – writing – original draft; formal analysis; methodology; Michal Zalibera – investigation; methodology; software; validation; Ruoli Bai – data curation; investigation; Nóra Igaz – data curation; investigation; methodology; Jóhannes Reynisson – data curation; formal analysis; investigation; methodology; writing – original draft; Mónika Kiricsi – investigation; methodology; software; validation; Éva A. Enyedy – data curation; formal analysis; investigation; methodology; writing – original draft; writing – review and editing; Peter Rapta – investigation; methodology; writing – original draft; Ernest Hamel – investigation; writing – review; and Vladimir B. Arion – conceptualization; funding acquisition; investigation; project administration; writing – review and editing.

## Disclaimer

This research was supported in part by the Developmental Therapeutics Program in the Division of Cancer Treatment and Diagnosis of the National Cancer Institute, which includes federal funds under Contract No. HHSN261200800001E. The content of this publication does not necessarily reflect the views or policies of the Department of Health and Human Services, nor does mention of trade names, commercial products, or organizations imply endorsement by the U.S. Government.

## Data availability

The data supporting this article have been included as part of the ESI.† Crystallographic data for compounds 1, 3, 4, 5, 6, 7, 8 and **[H**_**2**_***L***^**1′**^**]NO**_**3**_ has been deposited at the CCDC under accession numbers 2354137, 2354138, 2354139, 2354140, 2354141, 2354142, 2354143 and 2354144**[H**_**2**_***L***^**1′**^**]NO**_**3**_ and can be obtained from CCDC e-mail: deposit@ccdc.cam.ac.uk.†

## Conflicts of interest

There are no conflicts to declare.

## Supplementary Material

DT-053-D4DT01469C-s001

DT-053-D4DT01469C-s002
