# Supplementary material for: Are the metal identity and stoichiometry of metal complexes important for colchicine site binding and inhibition of tubulin polymerization?
Source: Dalton Trans. 2024 Jul 2;53(29):12349–69. doi: 10.1039/d4dt01469c (PMC11264232; doi:10.1039/d4dt01469c)
Supplement: DT-053-D4DT01469C-s001 [file DT-053-D4DT01469C-s001.pdf]

Electronic Supplementary Material (ESI) for Dalton Transactions.
This journal is © The Royal Society of Chemistry 2024

Electronic Supplementary Information
for

Are the metal identity and stoichiometry of metal complexes important for colchicine site binding and inhibition of tubulin polymerization?

Iuliana Besleaga,[a] Renáta Raptová,[b,c] Alexandru-Constantin Stoica,[d] Miljan N. M. Milunovic,[a,*] Michal Zalibera,[b] Ruoli Bai,[e] Nóra Igaz,[f] Jóhannes Reynisson,[g] Mónika Kiricsi,[g] Éva A. Enyedy,[h,i,*] Peter Rapta,[b] Ernest Hamel,[e] Vladimir B. Arion[a,d,*]

[a]*Institute of Inorganic Chemistry, University of Vienna, A-1090 Vienna, Austria*

[b]*Institute of Physical Chemistry and Chemical Physics, Faculty of Chemical and Food Technology, Slovak University of Technology in Bratislava, SK-81237 Bratislava, Slovakia*

[c]*Institute of Physical and Theoretical Chemistry, Graz University of Technology, Stremayrgasse 9/II, A-8010 Graz, Austria*

[d]*Inorganic Polymers Department, "Petru Poni" Institute of Macromolecular Chemistry, Aleea Gr. Ghica Voda 41 A, Iasi 700487, Romania*

[e]*Molecular Pharmacology Branch, Developmental Therapeutics Program, Division of Cancer Diagnosis and Treatment, National Cancer Institute, Frederick National Laboratory for Cancer Research, National Institutes of Health, Frederick, Maryland 21702, United States*

[f]*Department of Biochemistry and Molecular Biology, University of Szeged, Közép fasor 52, H-6726 Szeged, Hungary*

[g]*School of Pharmacy and Bioengineering, Keele University, Newcastle-under-Lyme, Staffordshire ST5 5BG, United Kingdom*

[h]*Department of Molecular and Analytical Chemistry, Interdisciplinary Excellence Centre, University of Szeged, Dóm tér 7-8, H-6720 Szeged, Hungary*

[i]*MTA-SZTE Lendület Functional Metal Complexes Research Group, University of Szeged, Dóm tér 7, H-6720 Szeged, Hungary*

Table of Contents

Are the metal identity and stoichiometry of metal complexes important for colchicine site binding and inhibition of tubulin polymerization? 1

1. NMR Data....................3
2. Crystallographic data collection 18
3. Solution Stability Studies 20
4. Spectroelectrochemistry 23
5. NCI-60 One-Dose Screen....................28
6. NCI-60 5-Dose Screen....................34
7. Molecular docking....................38

1. NMR Data

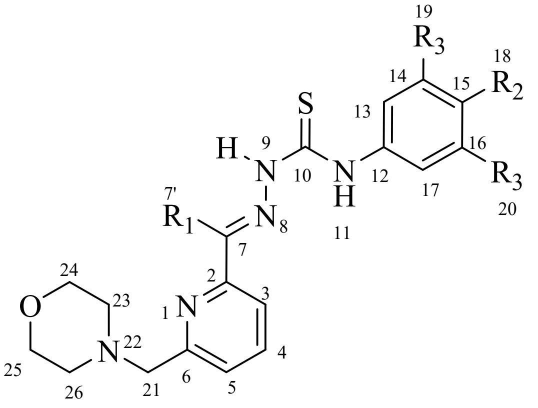

E-isomer

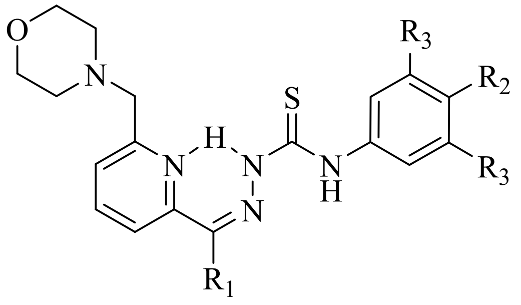

Z-isomer

Chart S1. Atom numbering of ligands for the assignment of NMR resonances.

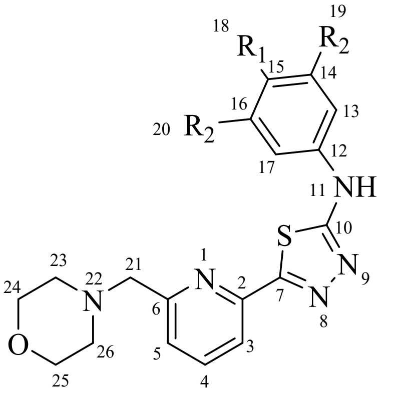

R_1 = OH, R_2 = Me **(H*L*[1'])**

R_1 = H, R_2 = H **(H*L*[3'])**

Chart S2. Line drawings of oxidized TSCs and their atom numbering for the assignment of NMR resonances.

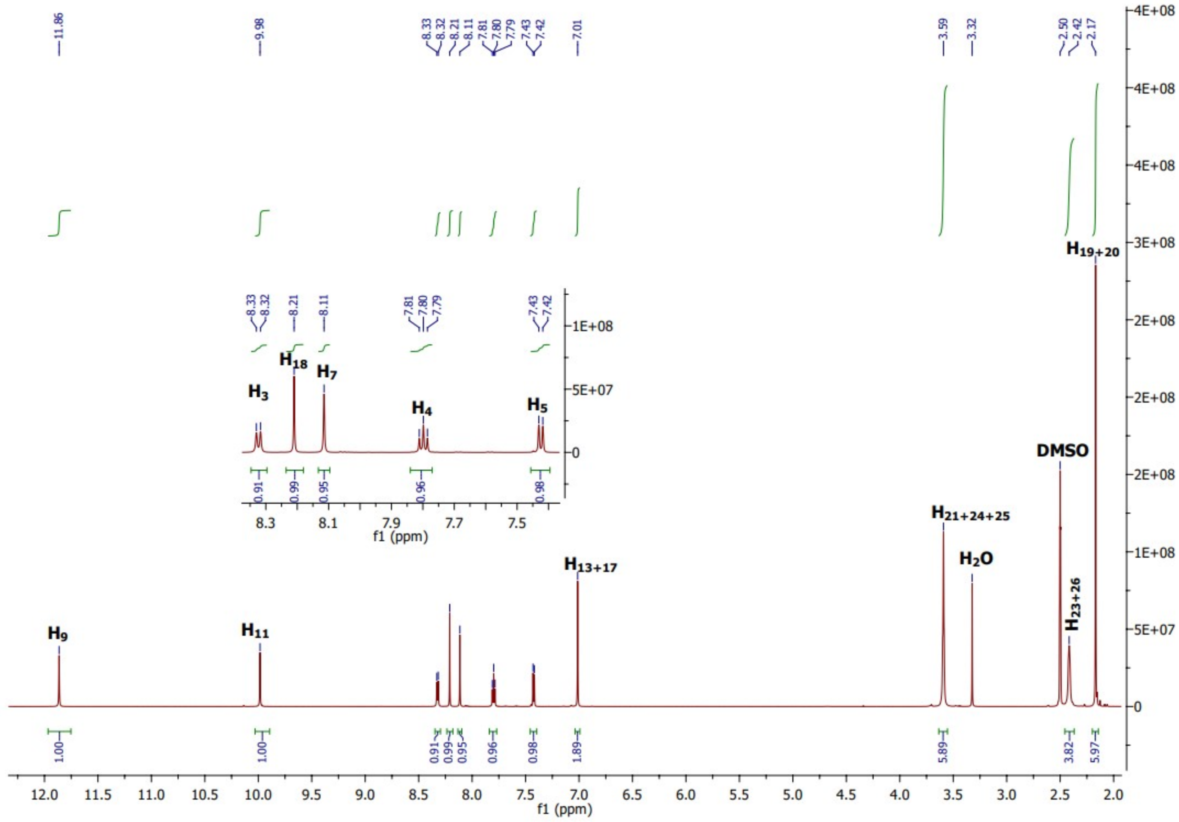

Figure S1A. ^{1}H NMR spectrum of **HL^{1}**.

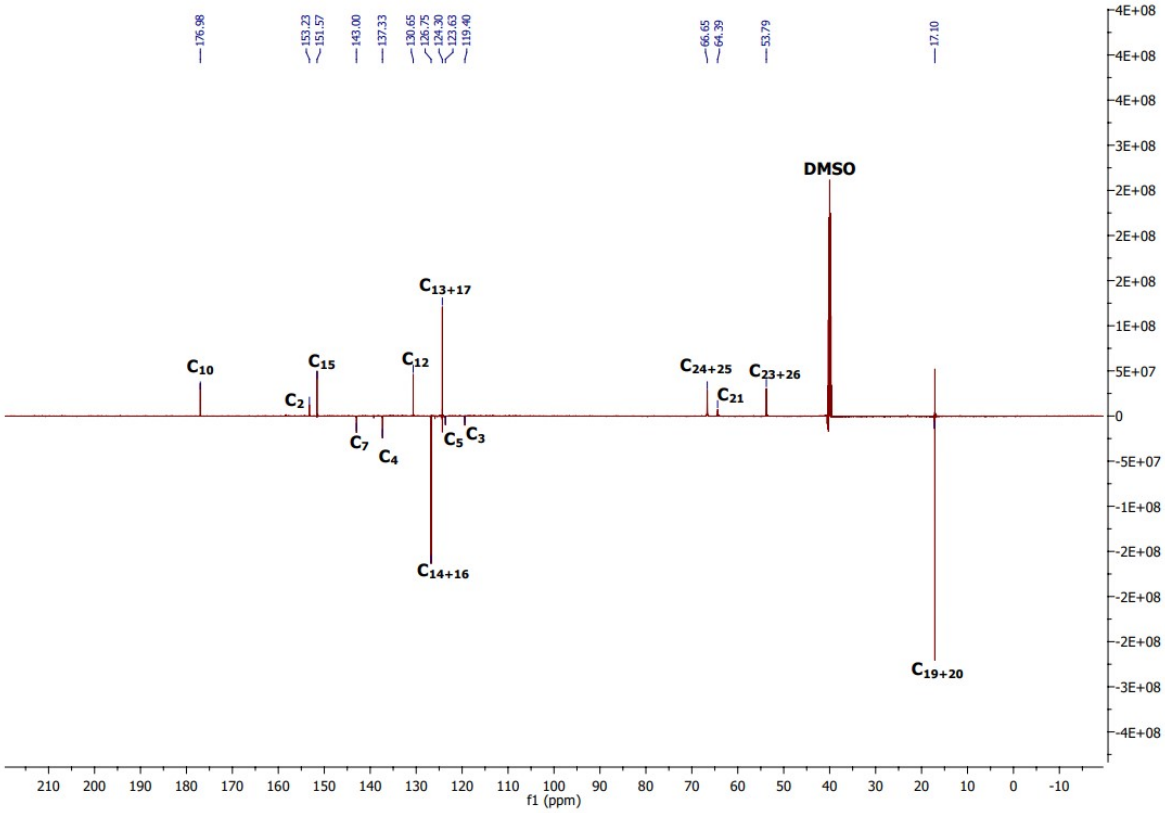

Figure S1B. ^{13}C NMR spectrum of **HL^{1}**.

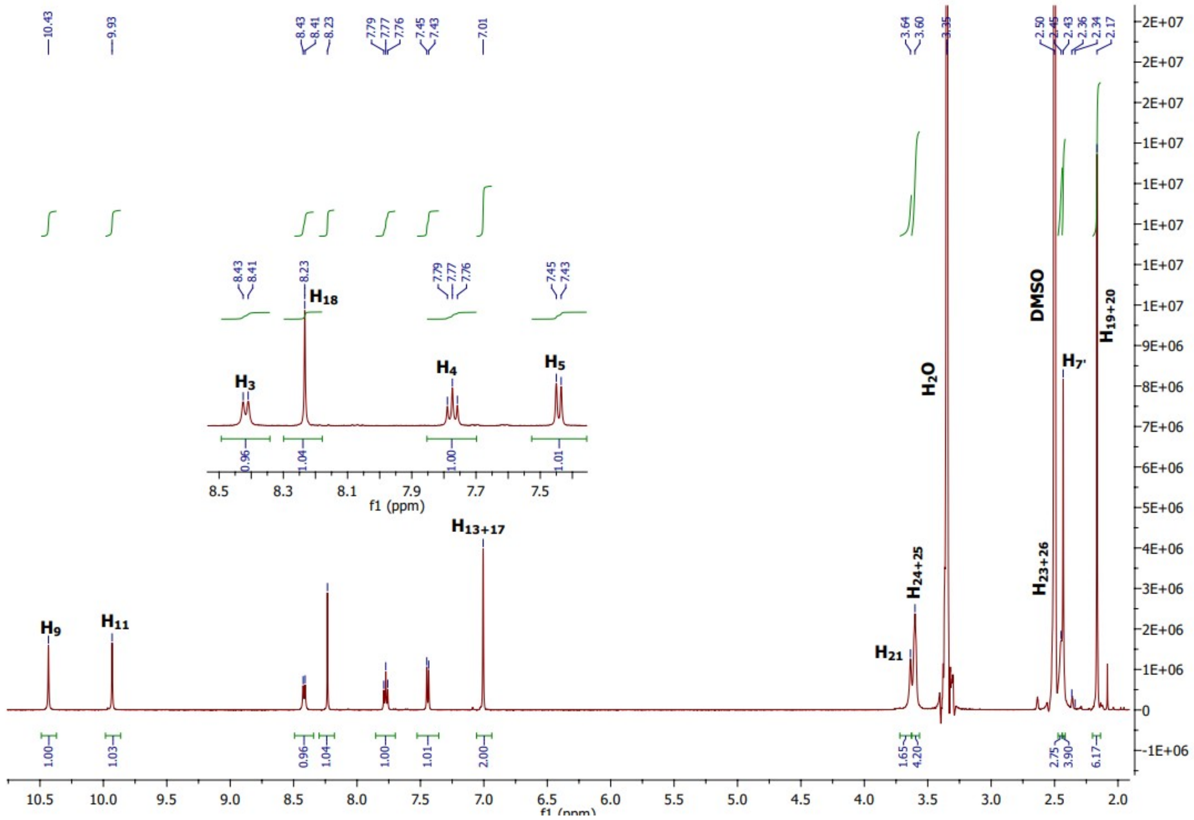

Figure S2A. ^{1}H NMR spectrum of $\mathbf{HL^2}$.

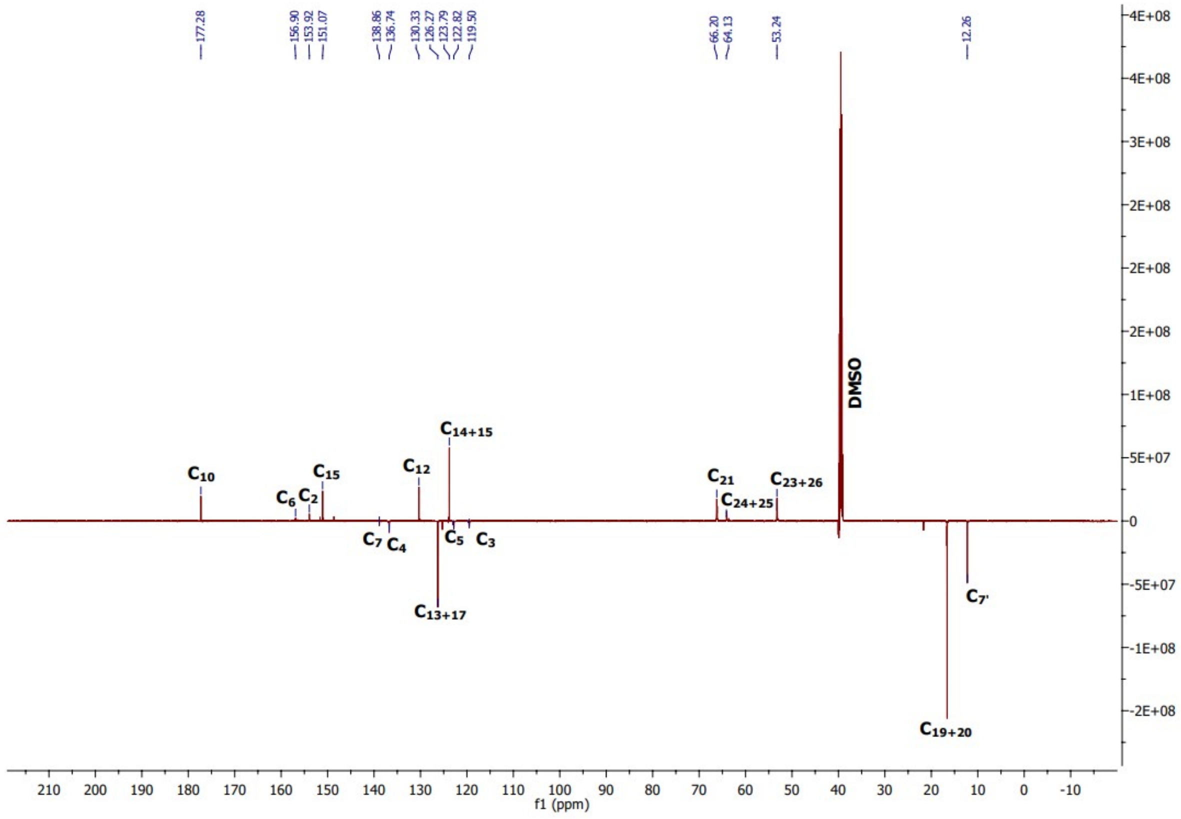

Figure S2B. ^{13}C NMR spectrum of $\mathbf{HL^2}$.

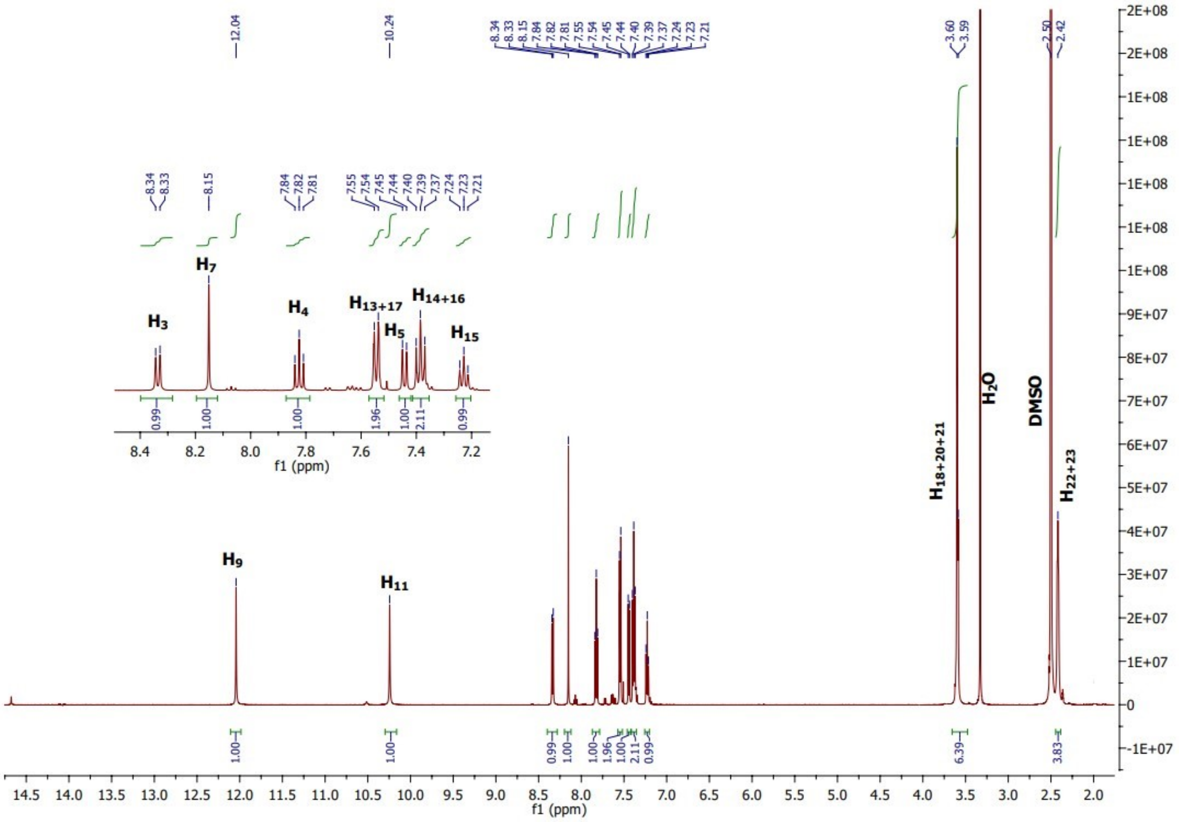

Figure S3A. ^{1}H NMR spectrum of **HL^{3}**.

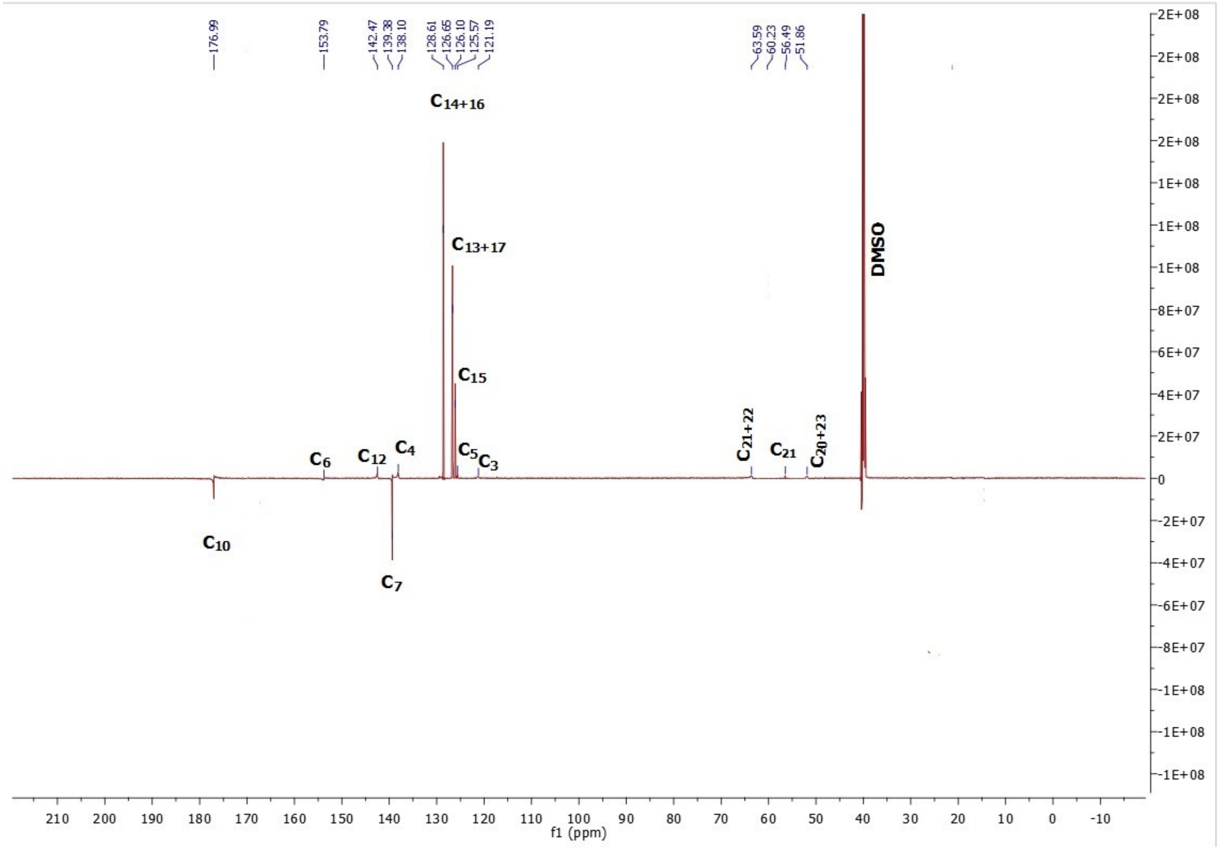

Figure S3B. ^{13}C NMR spectrum of **HL^{3}**.

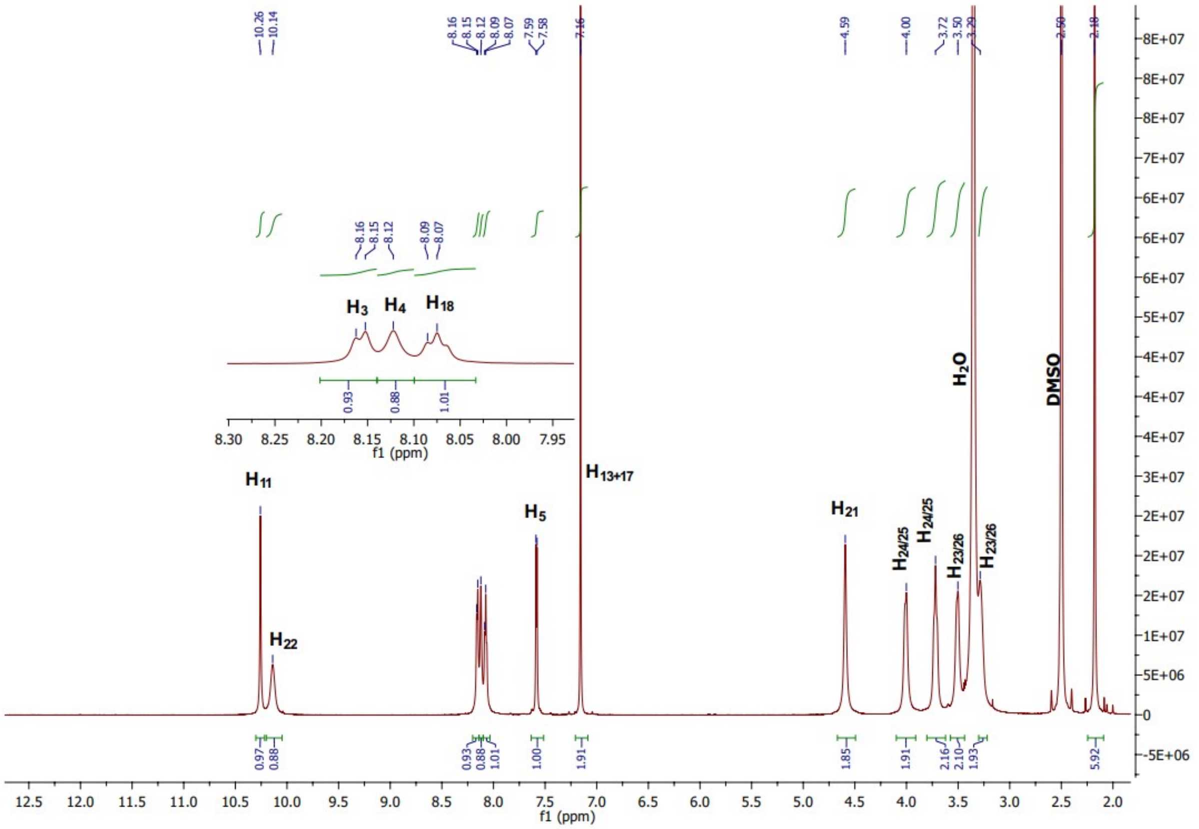

Figure S4A. ^{1}H NMR spectrum of **H*L*$^{1'}$**.

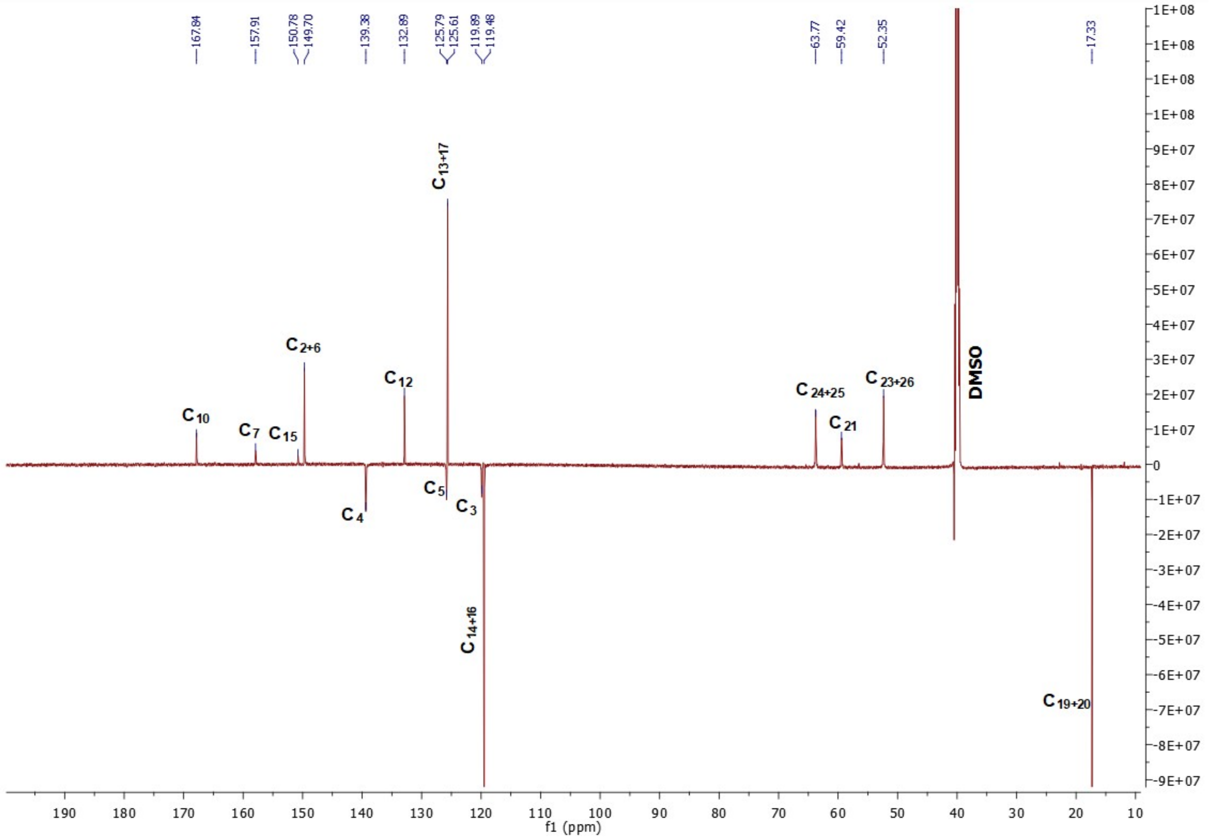

Figure S4B. ^{13}C NMR spectrum of **H*L*$^{1'}$**.

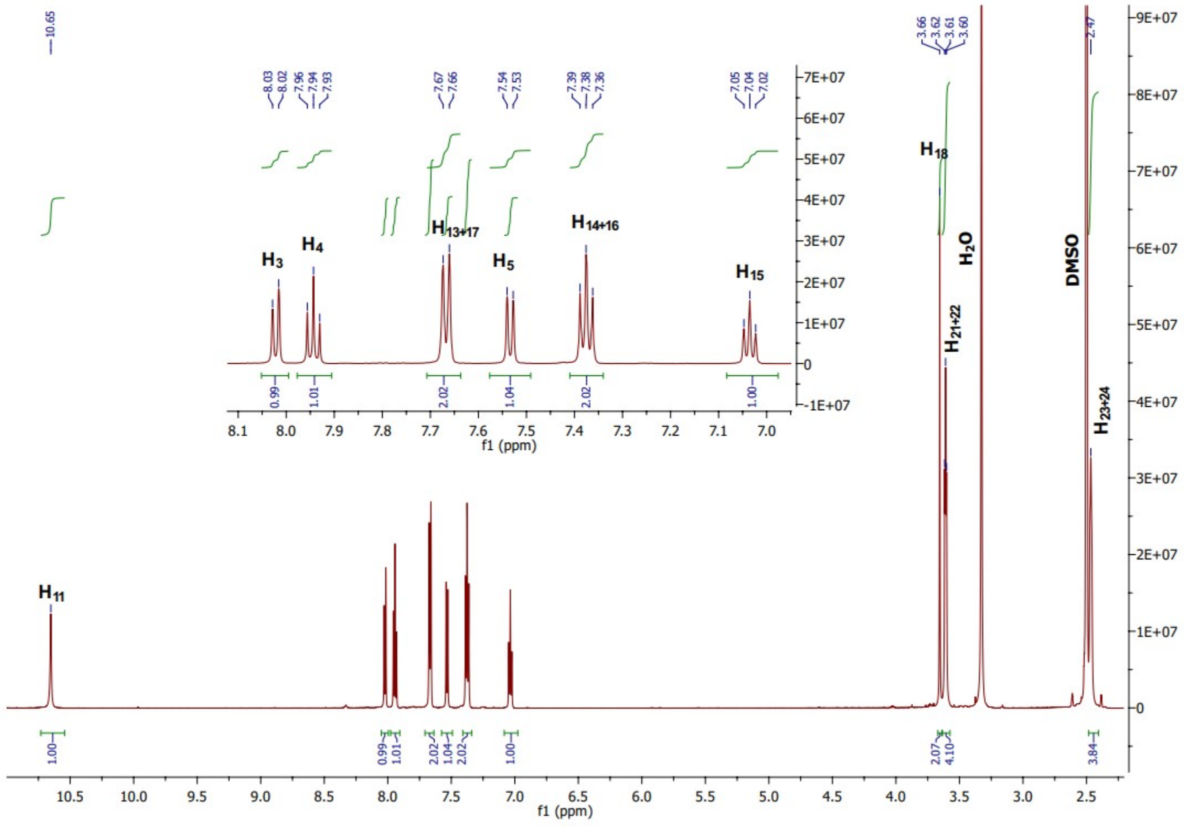

Figure S5A. ^{1}H NMR spectrum of **H*L*$^{3'}$**.

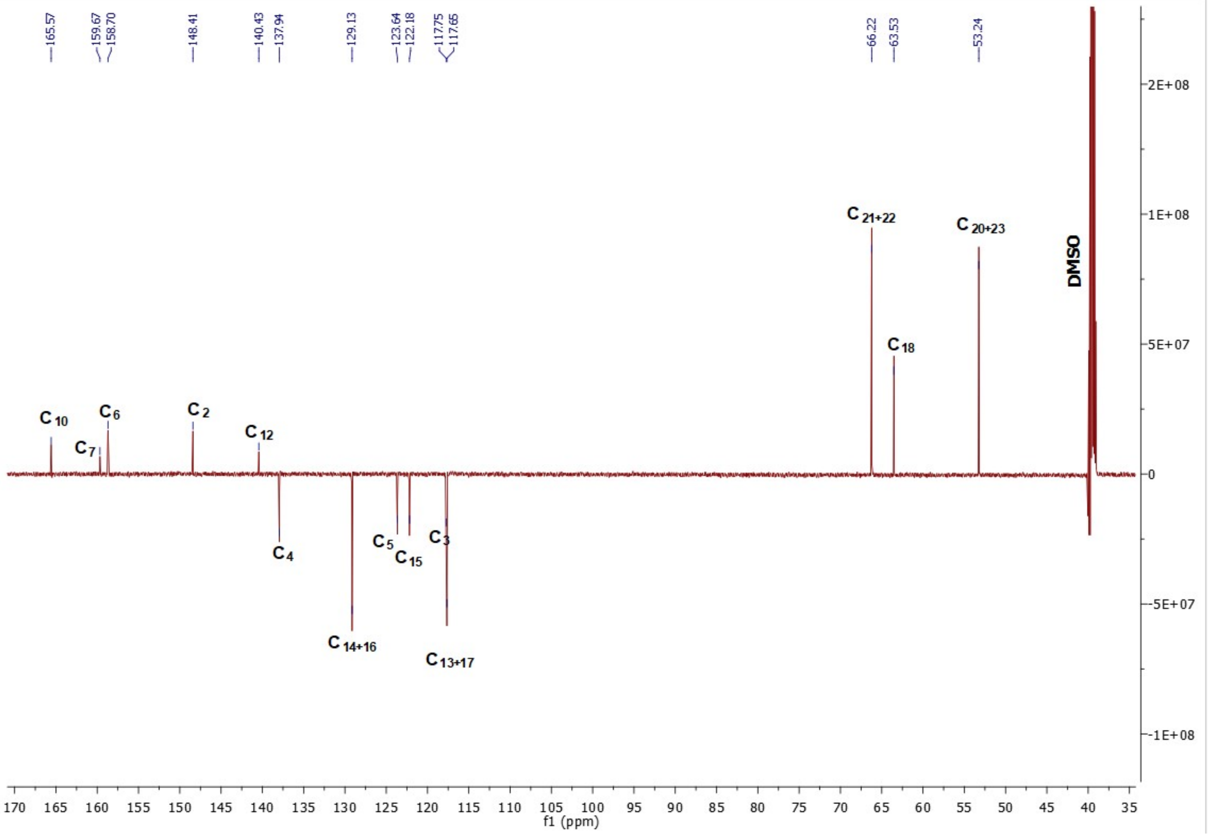

Figure S5B. ^{13}C NMR spectrum of **H*L*$^{3'}$**.

Assignment of proton resonances in ^{1}H NMR spectra of the morp-TSCs (solvent: DMSO-d_6).

		**HL^1** (*E*-)	**H*L*$^{1'}$**	**HL^2** (*E*-)	**HL^3** (*E*-)	**H*L*$^{3'}$**
H_3	C*H*$_{py}$	8.32 (d, 1H)	8.16 (d, 1H)	8.42 (d, 1H)	8.34 (s, 1H)	8.02 (d, 1H)
H_4	C*H*$_{py}$	7.80 (t, 1H)	8.08 (d, 1H)	7.77 (t, 1H)	7.82 (s, 1H)	7.95 (d, 1H)
H_5	C*H*$_{py}$	7.42 (d, 1H)	7.58 (d, 1H)	7.44 (d, 1H)	7.54 (s, 1H)	7.53 (d, 1H)
H_7	C*H*=N	8.11 (s, 1H)	-	-	8.15 (s, 1H)	-
$H_{7'}$	C*H*$_3$(C=N)	-	-	2.45 (s, 3H)	-	-
H_9	N*H* (closer to py)	11.86 (s, 1H)	-	10.43 (s, 1H)	12.04 (s, 1H)	-
H_{11}	N*H* (closer to ph)	9.98 (s, 1H)	10.26 (s, 1H)	9.93 (s, 1H)	10.24 (s, 1H)	10.65 (s, 1H)
H_{13+17}	C*H*$_{ph}$	7.01 (s, 2H)	7.16 (s, 2H)	7.01 (s, 2H)	7.44 (d, 2H)	7.67 (d, 2H)
H_{14+16}	C*H*$_{ph}$	-	-	-	7.39 (t, 2H)	7.38 (t, 2H)
H_{15}	C*H*$_{ph}$	-	-	-	7.23 (t, 1H)	7.04 (s, 1H)
H_{18}	O*H*$_{ph}$	8.21 (s, 1H)	8.12 (s, 1H)	8.23 (s, 1H)	-	-
H_{19+20}	C*H*$_3$	2.17 (s, 6H)	2.18 (s, 6H)	2.17 (s, 6H)	-	-
H_{22} (H_{19} for **HL^3** and **H*L*$^{3'}$**)	N*H*$_{(morph)}$	-	10.14 (s, 1H	-	11.38 (s, 1H)	-
H_{21} (H_{18} for **HL^3** and **H*L*$^{3'}$**)	C*H*$_2$–N$_{(morph)}$	3.59 (s, 6H)	4.59 (s, 2H)	3.64 (s, 2H)	3.59 (s, 6H)	3.66 (s, 2H)
H_{24+25} (H_{21+22} for **HL^3** and **H*L*$^{3'}$**)	C*H*$_{2\ (morph)}$ (closer to O)		4.00 (s, 2H) 3.72 (s, 2H)	3.60 (s, 4H)		3.61 (m, 4H)
H_{23+26} (H_{20+23} for **HL^3** and **H*L*$^{3'}$**)	C*H*$_{2\ (morph)}$ (closer to N)	2.42 (s, 4H)	3.50 (s, 2H) 3.29 (s, 2H)	2.43 (s, 4H)	2.42 (s, 4H)	2.47 (s, 4H)

Assignment of ^{13}C resonances in ^{13}C NMR spectra of the morp-TSCs (solvent: DMSO-d_6).

		**HL^1** (*E*-)	**H*L*$^{1'}$**	**HL^2** (*E*-)	**HL^3** (*E*-)	**H*L*$^{3'}$**
C_2	C_{py}	153.23	149.67	153.92	149.17*	148.41
C_3	CH_{py}/C_{py}	119.40	119.89	119.50	121.19	117.75
C_4	CH_{py}	137.33	139.38	136.74	138.10	137.94
C_5	CH_{py}	123.63	125.79	122.82	125.57	123.64
C_6	CH_{py}	158.16*	149.70	156.90	153.76	158.70
C_7	*C*=N	143.00	-	138.86	139.38	-
C_7	thiadiazole	-	157.91	-	-	159.67
$C_{7'}$	CH_3 (C=N)	-	-	12.26	-	-
C_{10}	*C*=S	176.98	-	177.28	176.99	-
C_{10}	S-*C*-N	-	167.84	-	-	165.57
C_{12}	C_{ph}	130.65	132.89	130.33	142.47	140.43
C_{13+17}	CH_{ph}	126.75	119.48	126.27	126.65	117.65
C_{14+16}	$C(CH_3)_{ph}$	124.30	125.61	123.79	128.61	129.13
C_{15}	$C(OH)_{ph}$	151.57	150.77	151.07	126.10	122.18
C_{19+20}	$C(CH_3)_{ph}$	17.10	17.33	16.60	-	-
C_{21} (C_{18} for **HL^3** and **H*L*$^{3'}$**)	CH_2–$N_{(morph)}$	64.39	59.42	66.20	60.23	63.53
C_{24+25} (C_{21+22} for **HL^3** and **H*L*$^{3'}$**)	$CH_{2\ (morph)}$ (closer to O)	66.65	63.77	64.13	63.59	66.22
C_{23+26} (C_{20+23} for **HL^3** and **H*L*$^{3'}$**)	$CH_{2\ (morph)}$ (closer to N)	53.79	52.35	53.24	51.86	53.24

*Resonances were assigned by using ^{1}H–^{13}C HSQC, ^{1}H–^{13}C HMBC and ^{1}H–^{1}H COSY NMR spectra.

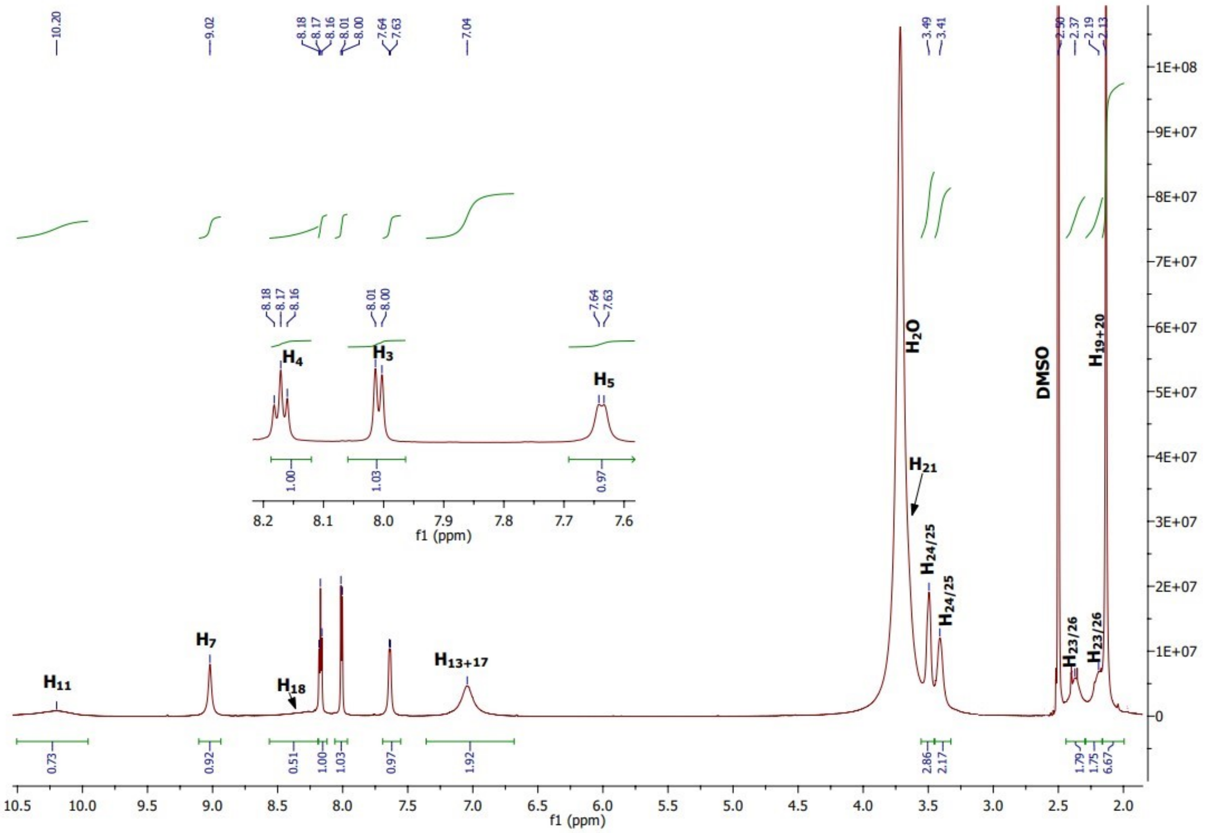

Figure S6A. ^{1}H NMR spectrum of **1**.

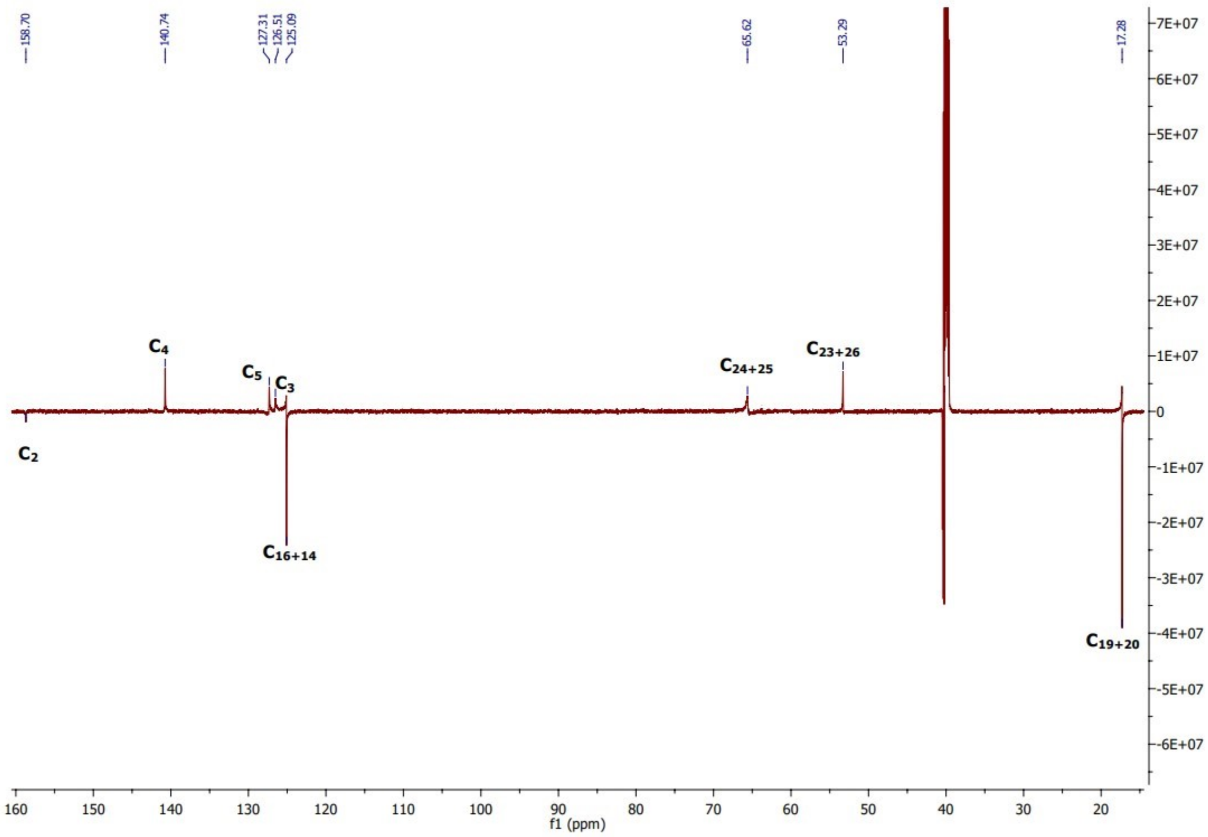

Figure S6B. ^{13}C NMR spectrum of **1**.

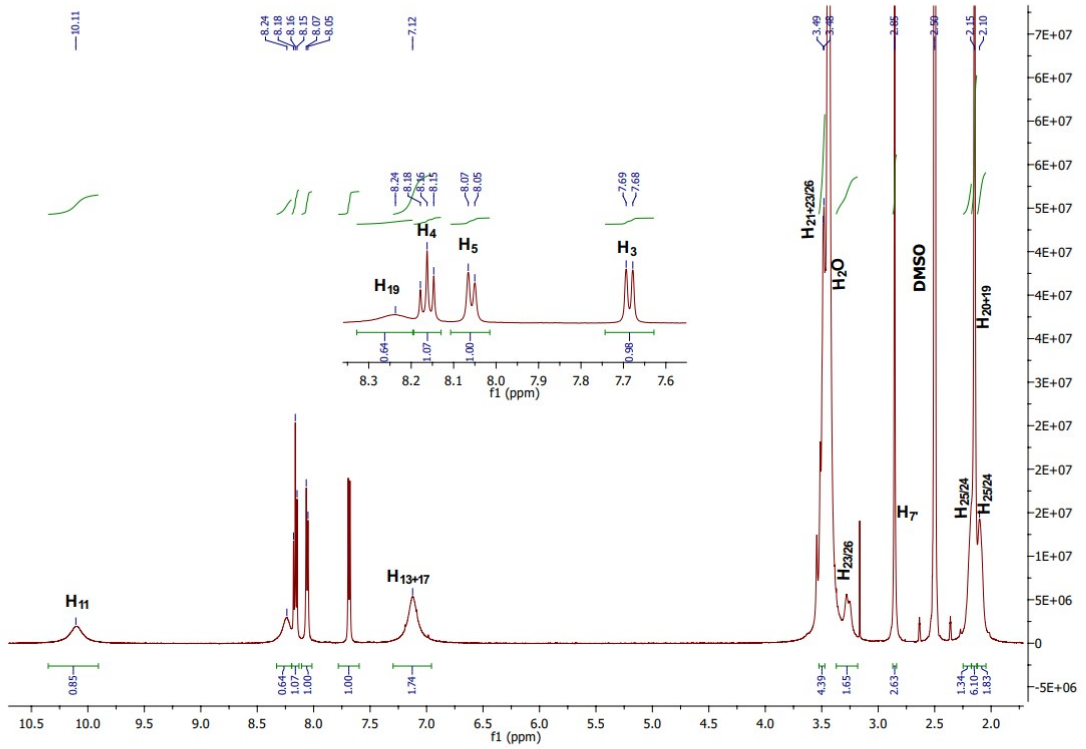

Figure S7A. ^{1}H NMR spectrum of **2.**

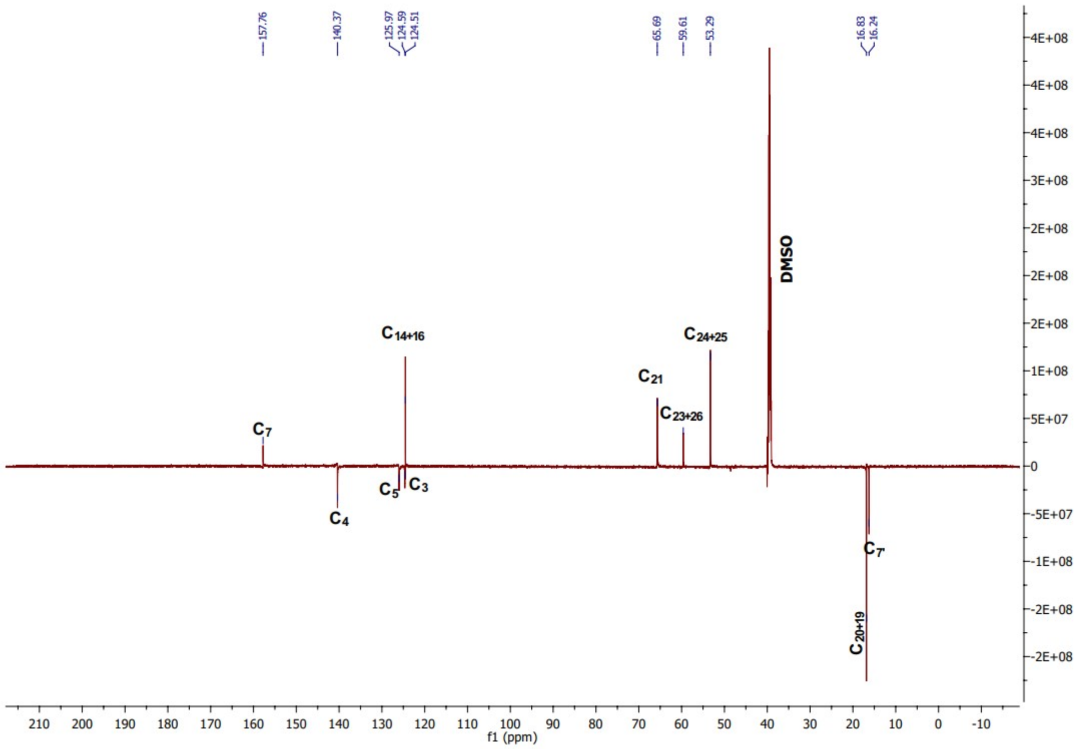

Figure S7B. ^{13}C NMR spectrum of **2.**

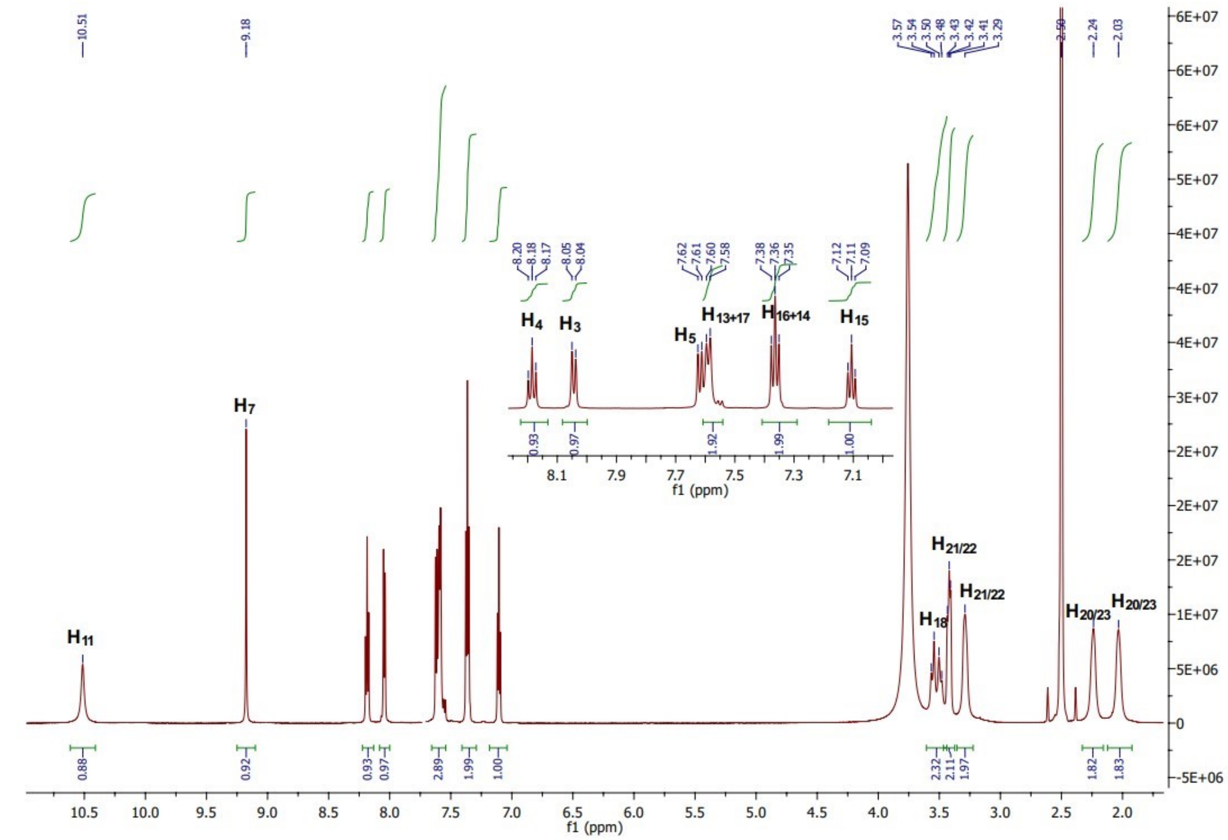

Figure S8A. ^{1}H NMR spectrum of **3.**

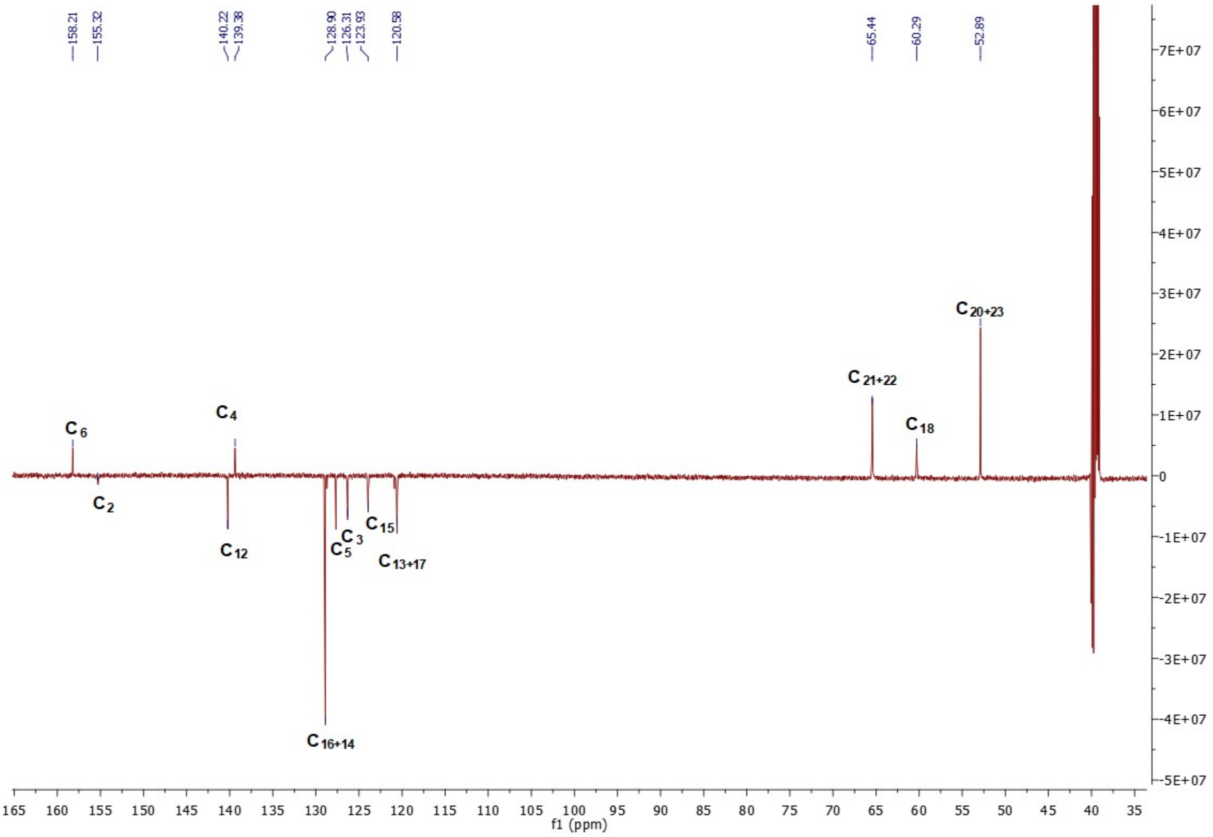

Figure S8B. ^{13}C NMR spectrum of **3**.

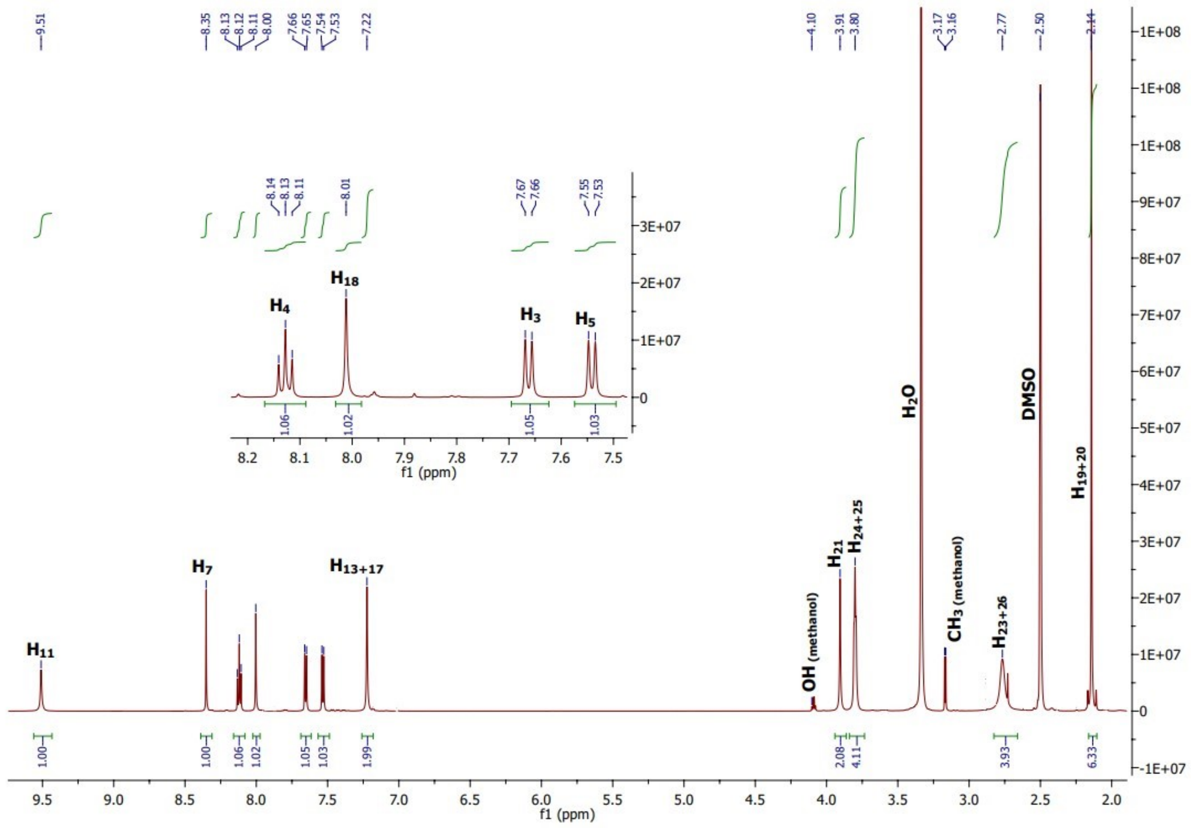

Figure S9A. ^{1}H NMR spectrum of **7**.

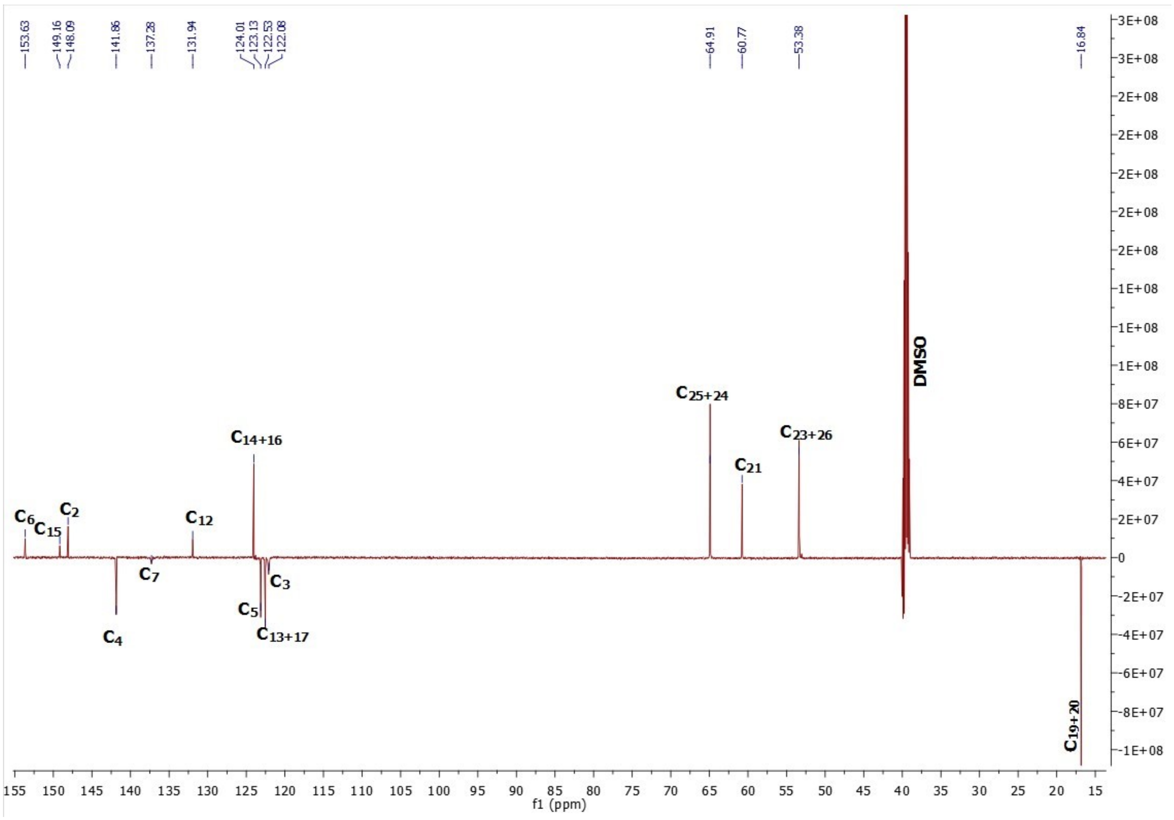

Figure S9B. ^{13}C NMR spectrum of **7**.

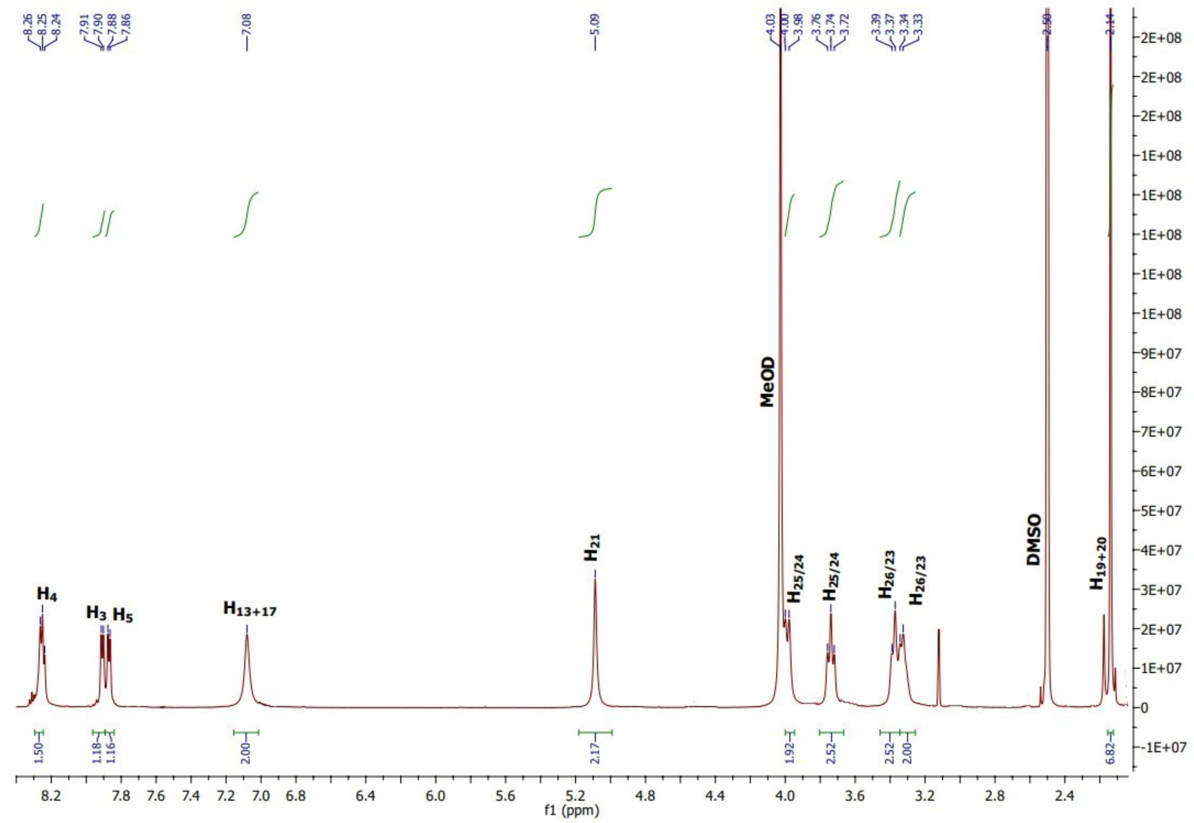

Figure S10A. ^{1}H NMR spectrum of **8**.

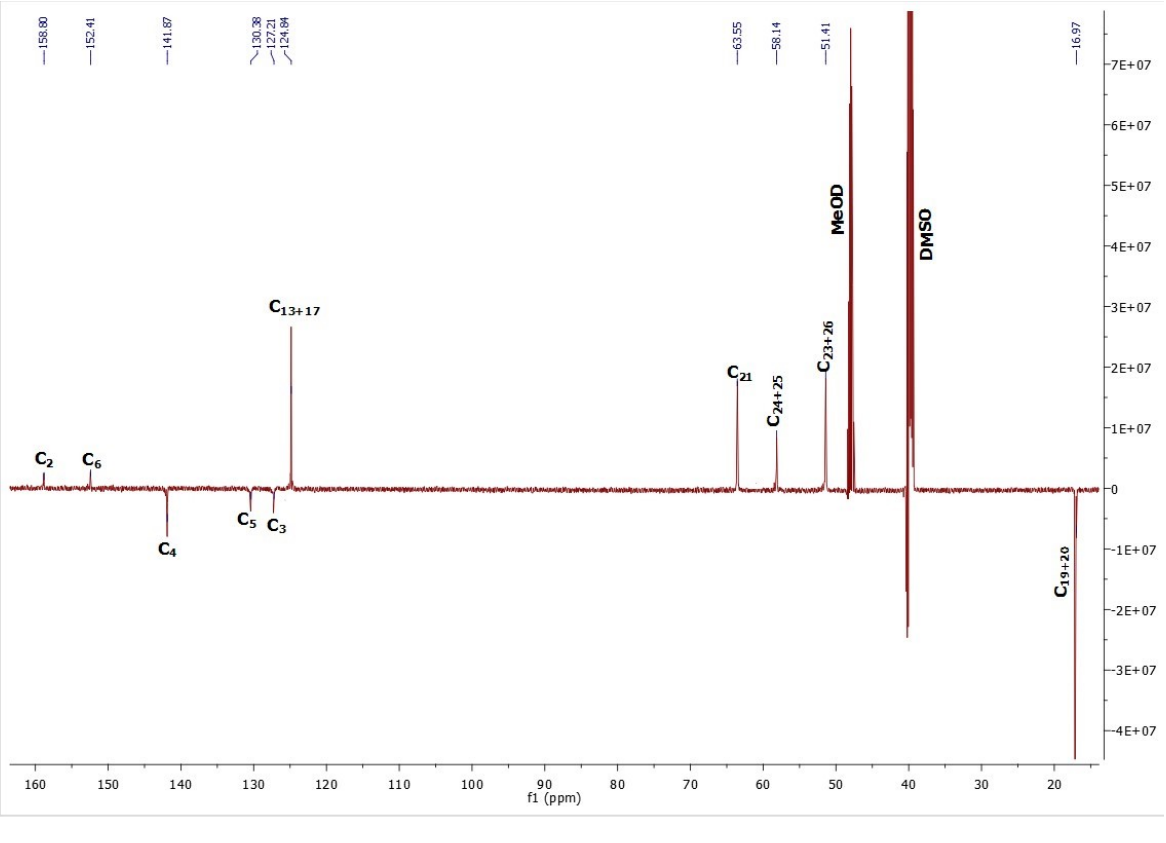

Figure S10B. ^{13}C NMR spectrum of **8**.

Assignment of proton resonances in ^{1}H NMR spectra of **1**, **7** and **8** (solvent: DMSO-d_6).

<table>
<tr><th></th><th></th><th>HL^1 (E-)</th><th>1</th><th>7</th><th>8*</th></tr>
<tr><td>H_3</td><td>CH_{py}</td><td>8.32 (d, 1H)</td><td>8.01 (d, 1H)</td><td>7.66 (d, 1H)</td><td>7.91 (d, 1H)</td></tr>
<tr><td>H_4</td><td>CH_{py}</td><td>7.80 (t, 1H)</td><td>8.17 (t, 1H)</td><td>8.13 (t, 1H)</td><td>8.26 (t, 1H)</td></tr>
<tr><td>H_5</td><td>CH_{py}</td><td>7.42 (d, 1H)</td><td>7.64 (d, 1H)</td><td>7.54 (d, 1H)</td><td>7.87 (d, 1H)</td></tr>
<tr><td>H_7</td><td>CH=N</td><td>8.11 (s, 1H)</td><td>9.02 (s, 1H)</td><td>8.36 (s, 1H)</td><td>not detected</td></tr>
<tr><td>H_9</td><td>NH (closer to py)</td><td>11.86 (s, 1H)</td><td>-</td><td>-</td><td>-</td></tr>
<tr><td>H_{11}</td><td>NH (closer to ph)</td><td>9.98 (s, 1H)</td><td>10.20 (s, 1H)</td><td>9.52 (s, 1H)</td><td>not detected</td></tr>
<tr><td>H_{13+17}</td><td>CH_{ph}</td><td>7.01 (s, 2H)</td><td>7.04 (b, 2H)</td><td>7.23 (s, 2H</td><td>7.08 (s, 2H)</td></tr>
<tr><td>H_{18}</td><td>OH_{ph}</td><td>8.21 (s, 1H)</td><td>8.56 – 8.19 (b, 1H)</td><td>8.01 (s, 1H)</td><td>not detected</td></tr>
<tr><td>H_{19+20}</td><td>CH_{3ph}</td><td>2.17 (s, 6H)</td><td>2.13 (s, 6H)</td><td>2.15 (s, 6H)</td><td>2.14 (s, 6H)</td></tr>
<tr><td>H_{22}</td><td>N$H_{(morph)}$</td><td>-</td><td>-</td><td>-</td><td>-</td></tr>
<tr><td>H_{21}</td><td>CH_2–$N_{(morph)}$</td><td rowspan="2">3.59 (s, 6H)</td><td>3.69 (s, 4H)* + ($H_{24/25}$)</td><td>3.91 (s, 2H)</td><td>5.09 (s, 2H)</td></tr>
<tr><td>H_{24+25}</td><td>C$H_{2\ morph}$
(closer to O)</td><td>3.49 (s, 2H)
3.41 (s, 2H)</td><td>3.81 (s, 4H)</td><td>3.98 (m, 1H)
3.74 (m, 1H)</td></tr>
<tr><td>H_{23+26}</td><td>C$H_{2\ morph}$
(closer to N)</td><td>2.42 (s, 4H)</td><td>2.37 (s, 2H)
2.19 (s, 2H)</td><td>2.77 (s, 4H)</td><td>3.37 (m, 1H)
3.33 (m, 1H)</td></tr>
</table>

*Measured in DMSO-d_6 + 10% MeOD-d_4.

Assignment of ^{13}C resonances in ^{13}C NMR spectra of **1**, **7** and **8** (solvent: DMSO-d_6).

		**HL**$^{\mathbf{1}}$ (*E*-)	**1**	**7**	**8***
C_2	C_{py}	153.23	158.70	148.09	158.80
C_3	CH_{py}/C_{py}	119.40	126.51	122.53	127.21
C_4	CH_{py}	137.33	140.74	141.86	141.87
C_5	CH_{py}	123.63	127.31	123.13	130.38
C_6	CH_{py}	158.16	not detected	153.63	152.41
C_7	*C*=N	143.00	not detected	137.28	not detected
C_{10}	*C*=S	176.98	-	-	-
C_{10}	S-*C*-N	-	not detected	not detected	not detected
C_{12}	C_{ph}	130.65	not detected	131.94	not detected
C_{13+17}	CH_{ph}	126.75	not detected	122.08	124.84
C_{14+16}	$C(CH_3)_{ph}$	124.30	125.09	124.01	not detected
C_{15}	$C(OH)_{ph}$	151.57	151.04*	149.16	not detected
C_{19+20}	$C(CH_3)_{ph}$	17.10	17.28	16.84	16.97
C_{21}	CH_2–$N_{(morph)}$	64.39	60.06*	60.77	63.55
C_{24+25}	$CH_{2\ (morph)}$ (closer to O)	66.65	65.62	64.91	58.14
C_{23+26}	$CH_{2\ (morph)}$ (closer to N)	53.79	53.29	53.38	51.41

*Resonances were assigned by using ^1H–^{13}C HSQC, ^1H–^{13}C HMBC and ^1H–^1H COSY NMR spectra.

2. Crystallographic data collection

Table S1. Crystal Data and Details of Data Collection and Refinement for $[Co^{III}(HL^1)(L^1)](NO_3)_2{\cdot}H_2O$ **(1)**, $[Co^{III}(HL^3)(L^3)](NO_3)_2$**(3)**, $[Fe^{III}(L^2)_2]NO_3$ (**4**), $[Fe^{III}(HL^3)(L^3)](NO_3)_2$ (**6**).

compound	$[Co^{III}(HL^1)(L^1)](NO_3)_2{\cdot}H_2O$	$[Co^{III}(HL^3)(L^3)](NO_3)_2$	$[Fe^{III}(L^2)_2]NO_3$	$[Fe^{III}(HL^3)(L^3)](NO_3)_2$
empirical formula	$C_{40}H_{51}CoN_{12}O_{11}S_2$	$C_{38}H_{49}CoN_{12}O_{10}S_2$	$C_{42.65}$ $H_{54.60}FeN_{11}O_{7.65}S_2$	$C_{36}H_{41}FeN_{12}O_8S_2$
fw	998.97	956.94	963.74	889.78
space group	$P2_1/c$	$P2_1/n$	$P2_1/n$	$P2_1/n$
a, Å	19.607(3)	17.659(4)	16.8583(16)	17.7730(5)
b, Å	17.3104(15)	9.9034(13)	13.8926(7)	9.9333(2)
c, Å	15.5505(15)	24.975(7)	21.7744(17)	24.8488(7)
α, °				
β, °	109.389(10)	95.42(2)	107.706(7)	95.264(2)
γ, °				
V [Å^3]	4978.7(11)	4348.1(17)	4858.1(7)	4368.4(2)
Z	4	4	4	4
λ [Å]	0.71073	0.71073	0.71073	0.71073
ρ_{calcd}, g cm^{-3}	1.333	1.462	1.318	1.353
cryst size, mm^3	0.17 × 0.11 × 0.04	0.25 × 0.12 × 0.03	0.20 × 0.12 × 0.01	0.30 × 0.14 × 0.02
T [K]	100(2)	100(2)	100(2)	100(2)
μ, mm^{-1}	0.495	0.561	0.456	0.503
$R_1{}^a$	0.0526	0.0416	0.0851	0.0325
$wR_2{}^b$	0.1289	0.1053	0.2308	0.0762
GOF^c	0.918	0.929	0.953	0.945
CCDC no.	2354137	2354138	2354139	2354140

$^aR_1 = \Sigma||F_o| - |F_c||/\Sigma|F_o|$. ^{b} $wR_2 = \{\Sigma[w(F_o^2 - F_c^2)^2]/\Sigma[w(F_o^2)^2]\}^{1/2}$. ^{c}GOF $= \{\Sigma[w(F_o^2 - F_c^2)^2]/(n - p)\}^{1/2}$, where n is the number of reflections and p is the total number of parameters refined.

Table S2. Crystal Data and Details of Data Collection and Refinement for $[Ni^{II}(L^1)]Cl{\cdot}CH_3OH$, $[Zn^{II}(L^1)Cl]{\cdot}CH_3OH$ and $[Pd^{II}(HL^1)Cl]Cl$

compound	$[Ni^{II}(L^1)]Cl\cdot CH_3OH$	$[Zn^{II}(L^1)Cl]\cdot CH_3OH$	$[Pd^{II}(HL^1)Cl]Cl$	$[H_2L^1]NO_3$
empirical formula	$C_{21}H_{28}ClN_5NiO_3S$	$C_{20}H_{24}ClN_5O_2SZn$	$C_{20}H_{27}Cl_2N_5O_3PdS$	$C_{20}H_{24}N_6O_5S$
fw	524.70	499.32	576.81	460.51
space group	*Pnn*2	$P\bar{1}$	*I*2/*c*	$P2_1/n$
a, Å	20.022(3)	9.5512(16)	13.9557(12)	13.9111(13)
b, Å	8.8256(10)	9.8642(17)	15.1993(13)	8.5430(6)
c, Å	17.135(2)	12.120(2)	24.0558(18)	18.0419(18)
α, °		87.313(14)		
β, °		80.712(13)	102.430(5)	92.547(8)
γ, °		70.063(12)		
V [Å^3]	3027.9(7)	1059.3(3)	4983.0(7)	2142.0(3)
Z	4	2	8	4
λ [Å]	0.71073	0.71073	0.71073	1.54178
ρ_{calcd}, g cm^{-3}	1.151	1.565	1.538	1.428
cryst size, mm^3	0.70 × 0.075 × 0.075	0.20 × 0.08 × 0.035	0.13 × 0.08 × 0.02	0.30 × 0.14 × 0.02
T [K]	296(2)	100(2)	100(2)	100(2)
μ, mm^{-1}	0.824	1.412	1.069	1.744
$R_1{}^a$	0.0301	0.0565	0.0369	0.0700
$wR_2{}^b$	0.0603	0.1678	0.0687	0.2007
GOF^c	1.022	1.088	0.854	0.985
CCDC no.	2354141	2354142	2354143	2354144

^{a} $R_1 = \Sigma||F_o| - |F_c||/\Sigma|F_o|$. ^{b} $wR_2 = \{\Sigma[w(F_o{}^2 - F_c{}^2)^2]/\Sigma[w(F_o{}^2)^2]\}^{1/2}$. ^{c} GOF $= \{\Sigma[w(F_o{}^2 - F_c{}^2)^2]/(n - p)\}^{1/2}$, where *n* is the number of reflections and *p* is the total number of parameters refined.

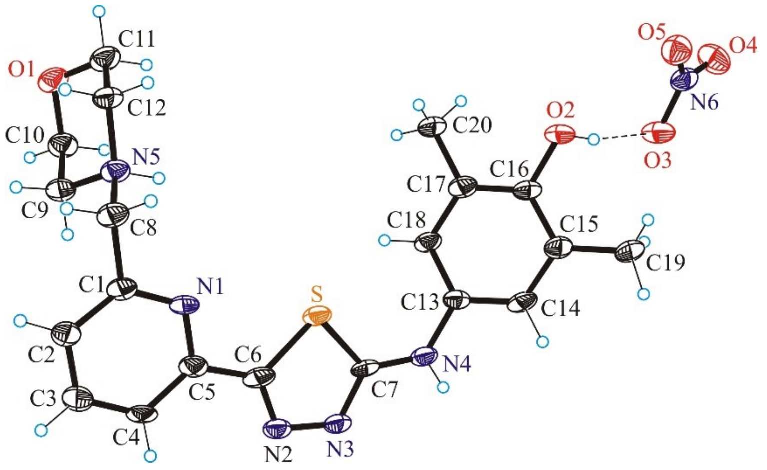

Figure S11. ORTEP view of $[H_2L^1]NO_3$

3. Solution Stability Studies

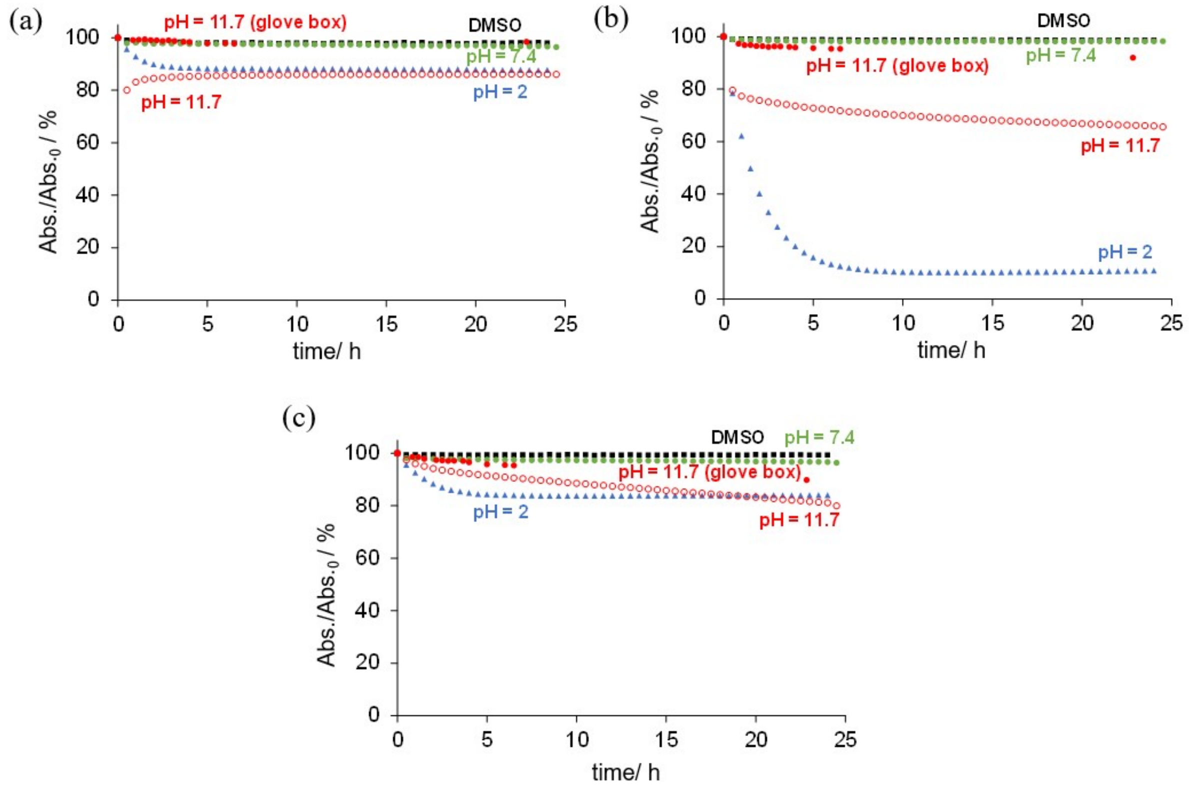

Figure S12. Changes of absorbance values measured for (a) **HL¹**, (b) **HL²** and (c) **HL³** in DMSO (■), at pH 2 (▲), at pH 7.4 (●), at pH 11.7 in glove box (●) and in a normal cuvette (○) over time at the absorbance maximum; {c_{ligand} = 50 μM; ℓ = 1 cm; t = 25.0 °C}.

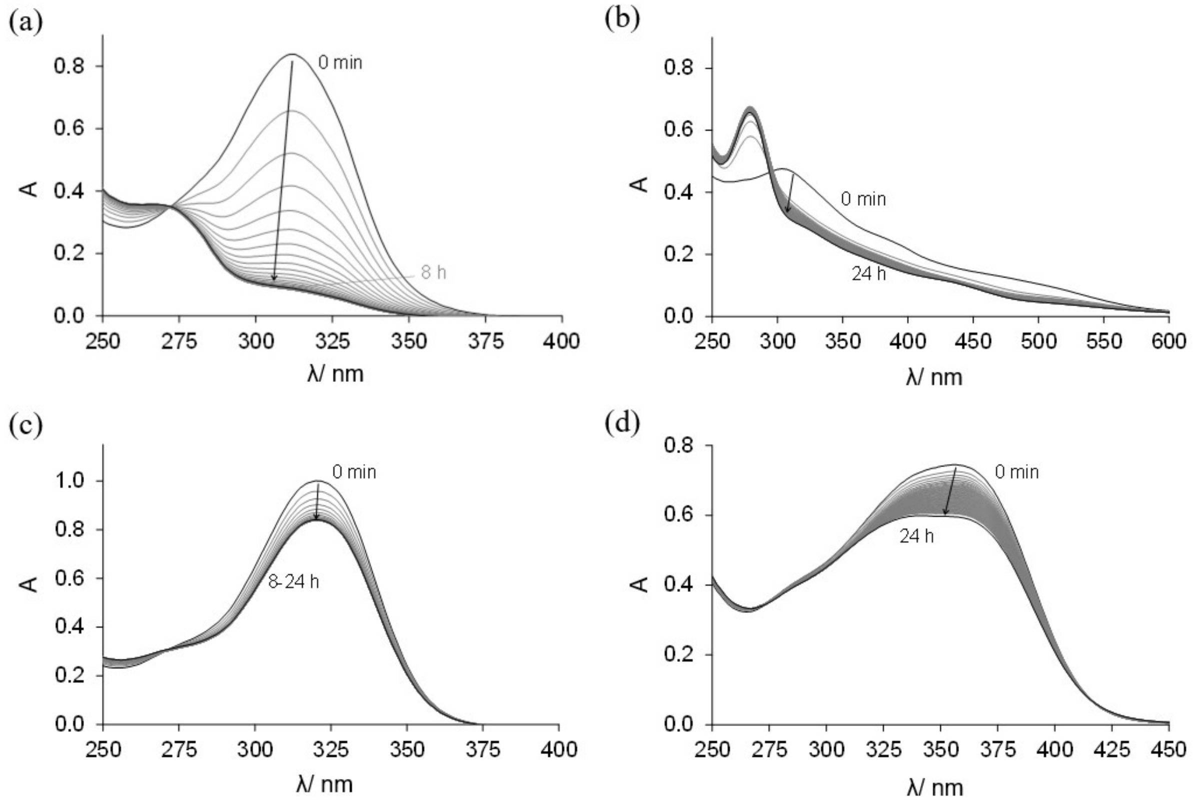

Figure S13. UV–vis absorption spectra recorded for **HL²** at (a) pH 2 and (b) pH 11.7, and for **HL³** at (c) pH 2 and (d) pH 11.7 over time; {c_{ligand} = 50 μM; ℓ = 1 cm; t = 25.0 °C}.

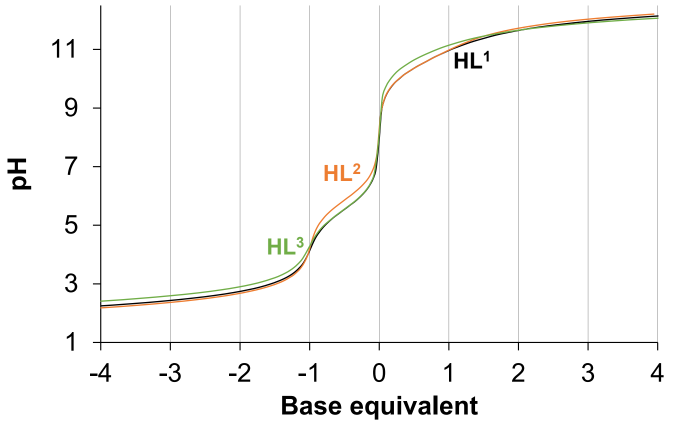

Figure S14. pH-potentiometric titration curves of the studied proligands **HL¹–HL³** in 30% (*v*/*v*) DMSO/H_2O. Base equivalent: $(n_{\text{KOH}} - n_{\text{HCl}})$ / n_{ligand}; {ℓ = 1 cm; I = 0.1 M (KCl); t = 25.0 °C}.

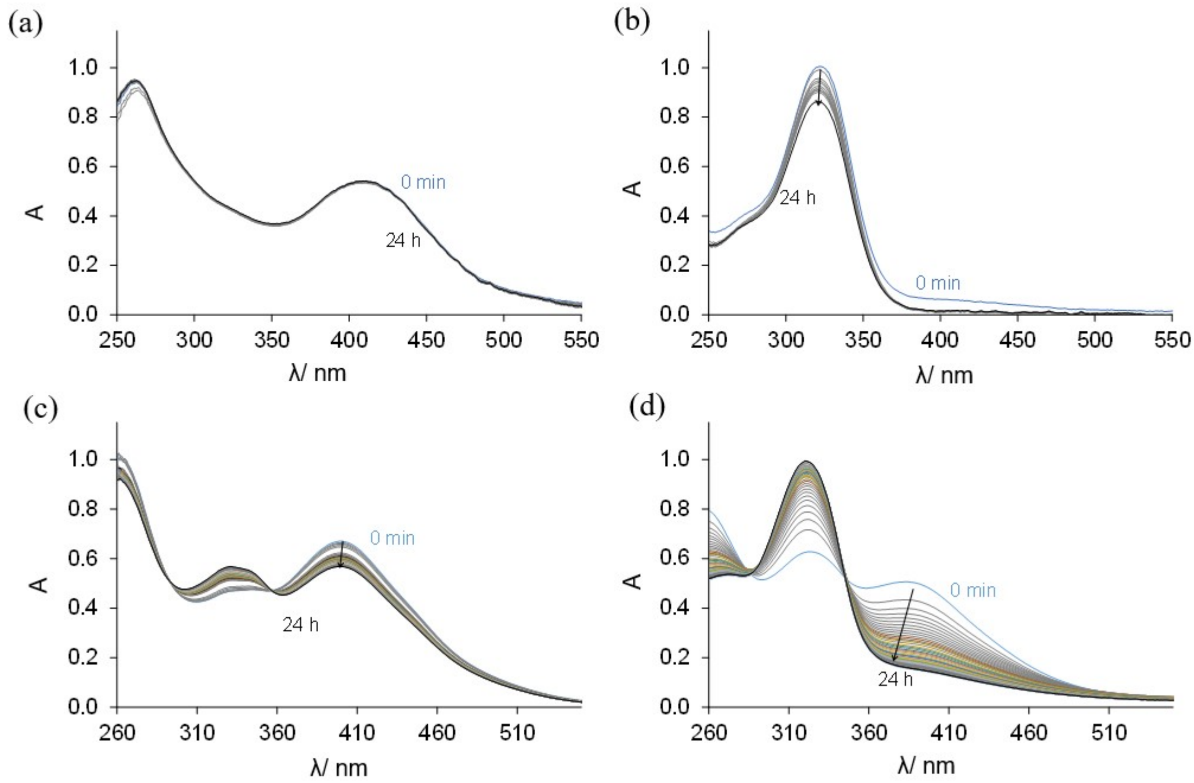

Figure S15. UV–vis absorption spectra recorded for (a) complex **2** at pH 2 and for complex **5** (b) at pH 2, (c) complex **2** in DMSO, and (d) complex **2** at pH 7.4 over time; {$c_{complex}$ = 21 μM (**2**) or 23 μM (**5**); ℓ = 1 cm; t = 25.0 °C}.

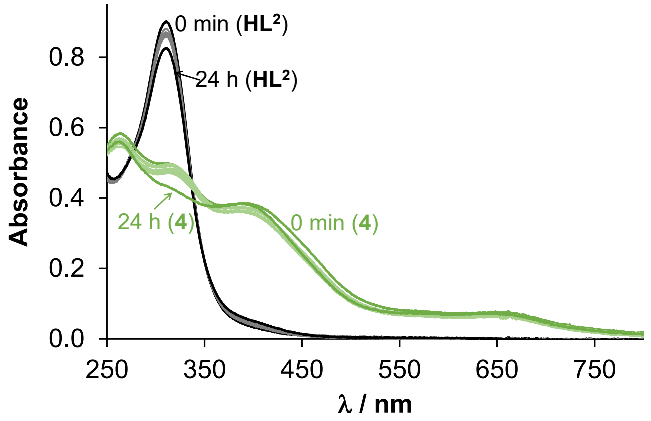

Figure S16. UV–vis absorption spectra recorded for complex **4** (green lines) and **HL²** (black lines) at pH 11.7 over time in the glove box; {c = 23 μM; ℓ = 1 cm; t = 25.0 °C}.

4. Spectroelectrochemistry

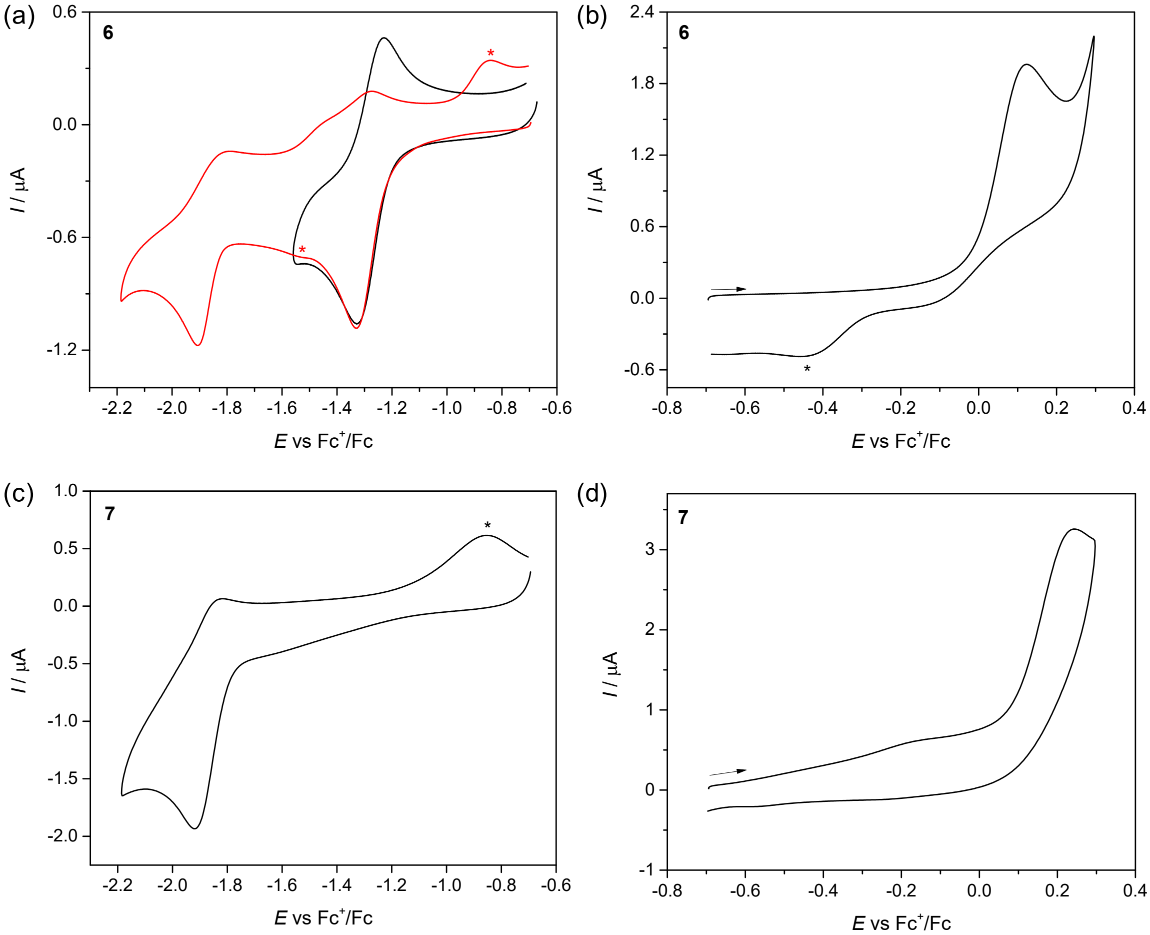

Figure S17. Cyclic voltammograms of the nickel complex **6** (a) in the cathodic and (b) in the anodic part, as well as of the zinc(II) complex **7** (c) in the cathodic and (d) in the anodic part in DMSO/nBu$_4$NPF$_6$ (Pt working electrode, scan rate: 100 mV s^{-1}). Asterisk indicates the follow up products formed upon reduction and oxidation, respectively.

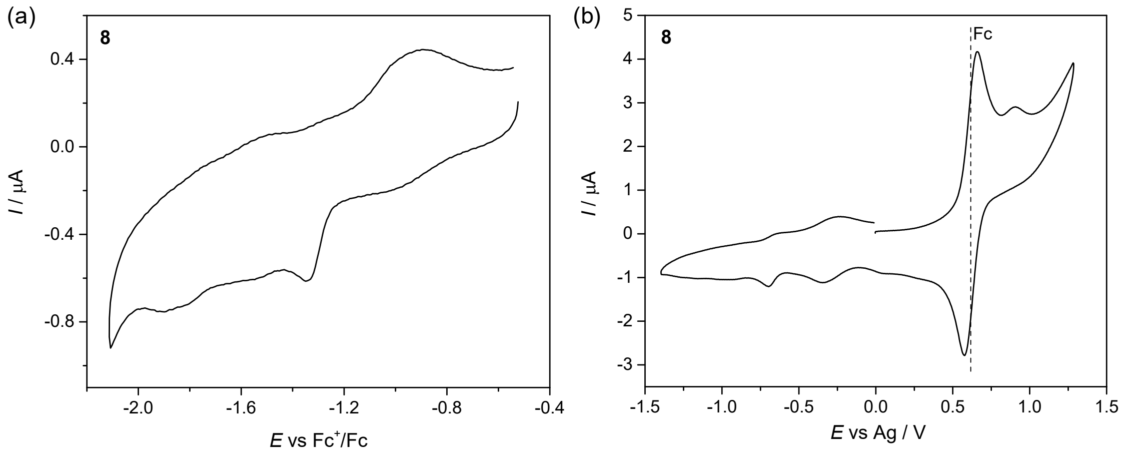

Figure S18. Cyclic voltammograms of palladium(II) complex **8** (a) in the cathodic part and (b) in both the anodic and cathodic parts in the presence of ferrocene in DMSO/$n$$Bu_4NPF_6$ (Pt working electrode, scan rate: 100 mV s^{-1}).

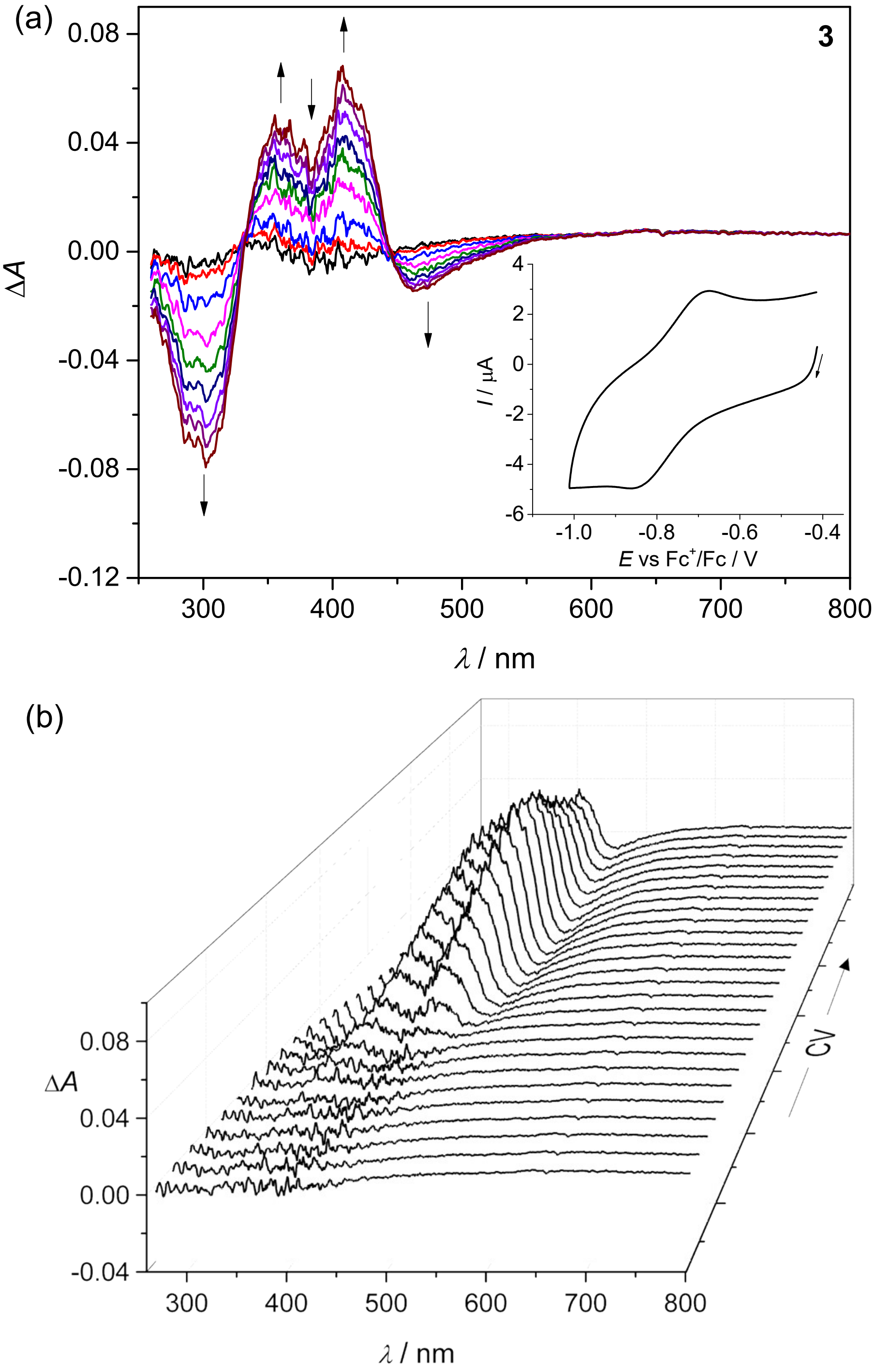

Figure S19. (a) Difference UV–vis–NIR spectra detected simultaneously during the *in situ* reduction of **3** in $n\mathrm{Bu_4NPF_6}$/DMSO in the region of the first cathodic peak (forward scan). Inset: respective cyclic voltammogram (Pt-microstructured honeycomb working electrode, scan rate v = 10 mV s^{-1}). (b) Difference UV–vis–NIR spectra taken during cyclic voltametric scan of **3** shown in 3D mode.

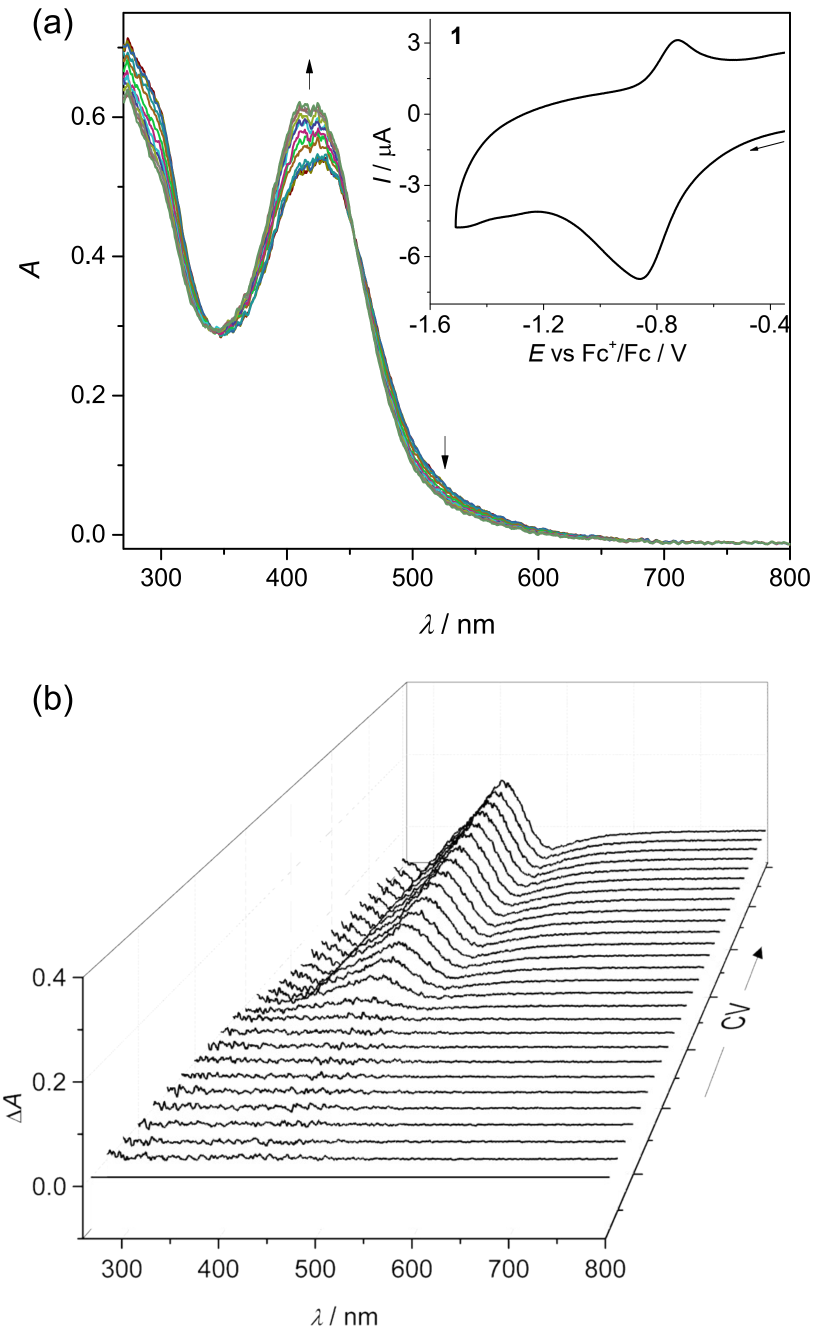

Figure S20. (a) UV–vis–NIR spectra detected simultaneously upon the *in situ* reduction of **1** in nBu$_4$NPF$_6$/DMSO in the region of the first cathodic peak; inset: respective cyclic voltammogram (Pt-microstructured honeycomb working electrode, scan rate v = 10 mV s^{-1}); (b) Difference UV–vis–NIR spectra taken upon cyclic voltametric scan of **1** shown in 3D mode.

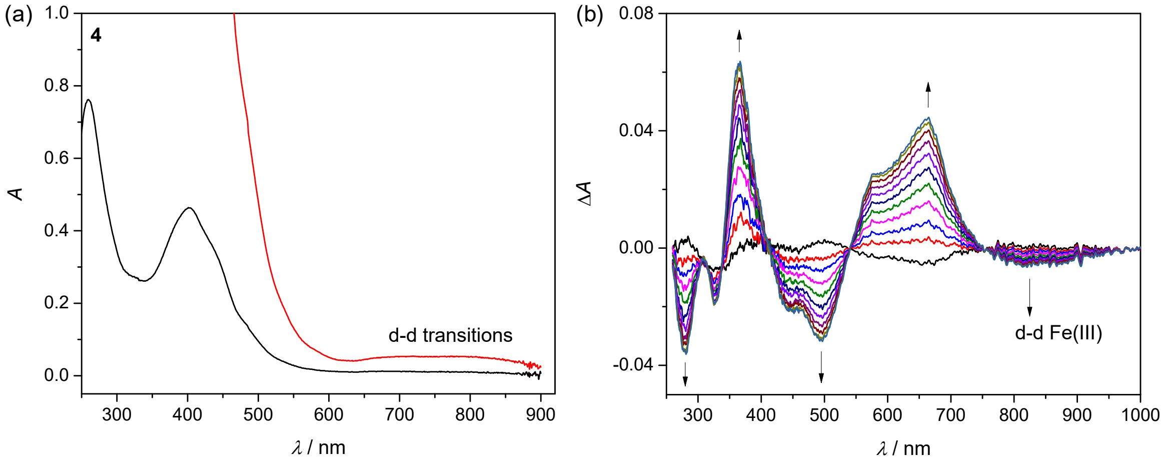

Figure S21. (a) UV–vis–NIR spectra of **4** in DMSO; (b) Spectroelectrochemistry of **4** in nBu$_4$NPF$_6$/DMSO in the region of the first cathodic peak - evolution of difference UV–vis–NIR spectra measured simultaneously upon forward scan (Pt-microstructured honeycomb working electrode, scan rate $v = 10$ mV s^{-1}).

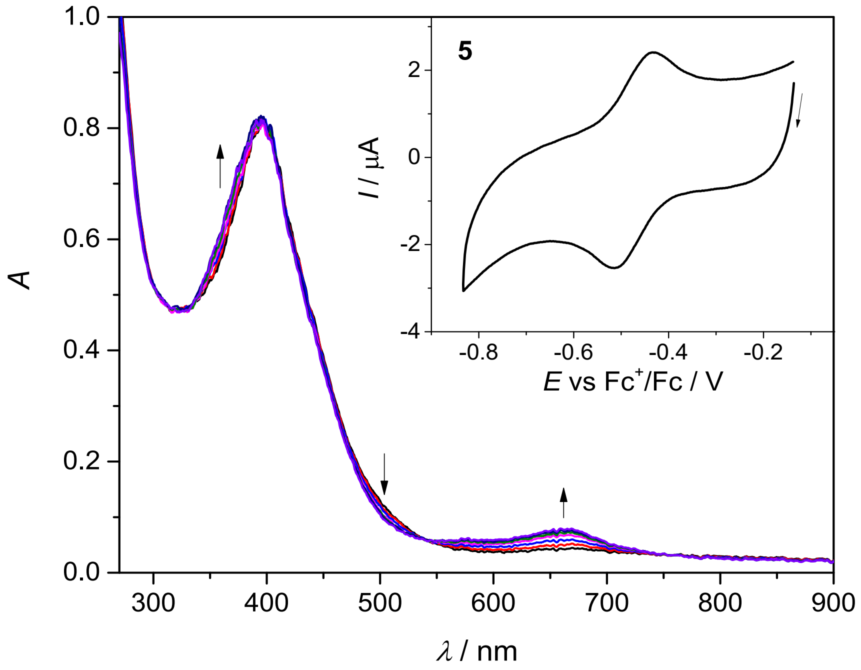

Figure S22. UV–vis–NIR spectra measured simultaneously upon the *in situ* reduction of **5** in nBu$_4$NPF$_6$/DMSO in the region of the first cathodic peak (forward scan); inset: respective cyclic voltammogram (Pt-microstructured honeycomb working electrode, scan rate $v = 10$ mV s^{-1}).

5. NCI-60 One-Dose Screen

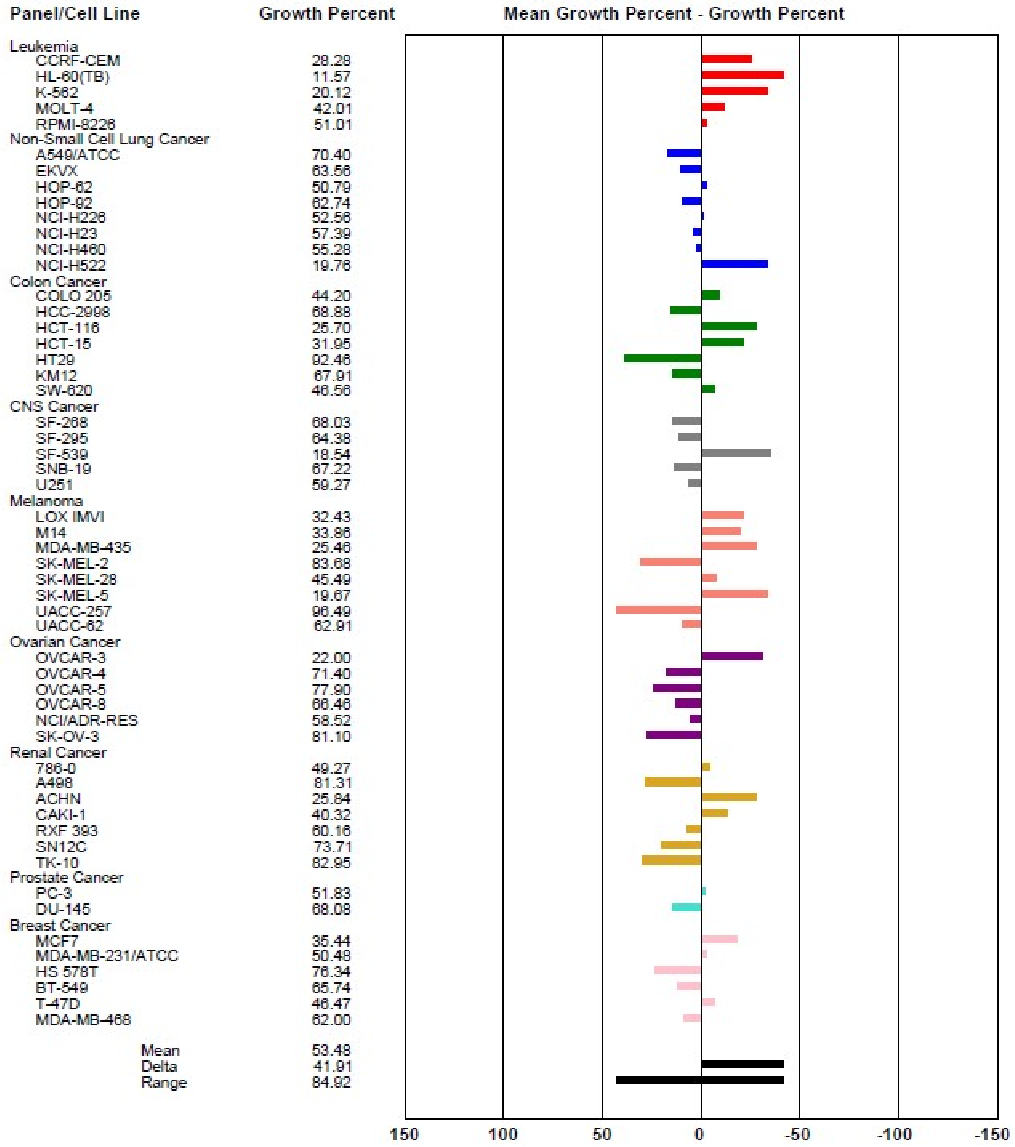

Figure S23. One dose mean graph for **HL[1]**.

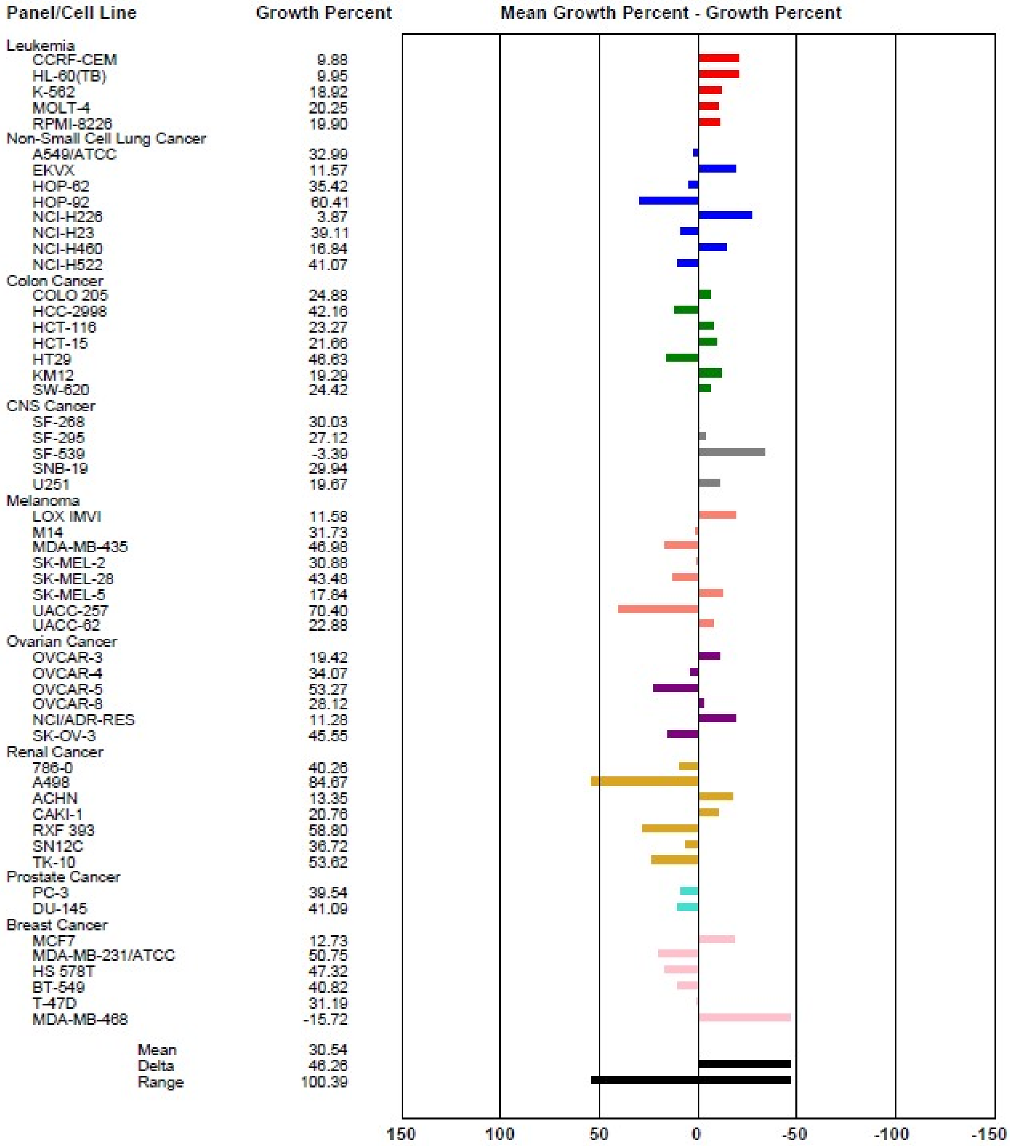

Figure S24. One dose mean graph for **HL²**.

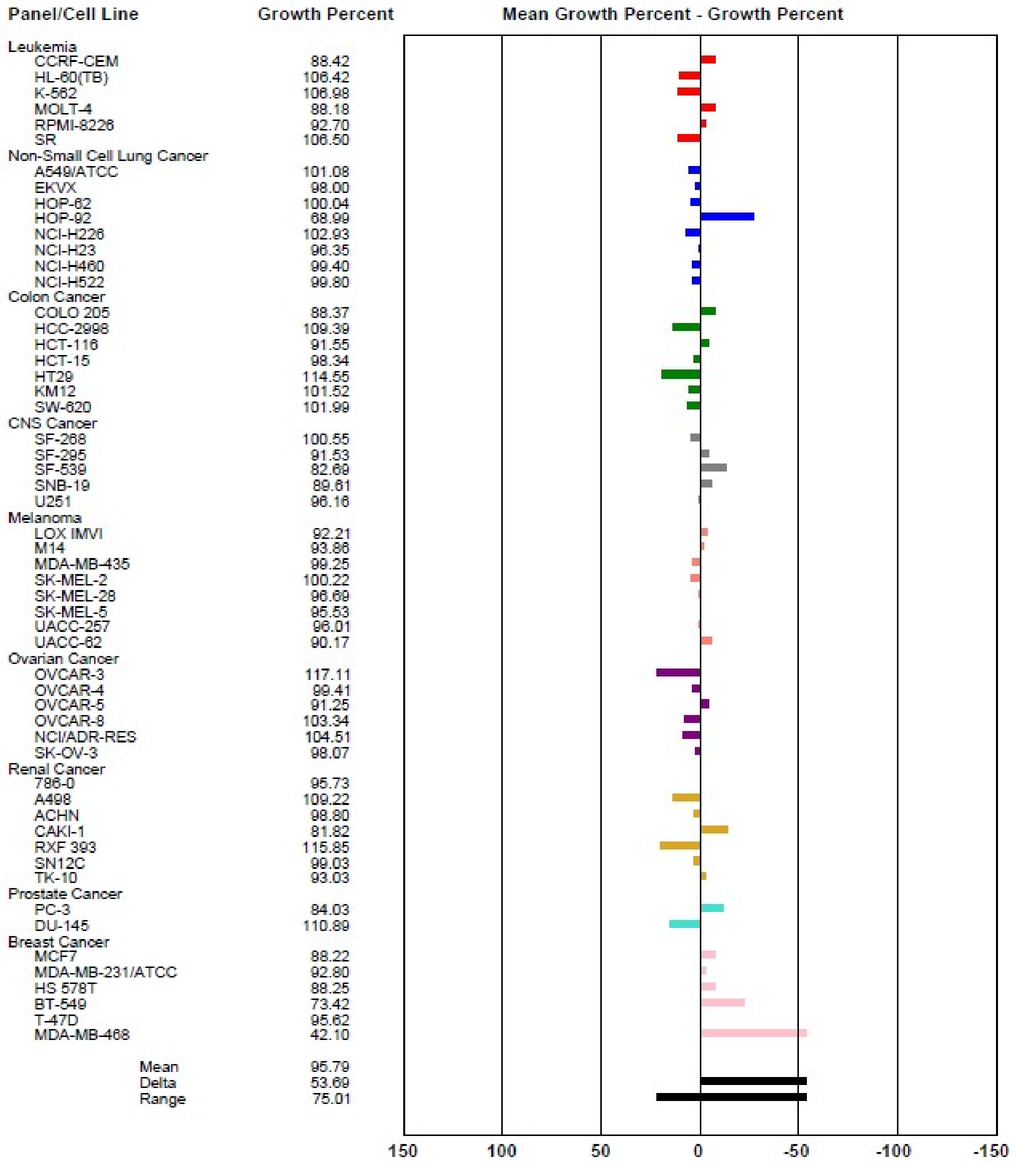

Figure S25. One dose mean graph for **3**.

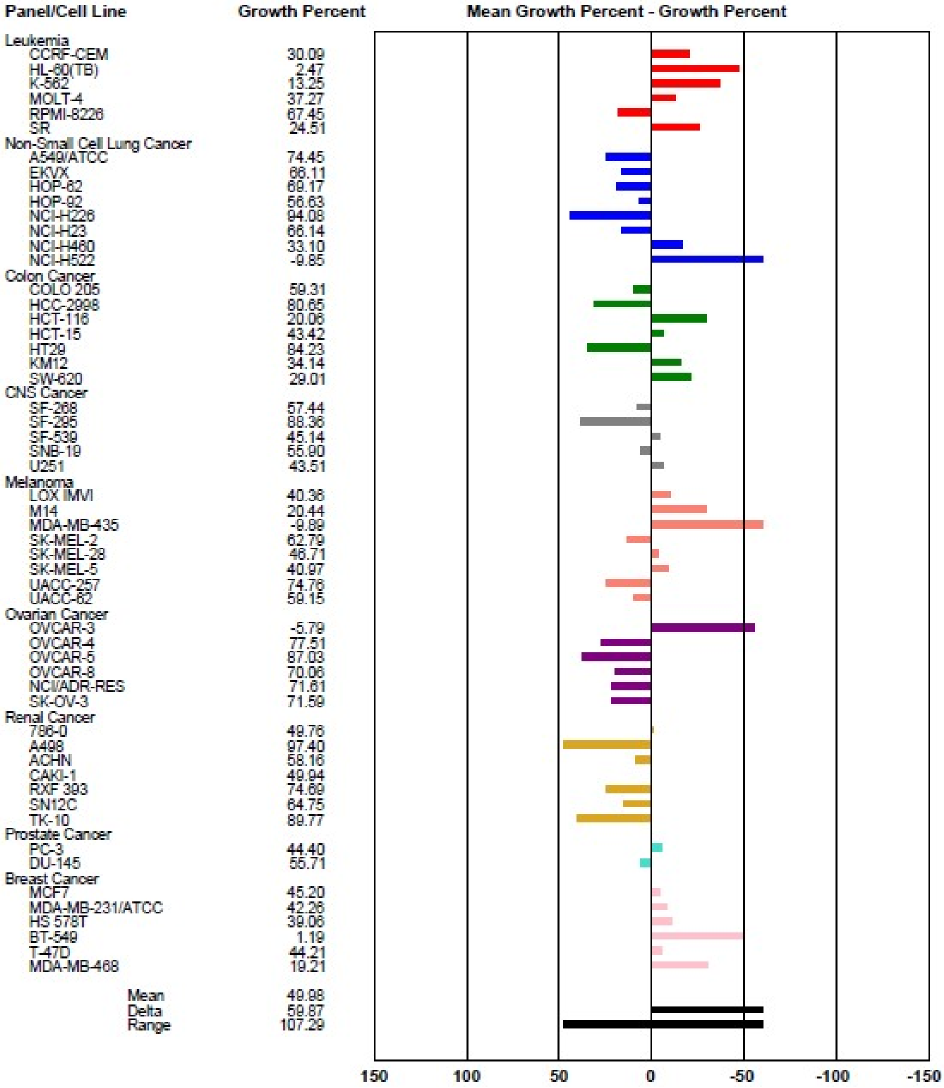

Figure S26. One dose mean graph for **6**.

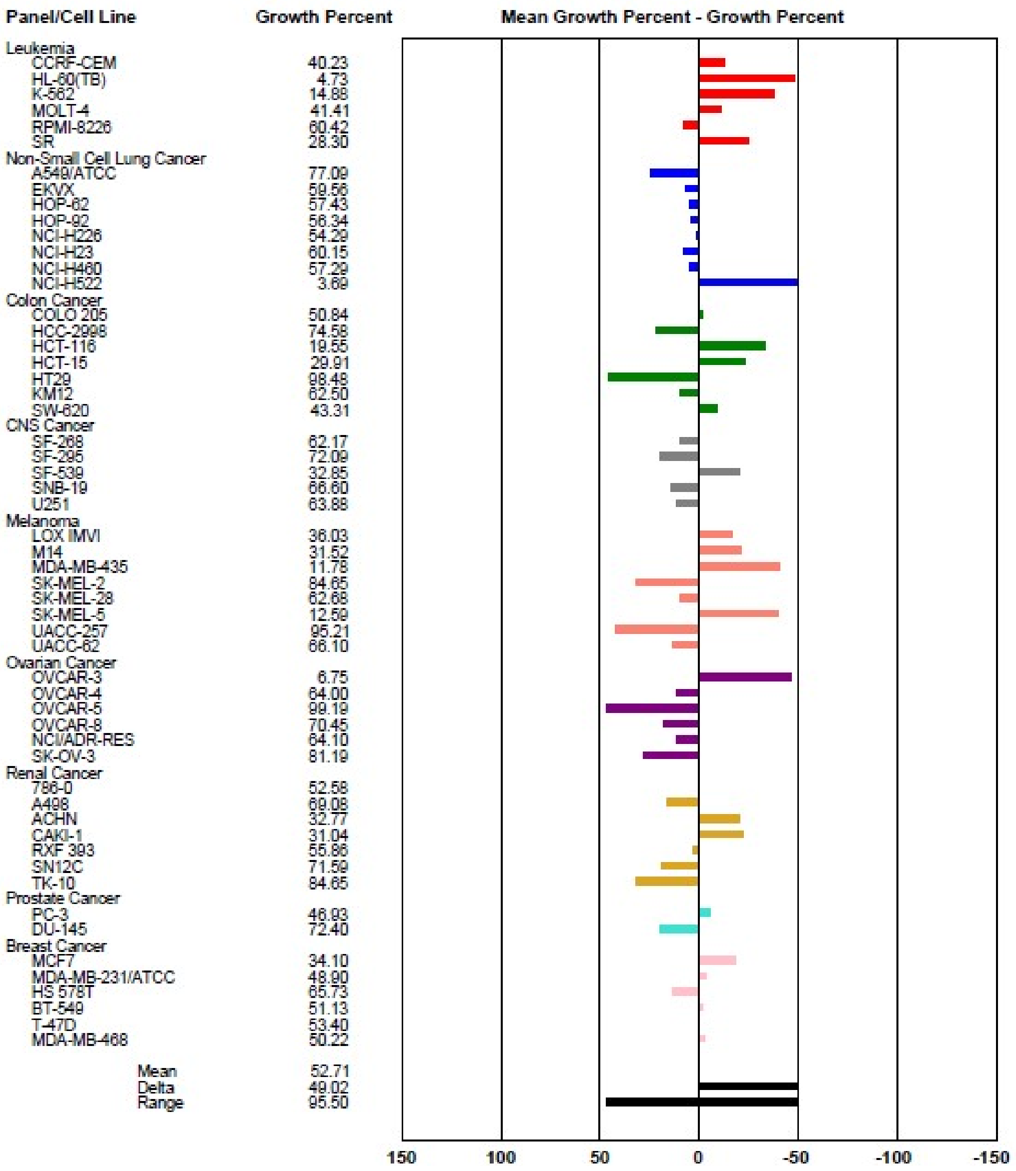

Figure S27. One dose mean graph for **7**.

Panel/Cell Line	Growth Percent
Leukemia	
CCRF-CEM	6.14
HL-60(TB)	24.85
K-562	26.90
MOLT-4	23.58
RPMI-8226	37.50
SR	30.54
Non-Small Cell Lung Cancer	
A549/ATCC	52.02
EKVX	72.38
HOP-62	50.38
HOP-92	82.78
NCI-H226	58.82
NCI-H23	74.34
NCI-H460	53.44
NCI-H522	-81.87
Colon Cancer	
COLO 205	51.50
HCC-2998	81.90
HCT-116	18.05
HCT-15	36.09
HT29	78.34
KM12	62.48
SW-620	26.51
CNS Cancer	
SF-268	66.41
SF-295	80.54
SF-539	69.45
SNB-19	69.20
U251	49.23
Melanoma	
LOX IMVI	37.91
M14	20.18
MDA-MB-435	9.85
SK-MEL-2	65.76
SK-MEL-28	55.25
SK-MEL-5	31.25
UACC-257	66.88
UACC-62	64.43
Ovarian Cancer	
OVCAR-3	6.66
OVCAR-4	57.10
OVCAR-5	82.86
OVCAR-8	61.54
NCI/ADR-RES	71.20
SK-OV-3	67.05
Renal Cancer	
786-0	32.80
A498	74.57
ACHN	57.90
CAKI-1	43.66
RXF 393	53.13
SN12C	78.02
TK-10	87.32
Prostate Cancer	
PC-3	52.10
DU-145	62.80
Breast Cancer	
MCF7	19.92
MDA-MB-231/ATCC	57.40
HS 578T	74.15
BT-549	30.11
T-47D	24.66
MDA-MB-468	-1.96
Mean	48.11
Delta	129.98
Range	169.19

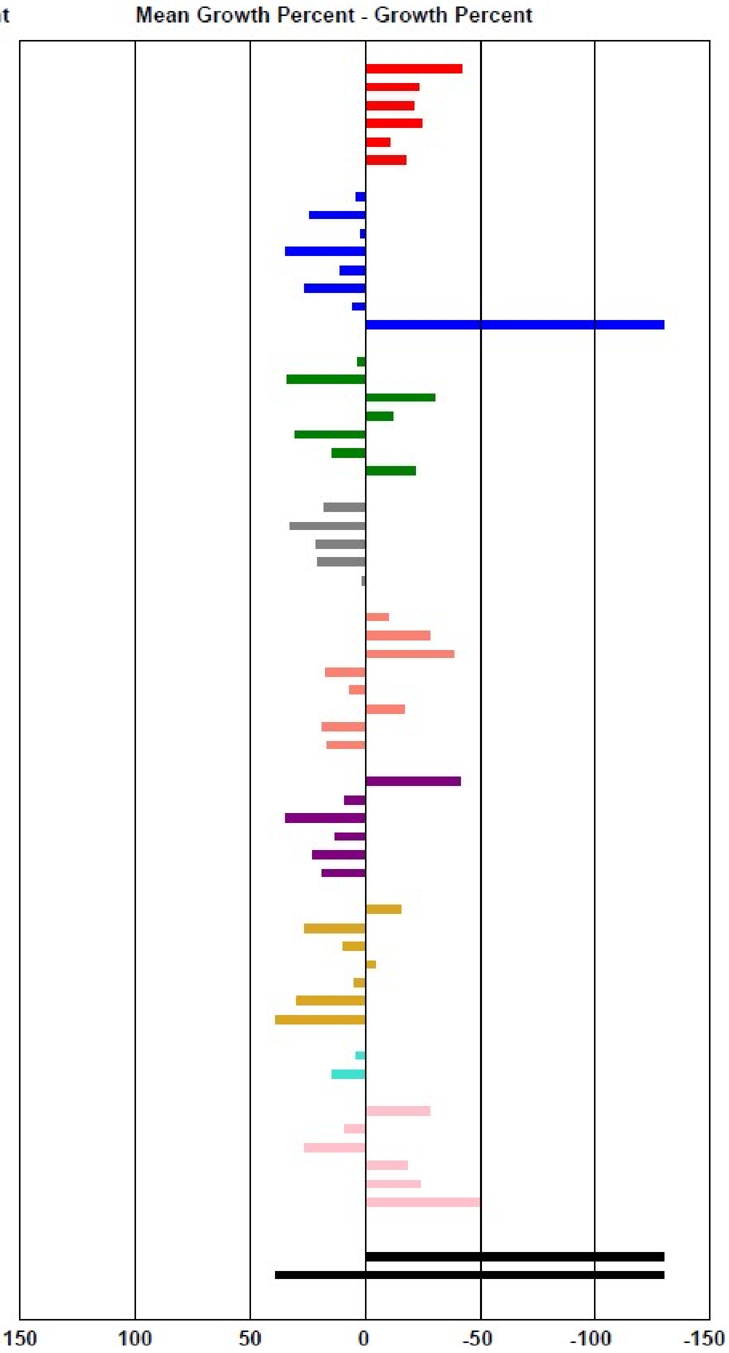

Figure S28. One dose mean graph for **8**.

6. NCI-60 5-Dose Screen

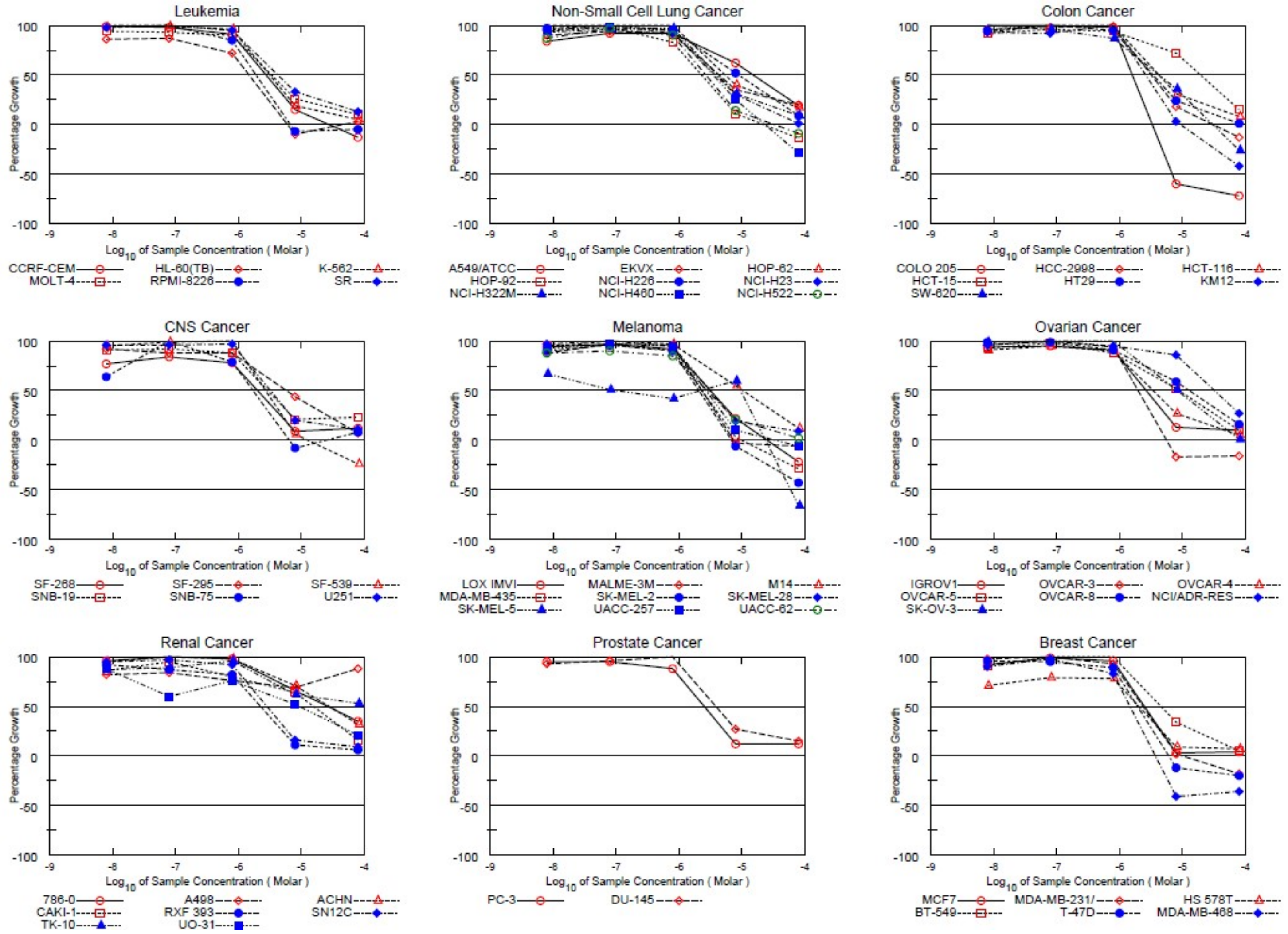

Figure S29. Dose response curves for **1**.

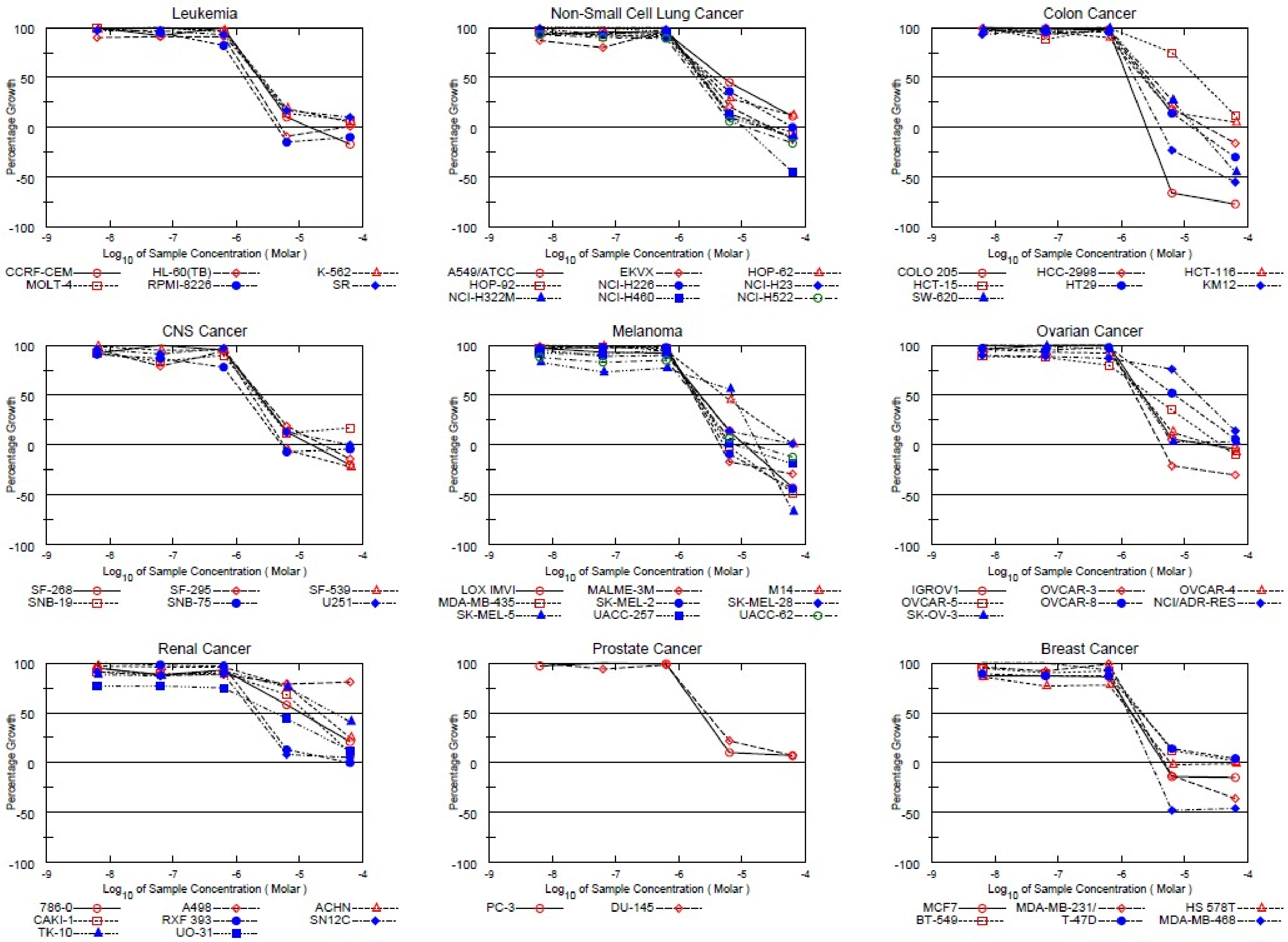

Figure S30. Dose response curves for **2**.

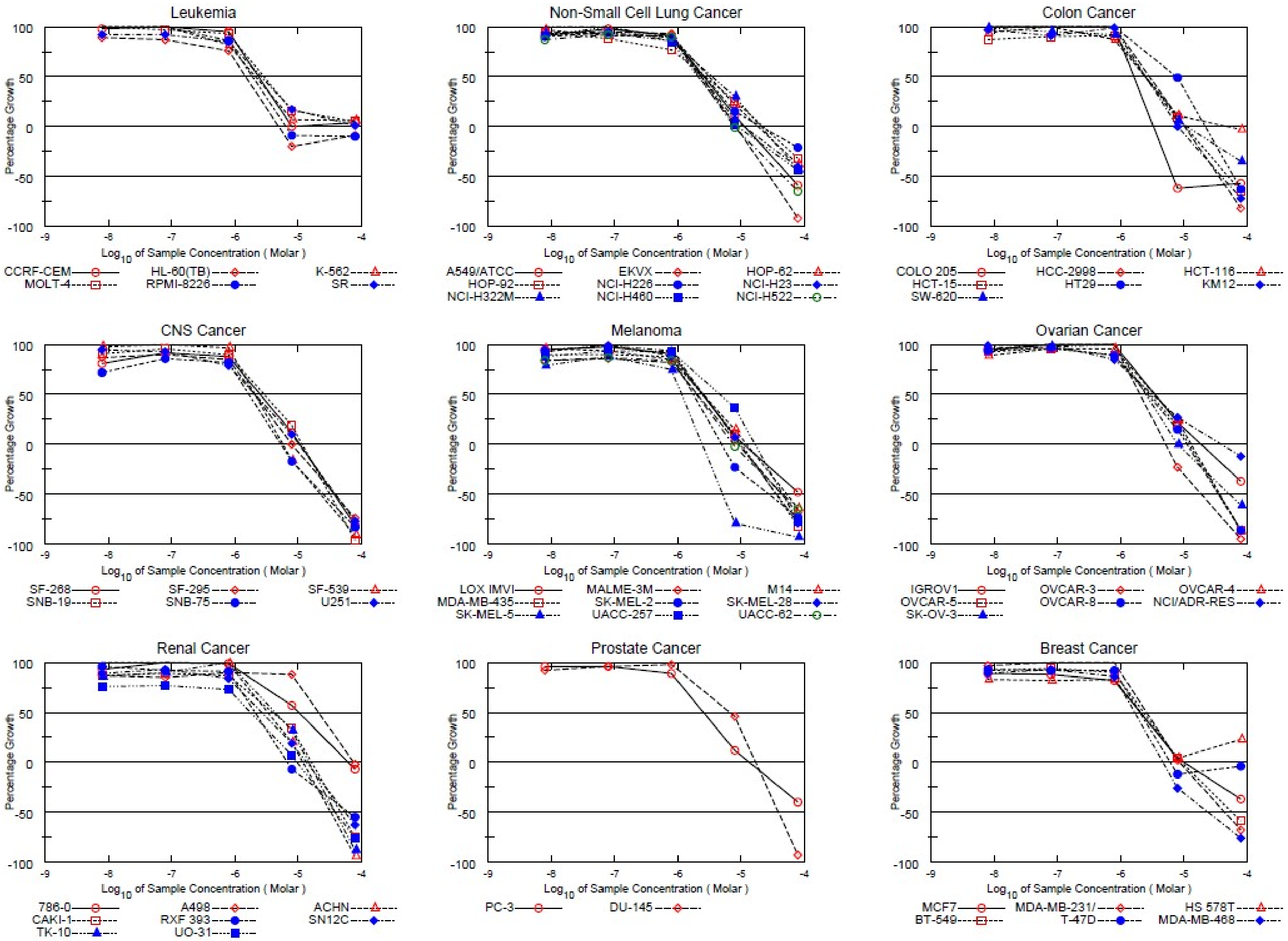

Figure S31. Dose response curves for **4**.

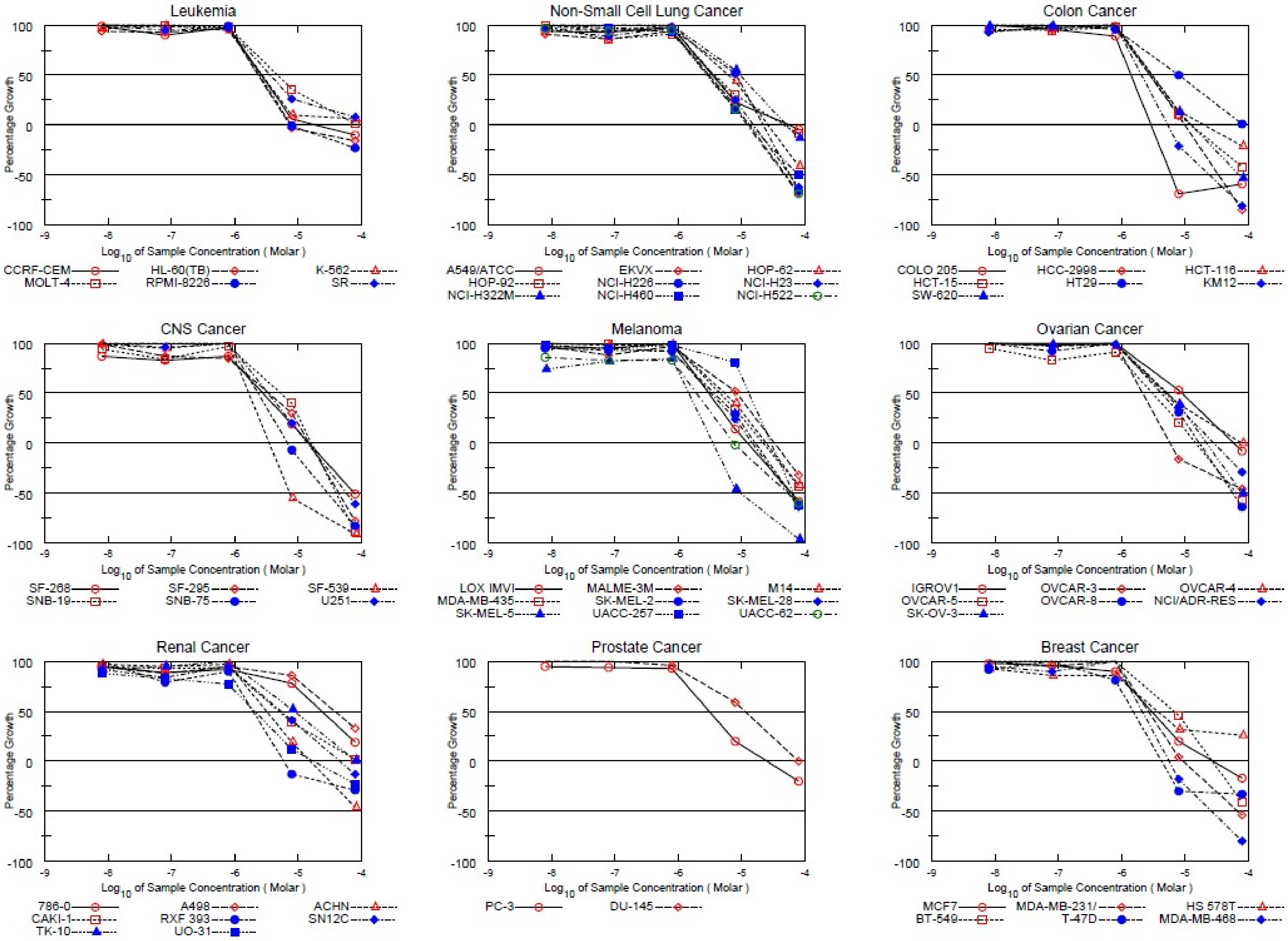

Figure S32. Dose response curves for **5**.

7. Molecular docking

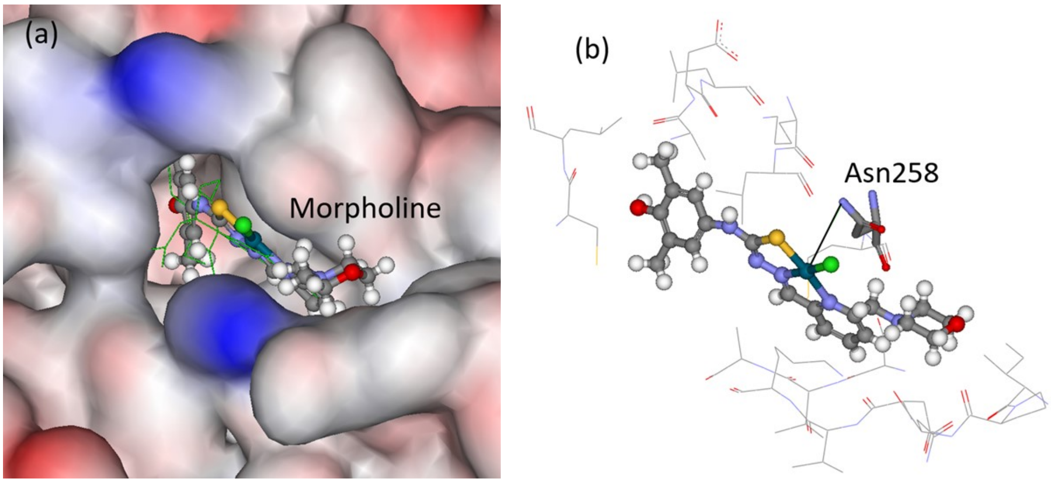

Figure S33. (a) The docked pose of **8** (ball-and-stick) in the colchicine site of tubulin. The co-crystalized ligand (LOC) is shown in line format (green), its hydrogen atoms are not shown for clarity. The protein surface is rendered with blue color depicting regions with a partial positive charge on the surface, red color depicting regions with a partial negative charge and grey showing neutral areas. (b) The predicted binding of complex **8**, amino acids within 5 Å are shown in line format. Complex formation of the amide side chain of βAsn258 with the Pd(II) could occur as shown in this docking image (black solid line, 3.7 Å).
